# Salicylate metal-binding isosteres as fragments for metalloenzyme inhibition†

**DOI:** 10.1039/d1sc06011b

**Published:** 2022-01-21

**Authors:** Moritz K. Jackl, Hyeonglim Seo, Johannes Karges, Mark Kalaj, Seth M. Cohen

**Affiliations:** Department of Chemistry and Biochemistry, University of California San Diego 9500 Gilman Drive La Jolla CA 92093-0358 USA scohen@ucsd.edu

## Abstract

Metalloenzyme inhibitors typically share a common need to possess a metal-binding pharmacophore (MBP) for binding the active site metal ions. However, MBPs can suffer from physicochemical liabilities, impeding the pharmacological properties and drug-likeliness of inhibitors. To circumvent this, problematic features of the MBP can be identified and exchanged with isosteric replacements. Herein, the carboxylic and hydroxyl group of the salicylic acid MBP were replaced and a total of 27 salicylate metal-binding isosteres (MBIs) synthesized. Of these 27 MBIs, at least 12 represent previously unreported compounds, and the metal-binding abilities of >20 of the MBIs have not been previously reported. These salicylate MBIs were examined for their metal-binding features in model complexes, physicochemical properties, and biological activity. It was observed that salicylate MBIs can demonstrate a range of attractive physicochemical properties and bind to the metal in a variety of expected and unexpected binding modes. The biological activity of these novel MBIs was evaluated by measuring inhibition against two Zn^2+^-dependent metalloenzymes, human glyoxalase 1 (GLO1) and matrix metalloproteinase 3 (MMP-3), as well as a dinuclear Mn^2+^-dependent metalloenzyme, influenza H1N1 N-terminal endonuclease (PA_N_). It was observed that salicylate MBIs could maintain or improve enzyme inhibition and selectivity. To probe salicylate MBIs as fragments for fragment-based drug discovery (FBDD), an MBI that showed good inhibitory activity against GLO1 was derivatized and a rudimentary structure–activity relationship was developed. The resulting elaborated fragments showed GLO1 inhibition with low micromolar activity.

## Abstract 2

Metal-binding isosteres (MBIs) of salicylic acid have been developed for metalloenzyme drug development.

## Graphical abstract

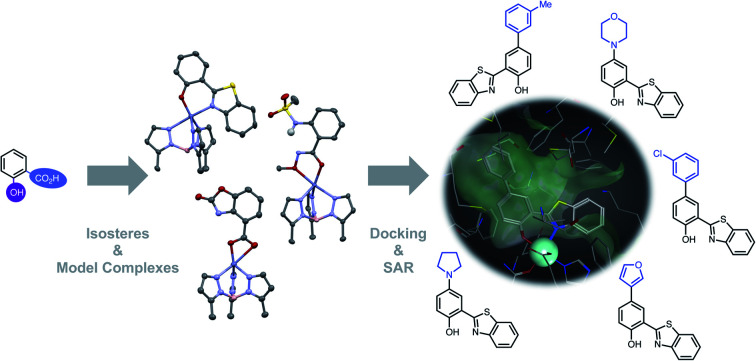

## Introduction

Metalloenzymes have been found to play important roles in the propagation of various diseases, ranging from microbial infections to cancer, which makes them an important class of targets for drug development.^1^ Fragment-based drug discovery (FBDD)^2–5^ strategies have been successfully used to develop potent inhibitors against metalloenzymes. Starting from a metal-binding pharmacophore (MBP), fragment growth strategies have been applied to identify hits from which lead-like compounds with good potency and selectivity can be obtained.^6–8^

Unfortunately, many MBPs are highly polar molecules that contain functional groups that can suffer from pharmacological liabilities, such as poor membrane permeability, challenging solubility, or toxicity. An attractive approach to circumvent these problems is to install isosteric replacements. Problematic functional groups can be exchanged with alternative, isosteric groups with improved properties while maintaining similar biological activity. This strategy is frequently applied in modern medicinal chemistry and drug development and it has been demonstrated that isosteric replacement can improve solubility, lipophilicity, cell permeability, and reduce toxicity. Furthermore, isosteres can be used to produce novel compositions of matter for intellectual property purposes.^9–11^

Recently, isosteric replacement has been applied to MBPs for metalloenzyme inhibition. In a proof-of-concept study, the carboxylic acid moiety of picolinic acid was exchanged for known carboxylic acid isosteres to obtain a library of metal-binding isosteres (MBIs). These picolinic acid MBIs showed a broad range of physicochemical properties while maintaining similar metal-binding properties and biological activity as the parent MBP. This demonstrated the potential value of the MBI concept to allow for modulation of properties in the development of more drug-like metalloenzyme inhibitors by FBDD.^12,13^

Herein, MBIs of salicylic acid, a known metal binding ligand and metalloenzyme inhibitor, were prepared.^14^ A library of 27 salicylate MBIs was designed, synthesized, and studied for metal-binding and physicochemical properties. Furthermore, their inhibitory activity against two Zn^2+^-dependent metalloenzymes – human glyoxalase I (GLO1) and matrix metalloproteinase-3 (MMP-3) – and a dinuclear Mn^2+^-dependent metalloenzyme – influenza H1N1 N-terminal endonuclease (PA_N_) – was investigated. A selected salicylate MBI, which showed good inhibitory activity against GLO1, was computationally docked into the enzyme active site and a rudimentary SAR study was performed. It was demonstrated that salicylate MBIs not only cover a wide range of physicochemical properties and chemical space but also showcase various metal-binding modes, opening new avenues for metalloenzyme inhibitor development. It was observed that salicylate MBIs not only maintain, but also can surpass the parent salicylic acid MBP in terms of metalloenzyme inhibition and selectivity in select cases. Finally, it was shown that salicylate MBI fragments are easy to derivatize, which makes them strong candidates for lead development and rational drug design.

## Results and discussion

### MBI library design and synthesis

**Fig. 1:**
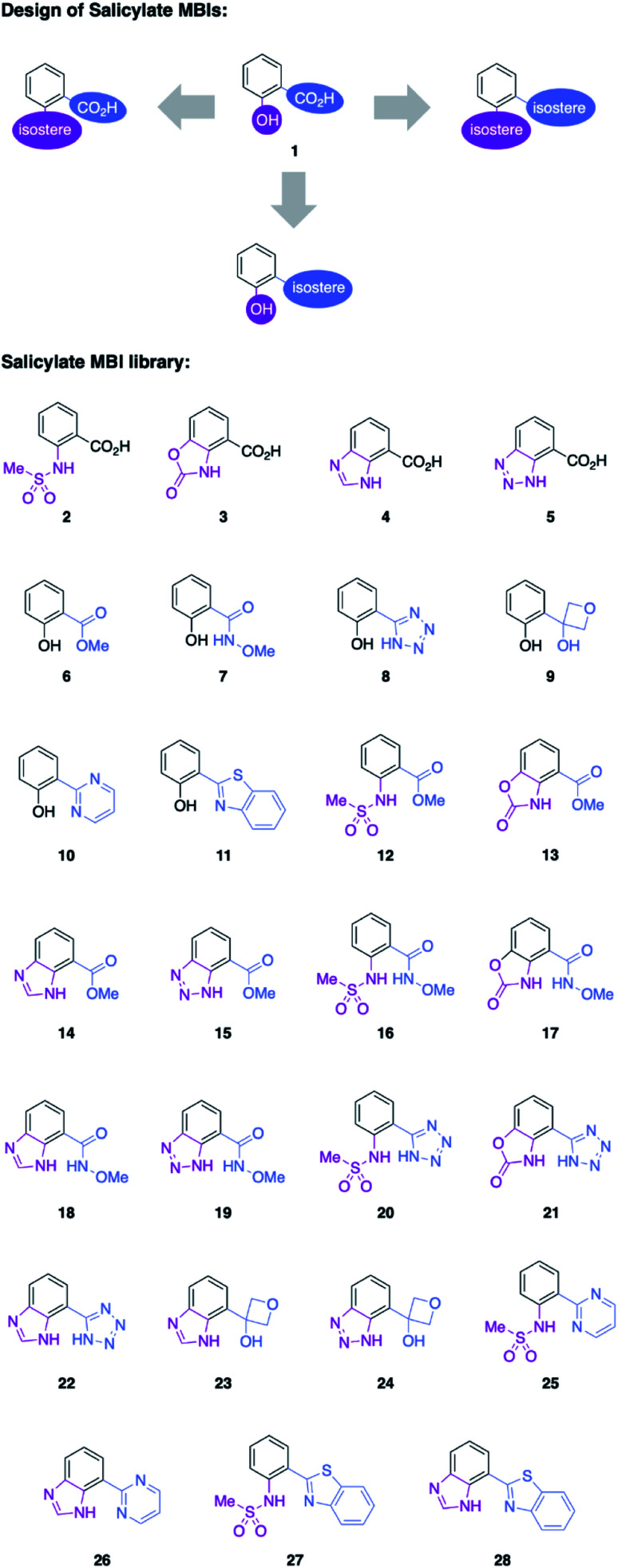
Top: Salicylic acid (1) and conceptual approach to salicylic acid metal-binding isosteres (MBIs). Bottom: MBIs of salicylic acid. The hydroxyl group (purple) and the carboxylic acid group (blue) were replaced with isosteres to obtain 27 new MBIs.

Salicylic acid 1 is known to bind to various metal ions and salicylic acids have been reported as metalloenzyme inhibitors.^14^ Two sites of the molecule – the hydroxyl group and the carboxylic acid group – are generally involved in metal binding. Consequently, these functional groups were the target of isosteric replacement (Fig. 1).

Similar to a previous study on picolinic acid MBIs, the carboxylic acid moiety was substituted by a hydroxamic acid ester, tetrazole, and oxetane.^13^ These isosteric replacements were assumed to bind to the metal in their deprotonated, anionic form. Furthermore, methyl ester, pyrimidine, and benzothiazole were identified as neutral metal coordinators and potential carboxylic acid isosteres. To date, hydroxyl group isosteres have not been widely reported in the context of MBIs. Inspired by the literature on classical, non-metal-binding isosteres, several functional groups, including sulfonamides, carbamates, imidazoles, and triazoles were selected as isostere replacements for the hydroxyl group.^9–11^

MBIs with one or both of these isosteric replacements were designed and synthesized, resulting in a library of 27 MBIs (Fig. 1). Salicylate MBIs 2, 4, and 6 were purchased from commercial suppliers, while all other MBIs were prepared in a few steps from widely available starting materials using established synthetic methods (see ESI†). Esters and hydroxamic acid esters were derived from carboxylic acids by simple functional group interconversions, whereas tetrazoles were prepared from nitriles by 1,3-dipolar cycloaddition. Oxetanes were accessible from halides by lithiation/addition to 3-oxetanone, pyrimidines and benzothiazoles were synthesized using cross-coupling approaches. Sulfonamides were prepared by reaction of anilines with methylsulfonyl chloride, carbamates by annulation of amino alcohols with 1,1′-carbonyldiimidazole (CDI), and benzotriazoles by a diazotization/ring-closing protocol. Importantly, 12 out of 27 MBIs (compounds 16–19, 21–28) are new, unreported molecules. This indicates that by simply applying the principle of isosters to salicylic acid, new chemical space and new compositions of matter can be readily obtained.

### Synthesis and characterization of model complexes

**Fig. 2:**
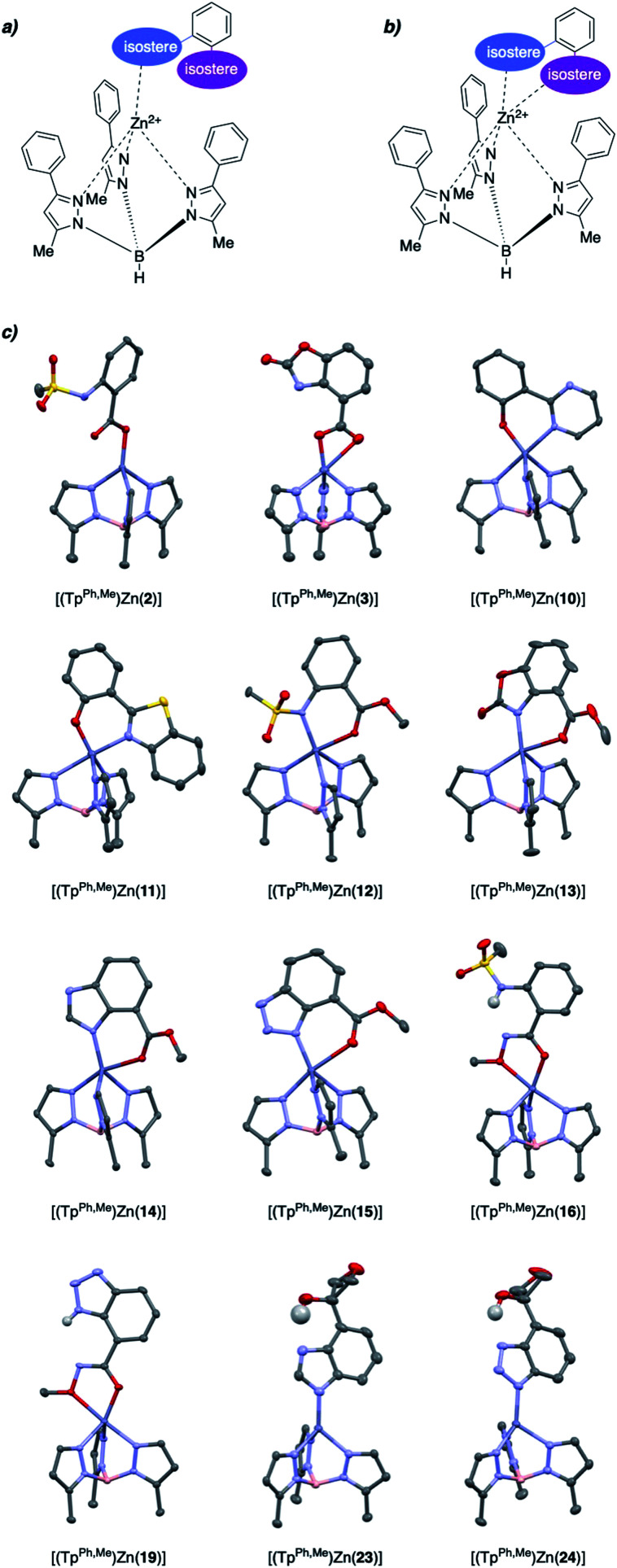
(a) Monodentate and (b) bidentate binding modes for MBIs. (c) Crystal structures of [(Tp^Ph,Me^)Zn(MBI)] model complexes. Hydrogen atoms and phenyl groups on Tp^Ph,Me^ were omitted for clarity except for hydrogen atoms involved in intramolecular hydrogen bonds. Color code: carbon = gray, nitrogen = blue, oxygen = red, sulfur = yellow, boron = pink, zinc = dark blue.

To evaluate the structural features of metal binding by the salicylate MBIs, [(Tp^Ph,Me^)Zn(MBI)] (Tp^Ph,Me^ = hydrotris(3,5-phenylmethylpyrazolyl)borate) model complexes were prepared and characterized by X-ray crystallography (Fig. 2). The [(Tp^Ph,Me^)Zn(MBI)] complex models the tris(histidine) active site of Zn^2+^-dependent metalloenzymes (*e.g.*, matrix metalloproteinases, MMPs) and has been widely used to evaluate the structural characteristics of MBPs and MBIs as potential metalloenzyme inhibitors.^15–17^

Various binding modes were observed with different salicylate MBIs (Fig. 2). While MBIs 10–15 formed 6-membered chelate rings engaging both hydroxyl and carboxylic acid groups (or isosteres) MBIs 2, 3, 16, 19, 23, and 24 bound the Zn^2+^ ion by only one isostere donor in either a monodentate or bidentate fashion.

Both monodentate and bidentate coordination in [(Tp^Ph,Me^)Zn(MBP)] complexes has been observed for salicylate ligands^18^ and it was hypothesized that the differences in binding behaviour observed here can be explained, in part, by charge neutrality. To obtain an overall neutral [(Tp^Ph,Me^)Zn(MBI)] complex, monoanionic MBIs are required, as dianionic MBIs will produce charged species. However, some functional groups, such as carboxylic acids, alcohols, or sulfonamides must be deprotonated (*e.g.*, form anionic ligands) to effectively bind to the metal centre. MBIs that combine two ionizable groups, such as 2, 3, 16, 19, 23, and 24, consequently can only bind as 6-membered chelates when double deprotonated (*i.e.*, dianionic), which disfavours charge neutrality. It should be noted that this is a limitation of the [(Tp^Ph,Me^)Zn(MBI)] model complex and does not necessarily correlate within the context of a metalloenzyme active site. Indeed, several protein crystal structures of dianionic MBPs (*e.g.*, catechol ligands) bound to active site metal ions in metalloenzymes have been reported.^19–21^

An interesting observation was made for hydroxamic ester MBIs 16 and 19 (Fig. 2). These MBIs bind to the Zn^2+^ ion *via* the deprotonated hydroxamic ester moiety forming 5-membered chelates. The deprotonated hydroxamic ester is present in the enol form and presumably stabilized by intramolecular hydrogen bonding from the sulfonamide and benzotriazole groups, respectively. Considering the widespread use of hydroxamic acids as MBPs in metalloenzyme inhibitors,^22^ this observation could inspire the use of hydroxamic esters for new avenues in the development of MBIs and metalloenzyme inhibitors. For example, the hydroxamic ester moiety could be easily elaborated by applying more complex *O*-substituted hydroxylamines. This would enable additional interactions with enzyme pockets near the metal centre, which cannot be readily achieved with conventional hydroxamic acid MBPs.

Internal hydrogen bonds were also observed for oxetane MBIs 23 and 24 (Fig. 2). These MBIs are bound to the metal with a deprotonated nitrogen atom of the imidazole or triazole ring. Another nitrogen of the heteroaromatic moiety engages in internal hydrogen bonding with the aliphatic hydroxyl group adjacent to the oxetane. Apart from stabilization, this also rigidifies the molecules and could enable secondary interactions of the oxetane moiety with an enzyme pocket. Overall, this rich variations in metal binding modes among the 27 novel MBIs, serves to highlight the fact that only five of these compounds have previously characterized metal complexes (compounds 4, 6, 8, 11, 14), further emphasizing their utility as novel moieties for metal coordination.

### Physicochemical analysis

In addition to gaining an understanding about the metal coordinating properties of the salicylate MBIs by using [(Tp^Ph,Me^)Zn(MBI)] model complexes, the physicochemical properties of the MBIs were also studied. The acidity (p*K*_a_) and lipophilicity (log *P*) were measured using potentiometric methods and the lipophilicity at physiological pH (log *D*_7.4_) was calculated from these two values (Table 1).^23–25^

**Table 1:** Measured Physicochemical Properties of Salicylic Acid (1) and select MBIs

Compound	p*K*_a_	log *P*	log *D*_7.4_
1	2.82, >12	2.18	−2.40
2	3.04, 10.8	1.39	−2.97
3	3.65, 10.29	1.28	−2.47
4	3.04, 5.49, 12.79	−1.40	−2.09
5	3.49, 9.29	1.38	−2.37
6	9.8	2.19	2.19
7	7.23, 12	0.85	0.49
8	4.63, 10.92	1.54	−0.46
9	9.38	0.43	0.42
12	8.86	1.51	1.49
13	8.78	1.24	1.22
14	4.51, 12.32	1.73	1.73
15	7.96	1.31	1.21
16	7.15, 11.31	0.12	−0.33
17	7.58, 12.51	0.40	0.18
18	4.23, 8.40, >12	0.05	0.01
19	6.99, 9.93	0.75	0.22
20	3.62, 9.52	1.18	−0.43

Overall, a wide range of acidities and lipophilicities was observed among the MBIs. Except for MBI 4 (Table 1, entry 4), which is an ampholyte and presumably exist as the zwitterion, all log *P* values were in the range of 0–3. MBIs 2–5 (Table 1, entries 2–5) were rather acidic (p*K*_a,1_ ∼3) and are deprotonated at physiological pH, which explains the high polarity of these MBIs at this pH (log *D*_7.4_ approx. −2). When the carboxylic moiety was exchanged for a less acidic isostere, the lipophilicity at physiological pH increased significantly. Interestingly, this was also observed for the tetrazole MBIs – despite the rather low p*K*_a_ of this functional group (p*K*_a,1_ ∼4), lipophilicities at physiological pH were in the range log *D*_7.4_ ∼0. This highlights that by careful selection and combination of isosteric groups, MBIs with tailor-made physicochemical properties can be designed.

Unfortunately, the physicochemical properties of benzothiazole MBIs (11, 27, 28) could not be measured with the applied method due to the poor aqueous solubility of these compounds. Literature data of structurally related compounds (*e.g.*, 4-(benzo[*d*]thiazol-2-yl)phenol, log *P* = 1.86, determined by a HPLC based method) suggests higher lipophilicity compared to other salicylate MBIs. This was confirmed by computationally calculated physicochemical data (Tables S2 and S3†). The calculated log *P* and log *D*_7.4_ values of MBIs 11 and 28 were the highest of the entire MBI library, which potentially makes these MBIs interesting candidates for central nervous system applications.^26–28^

### MBI screening

To evaluate if the salicylate MBIs could serve as starting points for future FBDD campaigns, the MBI library was screened for inhibitory activity against human glyoxalase I (GLO1) at fragment concentrations of 100 μM (Table 2). GLO1 is a Zn^2+^-dependent metalloenzyme and is associated with the propagation of several diseases.^29–31^ The catalytic Zn^2+^ is coordinated to four amino acids (Gln33, Glu99, Glu172, His126) and is in proximity to two hydrophobic pockets.^1,32^ Previous studies (unpublished) found salicylic acid 1 to be a weak GLO1 inhibitor (IC_50_ >50 μM). This result was confirmed in our screening and around half of the salicylate MBIs showed similar or better inhibitory activity compared to salicylic acid. MBIs 11, 22, and 27 inhibited GLO1 significantly better, with 22 showing 50% inhibition and 11 almost complete inhibition at 100 μM. The good inhibitory activity of 11 compared to the other salicylate MBIs could be rationalized by hydrophobic (and potentially π–π) interactions of the benzothiazole moiety with the hydrophobic pockets in proximity to the catalytic Zn^2+^.^8,33^ This indicates that isosteric replacements in MBPs could not only maintain, but also improve enzyme binding for metalloenzyme inhibition.

**Table 2:** Thermoplot of enzyme activity of MBIs against GLO1 (100 μM), MMP-3 (100 μM) and PA_N_ (100 μM). Standard deviations are given in parentheses. Cells are color-coded by percent inhibition: white (<20%), yellow (20–50%), orange (>50%). The activity of 22 and 28 could not be measured in some cases due to the poor aqueous solubility of the compounds under the assay condition

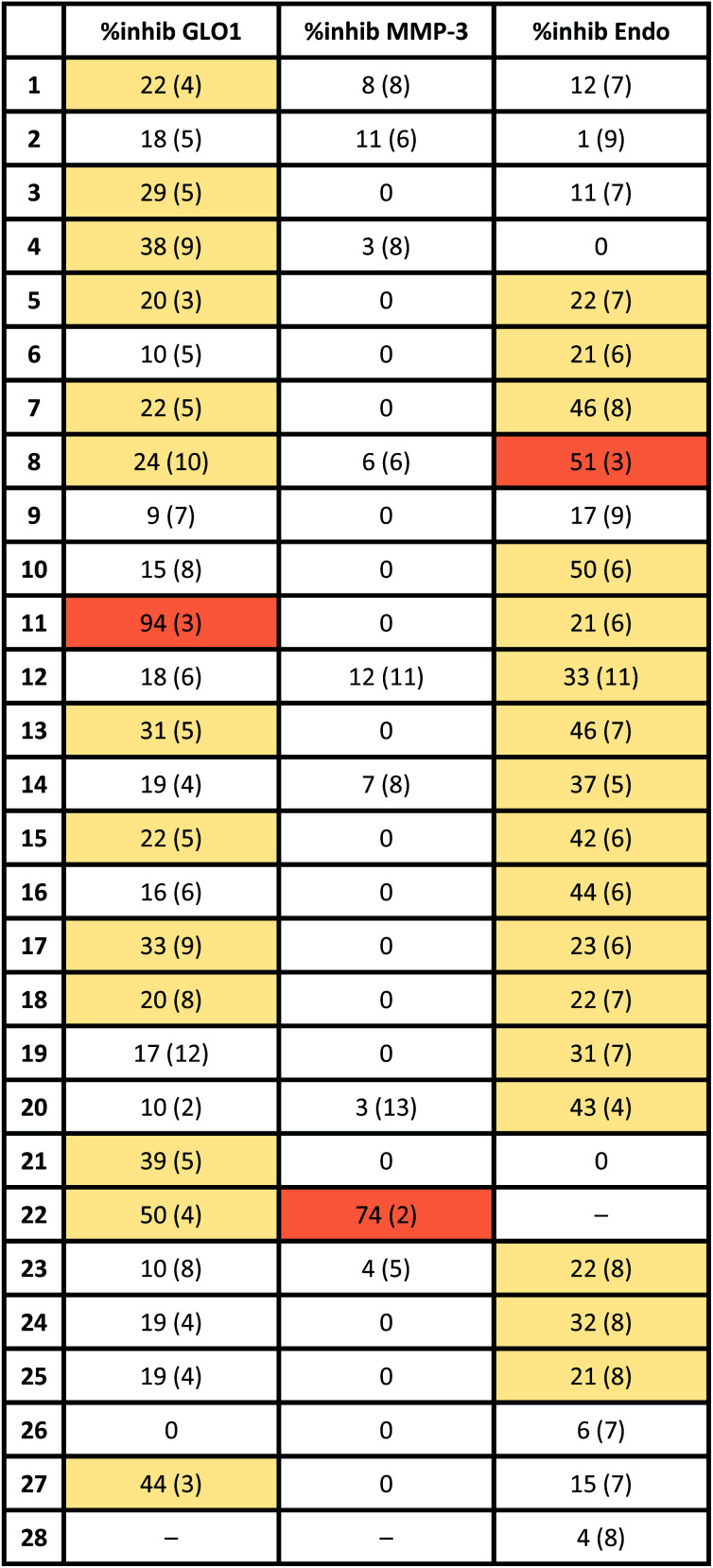

To probe the selectivity of the salicylate MBIs, the library was screened against matrix metalloproteinase-3 (MMP-3) at fragment concentrations of 100 μM (Table 2). MMP-3 is another Zn^2+^-dependent metalloenzyme that is inhibited by a variety of MBPs – however, salicylic acid 1 is not a known MMP-3 inhibitor.^1,14,34,35^ Except for MBI 22, which showed 74% inhibitory activity against MMP-3, the salicylate MBIs showed only very weak or no inhibition at all. These results indicate that salicylate MBIs could serve as selective fragments for the development of more elaborate metalloenzyme inhibitors, even against enzymes that contain common active site metal ions (*e.g.*, Zn^2+^).

Finally, the salicylate MBI library was screened against influenza H1N1 N-terminal PA endonuclease (PA_N_), a dinuclear Mn^2+^-dependent metalloenzyme.^38^ PA_N_ plays a major role in the viral lifecycle and is highly conserved among various influenza strains, which makes it an interesting target for the development of influenza antivirals.^1,7^ While the inhibitory activity of salicylic acid 1 was rather low at a concentration of 100 μM, more than half of the MBIs showed some significant inhibition (Table 2), with MBIs 8 and 10 showing inhibition ≥50%. This highlights that salicylate MBIs are not limited to Zn^2+^-dependent metalloenzymes and could potentially serve as starting points for inhibitor development against a variety of metalloenzyme targets.

### Computational docking and SAR

**Fig. 3:**
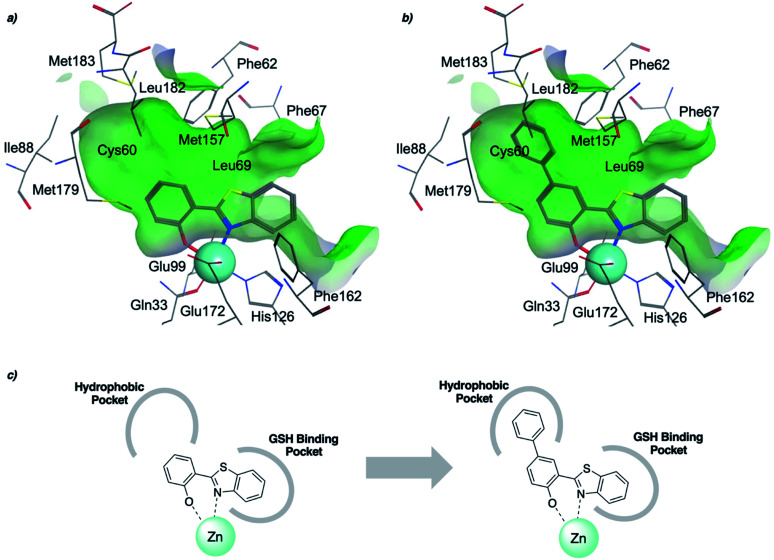
(a) Computational docking of 11 and (b) 11a in the GLO1 active site (PDB: 3VW9). The docking was performed using GOLD and visualized using MOE. Zn^2+^ is represented as a turquoise sphere, active site surface is colored in green with hydrophilic areas in blue. Inhibitor and key amino acid residues are drawn as sticks with carbon atoms in gray, oxygen in red, nitrogen in blue, and sulfur in yellow. (c) Schematic representation of fragment growth and inhibitor enzyme interactions.

To rationalize the high inhibitory activity of 11 against GLO1 and to probe salicylate MBIs as starting points for lead development, computational docking studies were performed. A computational model was built using a reported GLO1 crystal structure (PDB: 3VW9, see ESI† for details).^33^ The docking study confirmed the initial hypothesis – 11 coordinates to the catalytic Zn^2+^ with the benzothiazole nitrogen and the deprotonated hydroxyl group, thereby directing the aromatic moieties towards the hydrophobic pockets of the GLO1 active site (Fig. 3a). The first hydrophobic pocket, also referred to as the glutathione (GSH) binding pocket, consists of Phe67, Leu69, Met157, and Phe162. A second hydrophobic pocket consists of Cys60, Phe62, Ile88, Met179, Leu182, and Met183 residues.^1,8,32^ The benzothiazole moiety of 11 is predicted to occupy a large part of the GSH binding pocket, possibly engaging in hydrophobic or π–π interactions with Phe67, Leu69, and Phe162. However, the second hydrophobic pocket is only partially occupied by 11. To better fill this pocket, a phenyl group was attached to 11 in the *para* position relative to the hydroxy group and this new fragment 11a was docked into the GLO1 active site (Fig. 3b and c, S2 and Table S4†). Compound 11a fills the hydrophobic active site almost completely with the phenyl group possibly engaging in hydrophobic or π–π interactions with Phe62, Leu182, or Cys60. Consequently, it was expected that 11a should also show improved inhibitory activity compared to 11. To validate the computation predictions, 11a and six other *para* substituted derivatives of 11 were synthesized and tested for their inhibitory activity against GLO1 (Table 3). All compounds were obtained in just a few synthetic steps using well established methods (see ESI†). Aryl substituted derivatives 11a–11c and 11g were prepared by Suzuki cross-coupling from aryl boronic acids, while saturated N-heterocycle substituted derivatives 11d–11f by Buchwald–Hartwig coupling with cyclic amines. This highlights that salicylate MBIs are easy to synthesize and derivatize, making them ideal candidates for FBDD and lead development. It was found that aryl substituted derivatives 11a–11c showed significantly better inhibition of GLO1 compared to 11 (Table 3). The IC_50_ of 11 was 19.6 ± 4.7 μM whereas the IC_50_ of 11c was determined to be 3.3 ± 0.7 μM, which confirms the docking and virtual fragment growth results. Exchanging the phenyl ring for smaller aromatic moieties did not improve inhibitory activity. The IC_50_ of furane 11g was similar to phenyl derivatives 11a–11c. The inhibitory activity of piperidine 11d and morpholine 11e was significantly worse than 11. Interestingly, the derivative substituted with the smaller pyrrolidine 11f showed similarly good inhibition as the aryl substituted derivatives. This suggests that ring-size and shape has a larger effect on GLO1 inhibition for saturated N-heterocycles than for the aryl substituted derivatives of 11. The inhibitory activity of compounds 11a–c and 11f–g is comparable to the known micromolar inhibitor Myricetin,^36^ which gave an IC_50_ of 4.8 ± 1.3 μM under identical assay conditions (Fig. S13†).

**Table 3:** Inhibitory activity of 11 and derivatives against GLO1

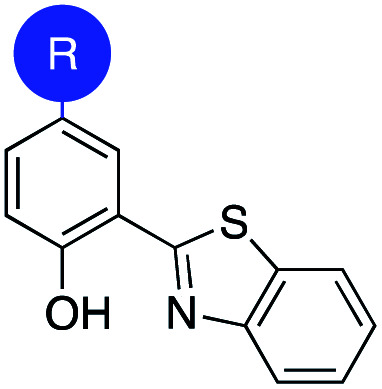		
Compound	R	IC_50_ [μM]
11	H	19.6 ± 4.7
11a	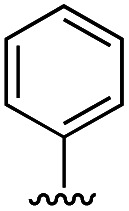	5.0 ± 1.5
11b	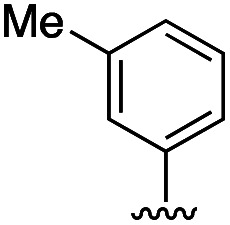	6.8 ± 1.4
11c	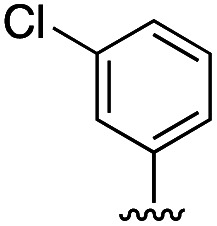	3.3 ± 0.7
11d	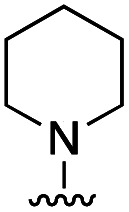	>20
11e	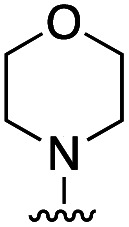	>50
11f	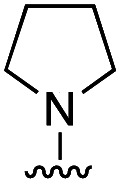	4.4 ± 1.8
11g	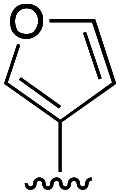	6.9 ± 2.8

## Conclusions

Salicylate MBIs are easy to synthesize and allowed for rapid preparation of 27 compounds, of which nearly half were wholly new compositions of matter. They show a variety of metal binding modes in a [(Tp^Ph,Me^)Zn(MBI)] model system. Indeed the majority (>20) of the MBIs generated had no previously described coordination chemistry. Unexpected binding modes were observed for salicylate MBIs capable of forming intramolecular hydrogen bonds, which will open new avenues in the development of metalloenzyme inhibitors. The physicochemical properties of salicylate MBIs are tuneable with a wide range of acidities and lipophilicities achieved. Importantly, the lipophilicities of most salicylate MBIs tested are in a desirable range for FBDD. The screening of their inhibitory activity against three metalloenzyme targets revealed that isosteric replacements in MBPs cannot only maintain, but potentially improve inhibitory activity and that salicylate MBIs can inhibit various metalloenzyme targets containing different metals. Supported by computational docking studies and rudimentary SAR, the enzyme binding of a selected salicylate MBI was investigated and the inhibitory activity improved. This demonstrates that salicylate MBIs are an attractive starting point for FBDD and lead development. In conclusion, salicylate MBIs are an interesting new class of metalloenzyme binding fragments with beneficial physicochemical properties and will be useful for the development of new metalloenzyme targeting inhibitors with more drug-like properties.

## Experimental

### General information

All solvents and reagents, unless otherwise noted, were obtained from commercial sources and used without further purification. MBIs 2, 4, 6, and 11 were purchased from Combi-Blocks Inc. and used without further purification. All reactions, unless otherwise stated, were performed under a nitrogen atmosphere. Silica chromatography was performed using a CombiFlash Rf Teledyne ISCO system using hexane, ethyl acetate, methylene chloride, or methanol as eluents. C18 reverse-phase chromatography was performed using the same instrument using 0.1% formic acid in methanol, acetonitrile, or water as eluent. Separations were monitored by mass spectrometry *via* a Teledyne ISCO RF^+^ PurIon ESI-MS detector with 1 Da resolution. ^1^H- and ^13^C-NMR spectra were obtained on a Varian (400 MHz) spectrometer or Jeol (500 MHz) spectrometer or a VX (500 MHz) equipped with an XSens cold probe (Varian) spectrometer in the Department of Chemistry and Biochemistry at U.C. San Diego. Standard resolution MS was performed either at U.C. San Diego Molecular Mass Spectrometry Facility or on the aforementioned Teledyne ISCO RF + PurIon MS. High resolution mass spectrometry (HRMS) analysis was performed using an Agilent 6230 accurate-mass liquid chromatography time-of-flight mass spectrometry LC-TOFMS located at the U.C. San Diego Molecular Mass Spectrometry Facility.

### General synthesis and characterization of model complexes

[(Tp^Ph,Me^)ZnOH] was prepared according to literature known procedures.^37^ [(Tp^Ph,Me^)ZnOH] (50 mg, 0.09 mmol) was dissolved in 15 mL of CH_2_Cl_2_ in a 50 mL round-bottom flask. The MBI (0.09 mmol, 1 equiv.) in 10 mL of MeOH was added, and the reaction mixture was stirred overnight under a nitrogen atmosphere. The resulting mixture was evaporated to dryness and subsequently dissolved in a minimal amount (∼1 mL) of benzene. The solution was filtered using a syringe filter to remove any undissolved solids. The resulting complex in benzene was recrystallized using vapor diffusion with pentane. Crystals typically formed within a few days.

### Physicochemical properties analysis

Physicochemical properties were determined using a Sirius T3 instrument.^23–25^ All titrations, both p*K*_a_ and log *P*, were performed in 0.15 M KCl with 0.5 M HCl and KOH. The p*K*_a_ of a compound was determined by analysing each MBI sample in triplicate using potentiometric titrations. Experiments were typically performed over a pH range of 2.0–12.0. Standard deviations were derived from fitting all three replicate experiments. For water insoluble compounds methanol was added and the obtained apparent p*K*_a_ (p_s_*K*_a_) was extrapolated to the aqueous p*K*_a_ by the Yasuda–Shedlovsky procedure. log *P* was determined *via* potentiometric titrations in the presence of varying ratios of octanol and water. The presence of octanol shifts the p*K*_a_ of ionizable species, and based on the shifts, a log *P* can be determined. Measurements for log *P* determination were typically performed over a pH range of 2.0–12.0. Three experiments with varying ratios of water:octanol was performed, allowing for a standard deviation to be determined from the fitting of all measurements. MBI sample sizes were ∼0.5 mg for both p*K*_a_ and log *P* measurements.

### GLO1 assay

Recombinant human glyoxalase I (GLO1) was purchased from R&D Systems (catalog #4959-GL). Assays were carried out in 0.1 M sodium phosphate, pH 7.0 buffer, utilizing 96-well clear UV plates (Corning UV Transparent Microplates, catalog #3635). A fresh solution of GSH (100 mM) and methylglyoxal (MG) (100 mM) was prepared in Millipore grade water. The substrate for the assay was prepared by adding 0.43 mL of GSH and 0.43 mL of MG to 15.14 mL of buffer. The substrate mixture was vortexed vigorously for 45 s and then allowed to sit at room temperature for 15 min. The initial well volume was 50 μL containing GLO1 (40 ng) and inhibitor. This protein and inhibitor mixture was incubated for 15–20 min prior to addition of the substrate. The substrate (150 μL) was then added to the wells yielding a maximum amount of 5% DMSO per well. The enzyme activity was measured utilizing a Biotek Synergy HT or H4 plate reader by measuring absorbance at 240 nm every 1 min for a duration of 8 min. The rate of absorbance increase was compared for samples *versus* controls containing no inhibitor (set at 100% activity). The absorbance reading for background wells containing DMSO, buffer, and substrate (no enzyme or inhibitor) was subtracted from the experimental wells. A positive control (1-hydroxy-4,6-diphenylpyridin-2(1*H*)-one, 50 μM)^33^ showed complete inhibition under the assay conditions described above. Dose-response curves were generated, analysed and fitted to obtain IC_50_ values (see ESI†). Myricetin,^36^ a known micromolar inhibitor, gave an IC_50_ value of 4.8 ± 1.3 μM under these assay conditions.

### MMP-3 assay

Human recombinant MMP-3 catalytic domain was purchased from ENZO Life Sciences (catalog # BML-SE109-9090). Assays were carried out in clear Costar 96-well, half-area, flat-bottom assay plates (catalog # 80-2404). Each well contained a total volume of 100 μL including buffer (50 mM MES, 10 mM CaCl_2_, 0.05% Brij-35, 1 mM DNTB, pH 6.0), MMP-3 (2 U), and the fragment solution. After a 30 min incubation period at 37 °C, the reaction was initiated by the addition of 10 μL of chromogenic MMP-3 substrate (100 μM final concentration, Ac-Pro-Leu-Gly-[2-mercap-to-4-methyl-pentanoyl]-Leu-Gly-OC_2_H_5_, ENZO Life Sciences, catalog #BML-P125-0005). Absorbance was monitored at 412 nm using a Bio-Tek ELx 808 colorimetric plate reader, and measurements were recorded every minute for 10 min. The rate of absorbance increase was compared for samples *versus* negative controls (no inhibitor, arbitrarily set as 100% activity). A positive control (NNGH, 1.3 μM) showed complete inhibition under the assay conditions described above.

### PA_N_ assay

Endonuclease was expressed and purified as previously reported.^38^ The activity assays were carried in black Costar 96-well plates with a total volume of 100 μL per well. Assay buffer consisted of 20 mM Tris pH 8.0, 150 mM NaCl, 2 mM MnCl_2_, 2 mM MgCl_2_, 10 mM 2-mercaptoethanol, and 0.2% Triton-X100. Endo was included at a final concentration of 25 nM, and MBIs were added from a 50 mM DMSO stock to a final concentration of 100 μM. A fluorescent ssDNA-oligo substrate [([6-FAM]AATCGCAGGC-AGCACTC[TAM]), FAM = 6-carboxyfluorescein, TAM = tetrame-thylrhodamine] was used to monitor enzymatic activity. After addition of substrate, fluorescence (*λ*_ex_ = 490 nm, *λ*_em_ = 520 nm) was monitored over 45 min at 39 s intervals at 37 °C. Negative control wells contained no inhibitor and were set to 100% activity. A reported pyridinone-based inhibitor was included as a positive control for inhibition.^38^ Percent inhibition was determined by comparing the activity of wells containing MBIs to the activity of those without.

## Author contributions

M. K. J. and S. M. C. outlined the project, interpreted results, and wrote the manuscript. M. K. J. and S. M. C. designed the MBI library, M. K. J. planned and executed the synthesis of all compounds. M. K. J. and H. S. performed the enzyme activity assays and interpreted the results. H. S., M. K., and M. K. J. performed X-ray crystallographic measurements and edited the data. J. K. performed computational docking studies.

## Conflicts of interest

S.M.C. is a cofounder of and has an equity interest in Cleave Therapeutics, Forge Therapeutics, and Blacksmith Medicines, companies that may potentially benefit from the research results. S.M.C. also serves on the Scientific Advisory Board for Blacksmith Medicines and serves on the Scientific Advisory Board and receives compensation from Forge Therapeutics. The terms of this arrangement have been reviewed and approved by the University of California, San Diego in accordance with its conflict-of-interest policies.

## Supplementary Material

SC-013-D1SC06011B-s001

SC-013-D1SC06011B-s002

SC-013-D1SC06011B-s003

SC-013-D1SC06011B-s004
