# Supplementary material for: Salicylate metal-binding isosteres as fragments for metalloenzyme inhibition
Source: Chem Sci. 2022 Jan 21;13(7):2128–36. doi: 10.1039/d1sc06011b (PMC8849047; doi:10.1039/d1sc06011b)

Electronic Supplementary Material (ESI) for Chemical Science.
This journal is © The Royal Society of Chemistry 2022

Salicylate Metal-Binding Isosteres as Fragments for Metalloenzyme Inhibition

SUPPORTING INFORMATION

Moritz K. Jackl, Hyeonglim Seo, Johannes Karges, Mark Kalaj, and Seth M. Cohen*

Department of Chemistry and Biochemistry, University of California, San Diego

9500 Gilman Drive, La Jolla, CA 92093-0358, USA

E-mail: scohen@ucsd.edu

Table of Contents

1. General information 3

2. Preparation of Salicylate MBIs 4

3. Preparation of MBI 11 derivatives 28

4. Computational docking 34

5. Single Point screening 38

6. Dose-response curves against GLO1 40

7. X-Ray crystallography data 44

8. Calculated physicochemical properties 49

9. References 51

10. NMR Spectra 53

1. General information

All solvents and reagents, unless otherwise noted, were obtained from commercial sources and used without further purification. Compounds **2**, **4**, **6**, and **11** were purchased from Combi-Blocks Inc. and used without further purification. All reactions, unless otherwise stated, were performed under a nitrogen atmosphere. Silica chromatography was performed using a CombiFlash Rf Teledyne ISCO system using hexane, ethyl acetate, methylene chloride, or methanol as eluents. C18 reverse-phase chromatography was performed using the same instrument using 0.1% formic acid in methanol, acetonitrile, or water as eluent. Separations were monitored by mass spectrometry via a Teledyne ISCO RF^+ PurIon ESI-MS detector with 1 Da resolution. ^1H- and ^{13}C-NMR spectra were obtained on a Varian (400 MHz) spectrometer or Jeol (500 MHz) spectrometer or a VX (500 MHz) equipped with an XSens cold probe (Varian) spectrometer in the Department of Chemistry and Biochemistry at U.C. San Diego. Standard resolution mass spectrometry (MS) was performed either at U.C. San Diego Molecular Mass Spectrometry Facility or on the aforementioned Teledyne ISCO RF^+ PurIon MS. High resolution mass spectrometry (HRMS) analysis was performed using an Agilent 6230 accurate-mass liquid chromatography time-of-flight mass spectrometry LC−TOFMS located at the U.C. San Diego Molecular Mass Spectrometry Facility.

2. Preparation of Salicylate MBIs

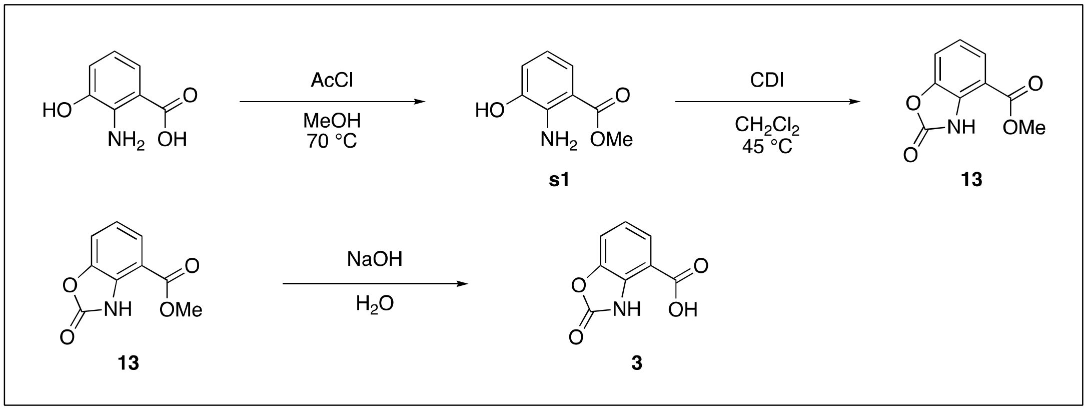

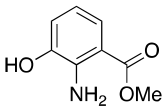

Methyl 2-amino-3-hydroxybenzoate (s1)

2-Amino-3-hydroxybenzoic acid (1.00 g, 7.00 mmol, 1.00 equiv) was dissolved in MeOH (15 mL) and acetyl chloride (0.90 mL, 14.0 mmol, 2.00 equiv) was added dropwise. The resulting dark-brown solution was refluxed for 14 h. All volatiles were removed under reduced pressure and the resulting solid was suspended in H_2O (5 mL). Sat. $NaHCO_3$ was added until a basic pH was reached, EtOAc (100 mL) was added and the phases were separated. The aqueous phase was extracted with EtOAc (3x50 mL) and the combined organics were washed with brine (50 mL), dried and concentrated under reduced pressure to obtain the desired product, which was used for the next steps without any further purifications. **^{1}H NMR** (400 MHz, $CDCl_3$): δ [ppm] = 7.53 – 7.43 (m, 1 H), 6.86 – 6.77 (m, 1H), 6.56 – 6.44 (m, 1H), 5.53 (br s, 2H), 3.87 (s, 3H); **^{13}C NMR** (100 MHz, $CDCl_3$): δ [ppm] = 169.2, 143.7, 140.9, 123.5, 118.3, 115.6, 118.8, 52.0. These spectral characteristics were identical to those previously reported.[1]

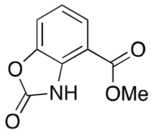

Methyl 2-oxo-2,3-dihydrobenzo[*d*]oxazole-4-carboxylate (13)

Compound **s1** (200 mg, 1.20 mmol, 1.00 equiv) was dissolved in CH_2Cl_2 (4 mL) and CDI (388 mg, 2.40 mmol, 2.00 equiv) was added as a solid. The resulting mixture was stirred for 14 h at 45 °C. Water (10 mL) was added and the mixture was extracted with EtOAc (3x20 mL). The combined organics were washed with 1 M HCl (3x5 mL) and brine (10 mL), dried over $MgSO_4$, and concentrated under reduced pressure. Purification by column chromatography (hexanes:EtOAc 10:1 to 1:1) to obtain the desired compound as an off-white solid. Yield: 170 mg (74%). **^{1}H NMR** (400 MHz, DMSO-d_6): *δ* [ppm] = 11.82 (br s, 1H), 7.67 – 7.60 (m, 1H), 7.58 – 7.51 (m, 1H), 7.22 – 7.13 (m, 1H), 3.88 (s, 3H); **^{13}C NMR** (100 MHz, DMSO-d_6): δ [ppm] = 164.5, 154.4, 143.9, 131.3, 124.3, 121.6, 113.9, 112.4, 52.2; **HRMS** (ESI): calculated for $[C_9H_8NO_4]^+$: *m/z* = 194.0448, found: *m/z* = 194.0449.

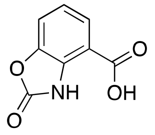

2-Oxo-2,3-dihydrobenzo[*d*]oxazole-4-carboxylic acid (3)

Compound **13** (80.0 mg, 0.41 mmol, 1.00 equiv) was suspended in 1 M NaOH (3 mL) and stirred for 3 h at room temperature. 1 M HCl was added until a pH <1 was reached and the mixture was extracted with EtOAc (3x10 mL). The combined organics were washed with brine (10 mL), dried over $MgSO_4$, and concentrated under reduced pressure. Purification by C18 column chromatography (MeOH:H_2O 1:10 to 10:1 + 0.1% HCO_2H) was used to obtain the desired product as a colorless solid. Yield: 50 mg (67%). **^{1}H NMR** (400 MHz, DMSO-d_6): *δ* [ppm] = 13.42 (br s, 1H), 11.68 (br s, 1H), 7.62 (dd, J = 8.1, 1.1 Hz, 1H), 7.51 (dd, J = 8.0, 1.1 Hz, 1H), 7.16 (t, J = 8,1 Hz, 1H); **^{13}C NMR** (100 MHz, DMSO-d_6): *δ* [ppm] = 165.7, 154.3,

143.9, 131.5, 124.5, 121.4, 113.7, 113.5; **HRMS** (ESI): calculated for $[C_8H_4NO_4]^-$: *m/z* = 178.0146, found: *m/z* = 178.0146.

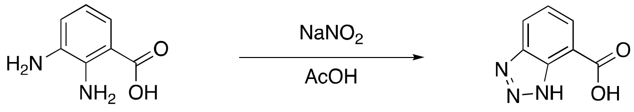

5

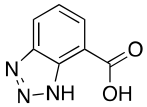

1*H*-Benzo[*d*][1,2,3]triazole-7-carboxylic acid (5)

2,3-Diaminobenzoic acid (300 mg, 1.97 mmol, 1.00 equiv) was dissolved in H_2O (2 mL) and AcOH (1 mL) and stirred for 5 min. Afterwards, a solution of $NaNO_2$ (151 mg, 2.19 mmol, 1.11 equiv) in H_2O (2 mL) was added dropwise. The resulting suspension was stirred for 18 h at room temperature. The brown solid was filtered off, washed with H_2O (3x20 mL) and dried under high-vacuum. Purification by C18 column chromatography (MeOH:H_2O 1:10 to 10:1 + 0.1% HCO_2H) produced the desired product as an off-white solid. Yield: 190 mg (60%). **^{1}H NMR** (400 MHz, DMSO-d_6): δ [ppm] = 13.71 (br s, 1H), 8.34 (dd, J = 8.3, 1.0 Hz, 1H), 8.10 (d, J = 7.3 Hz, 1H), 7.61 – 7.41 (m, 1H); **^{13}C NMR** (100 MHz, DMSO-d_6): δ [ppm] = 166.3, 145.8, 132.2, 129.9, 124.5, 124.0, 116.0; **HRMS** (ESI): calculated for $[C_7H_4N_3O_2]^-$: *m/z* = 162.0309, found: *m/z* = 162.0310.

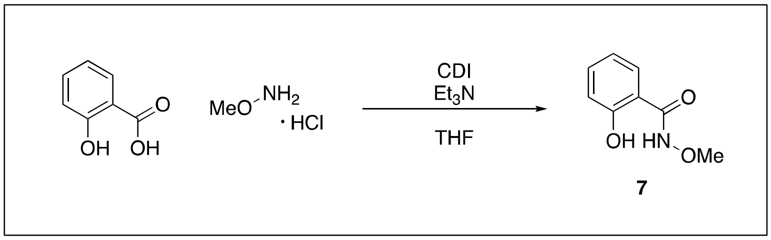

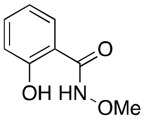

2-Hydroxy-*N*-methoxybenzamide (7)

Salicylic acid (150 mg, 1.09 mmol, 1.00 equiv) was suspended in anhydrous THF (3 mL). CDI (264 mg, 1.63 mmol, 1.50 equiv) was added as a solid and the resulting mixture was stirred for 0.5 h at room temperature. *O*-Methylhydroxylamine hydrochloride (181 mg, 2.17 mmol, 2.00 equiv) and Et_3N (0.30 mL, 2.17 mmol, 2.00 equiv) were added and the resulting suspension was stirred at room temperature for 14 h. All volatiles were removed and the resulting brownish oil was purified by column chromatography (hexanes:EtOAc 10:1 to 1:1) to obtain the desired product as a colorless solid. Yield: 100 mg (55%). **^{1}H NMR** (400 MHz, $CDCl_3$): δ [ppm] = 11.5 (br s, 1H), 9.20 (br s, 1H), 7.46 – 7.20 (m, 2H), 7.06 – 6.94 (m, 1H), 6.90 – 6.77 (m, 1H), 3.89 (s, 3H); **^{13}C NMR** (100 MHz, $CDCl_3$): δ [ppm] = 169.1, 161.3, 135.0, 125.5, 119.2, 188.8, 112.2, 65.0; **HRMS** (ESI): calculated for $[C_8H_{10}NO_3]^+$: m/z = 168.0655, found: m/z = 168.0657.

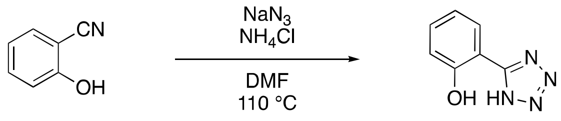

8

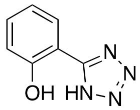

2-(1*H*-Tetrazol-5-yl)phenol (8)

2-Hydroxybenzonitrile (240 mg, 2.01 mmol, 1.00 equiv), NaN_3 (262 mg, 4.03 mmol, 2.00 equiv) and NH_4Cl (216 mg, 4.03 mmol, 2.00 equiv) were suspended in anhydrous DMF (3 mL) and the resulting mixture was stirred at 110 °C for 2 h (progress of the reaction monitored by TLC). H_2O (15 mL) was added to obtain a clear solution. 6 M HCl was added dropwise until a slightly acidic pH was reached and a colorless solid crushed out. The colorless solid was collected by filtration, washed with H_2O (3x30 mL) and dried under high-vacuum to obtain the desired product as a colorless solid. Yield: 150 mg (50%). **^{1}H NMR** (400 MHz, DMSO-d_6): δ [ppm] = 10.99 (br s, 1H), 7.99 (dd, J = 7.8, 1.7 Hz, 1H), 7.43 – 7.39 (m, 1H), 7.07 (dd, J = 8.3, 1.1 Hz, 1H), 7.00 (td, J = 7.5, 1.1 Hz, 1H); **^{13}C NMR** (100 MHz, DMSO-d_6): δ [ppm] = 155.4, 151.7, 132.2, 129.1, 120.2, 116.4, 111.0; **HRMS** (ESI): calculated for $[C_7H_5N_4O]^-$: *m/z* = 161.0469, found: *m/z* = 161.0470.

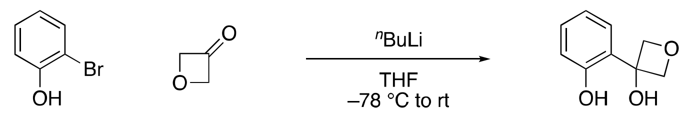

9

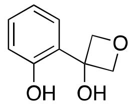

3-(2-Hydroxyphenyl)oxetan-3-ol (9)

2-Bromophenol (242 mg, 1.40 mmol, 1.00 equiv) was dissolved in anhydrous THF (4 mL) and ^{n}BuLi (1.6 M in hexanes, 3.08 mmol, 2.2 equiv) was added dropwise at –78 °C. The mixture was allowed to warm to room temperature and stirred for 30 min. The suspension was cooled back to –78 °C and oxetane-3-one (121 mg, 1.68 mmol, 1.20 equiv) in THF (2 mL) was added dropwise. The resulting mixture was allowed to warm to room temperature and stirred for 14 h. Sat. NH_4Cl (10 mL) was added and the mixture was extracted with EtOAc (3x20 mL). The combined organics were washed with brine, dried over $MgSO_4$ and concentrated under reduced pressure. Purification by column chromatography (hexanes:EtOAc 10:1 to 1:1) to obtain the desired product as a colorless solid. Yield: 50 mg (22%). **^{1}H NMR** (400 MHz, $CDCl_3$): δ [ppm] = 7.34 (dd, J = 7.7, 1.6 Hz, 1H), 7.26 – 7.21 (m, 1H), 6.96 (td, J = 7.6, 1.2 Hz, 1H), 6.87 (dd, J = 8.1, 1.2 Hz, 1H), 5.06 (dd, J = 7.2, 1.0 Hz, 2H), 4.91 (dd, J = 7.2, 1.0 Hz, 2H); **^{13}C NMR** (100 MHz, $CDCl_3$): δ [ppm] = 154.6, 130.1, 126.0, 125.3, 120.7, 117.3, 84.2, 77.3; **HRMS** (ESI): calculated for $[C_9H_9O_3]^-$: m/z = 165.0557, found: m/z = 165.0557.

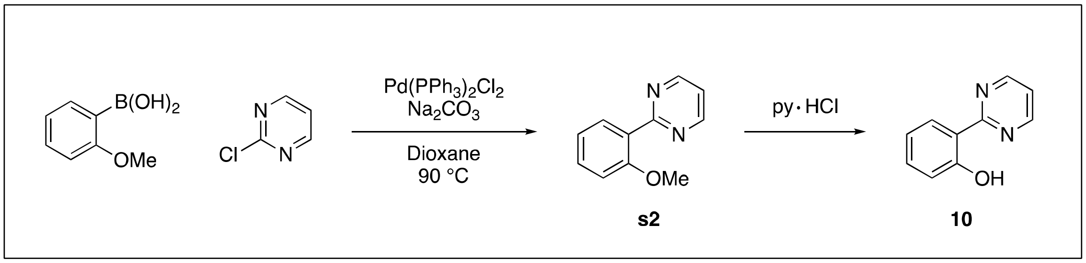

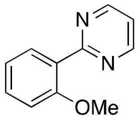

2-(2-Methoxyphenyl)pyrimidine (s2)

The compound was prepared according to a modified procedure from the literature.[1] (2-Methoxyphenyl)boronic acid (182 mg, 1.20 mmol, 1.20 equiv), 2-chloro-pyrimidine (115 mg, 1.00 mmol, 1.00 equiv), and $Pd(PPh_3)_2Cl_2$ were suspended in dioxane (2.5 mL). Afterwards, 2 M Na_2CO_3 (2.5 mL) was added and the resulting mixture was stirred at 90 °C for 5 h (progress of the reaction was monitored by TLC). H_2O (5 mL) and EtOAc (15 mL) were added, the phases were separated and the aqueous phase was extracted with EtOAc (3x10 mL). The combined organic phases were washed with brine, dried over $MgSO_4$, and concentrated under reduced pressure. Purification by column chromatography (hexanes:EtOAc 10:1 to 1:1) to obtain the desired product as a colourless solid. Yield: 140 mg (75%). **^{1}H NMR** (400 MHz, $CDCl_3$): δ [ppm] = 8.86 (d, J = 4.9 Hz, 2H), 7.77 – 7.66 (m, 1H), 7.48 – 7.37 (m, 1H), 7.22 (t, J = 4.9 Hz, 1H), 7.12 – 6.97 (m, 2H), 3.87 (s, 3H). These spectral characteristics matched those previously reported in the literature.[2]

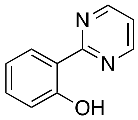

2-(Pyrimidin-2-yl)phenol (10)

The compound was prepared according to a modified procedure from the literature.[1] Compound **s2** (140 mg, 0.75 mmol, 1.00 equiv) and pyridine hydrochloride (350 mg, 3.00 mmol, 4.00 equiv) were heated to 210 °C and the resulting solution was stirred for 1 h. After

cooling to room temperature, EtOAc (20 mL) and sat. $NaHCO_3$ (10 mL) were added the phases were separated and the aqueous phase was extracted with EtOAc (3x10 mL). The combined organic phases were washed with brine, dried over $MgSO_4$, and concentrated under reduced pressure. Purification by column chromatography (hexanes:EtOAc 10:1 to 1:1) to obtain the desired product as a colourless solid. Yield: 60 mg (47%). **^{1}H NMR** (400 MHz, $CDCl_3$): δ [ppm] = 8.79 (d, J = 4.9 Hz, 2H), 8.52 – 8.45 (m, 1H), 7.43 – 7.36 (m, 1H), 7.23 (t, J = 4.9 Hz, 1H), 7.06 – 7.00 (m, 1H), 7.00 – 6.90 (m, 1H); **^{13}C NMR** (100 MHz, $CDCl_3$): δ [ppm] = 165.4, 160.7, 156.2, 133.5, 129.3, 119.3, 118.8, 118.4, 118.1. These spectral characteristics matched those previously reported in the literature.[2]

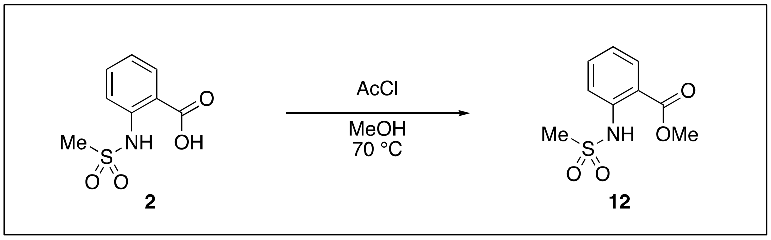

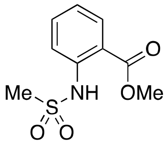

Methyl 2-(methylsulfonamido)benzoate (12)

Compound **2** (150 mg, 0.70 mmol, 1.00 equiv) was dissolved in anhydrous MeOH (2 mL) and acetyl chloride (0.15 mL, 2.10 mmol, 3.00 equiv) was added dropwise. The mixture was stirred at 70 °C for 14 h. All volatiles were removed under reduced pressure and sat. $NaHCO_3$ was added until a neutral pH was reached. The aqueous phase was extracted with EtOAc (3x10 mL). The combined organics were washed with brine (10 mL), dried over $MgSO_4$ and concentrated under reduced pressure. Purification by column chromatography (hexanes:EtOAc 10:1 to 3:1) produced the desired product as a colorless solid. Yield: 30 mg (20%). **^{1}H NMR** (400 MHz, $CDCl_3$): δ [ppm] = 10.48 (br s, 1H), 8.10 – 8.00 (m, 1H), 7.80 – 7.68 (m, 1H), 7.60 – 7.50 (m, 1H), 7.18 – 7.06 (m, 1H), 3.94 (s, 3H), 3.06 (s, 3H); **^{13}C NMR**

(100 MHz, $CDCl_3$): δ [ppm] = 168.5, 140.9, 135.1, 131.7, 123.0, 118.1, 115.4, 52.8, 40.1; **HRMS** (ESI): calculated for $[C_9H_{11}NO_4SNa]^+$: m/z = 252.0301, found: m/z = 252.0304.

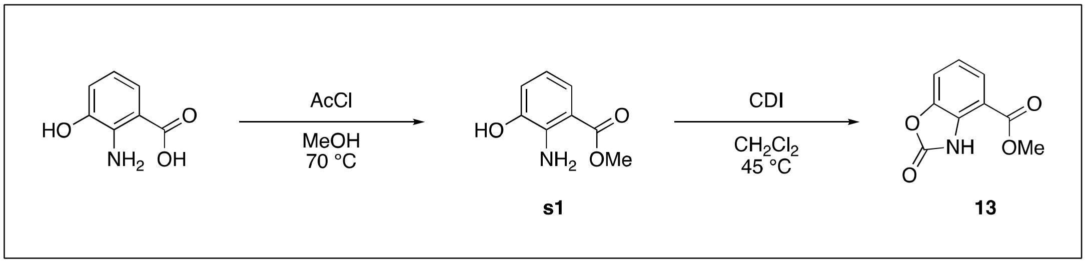

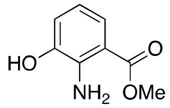

Methyl 2-amino-3-hydroxybenzoate (s1)

2-Amino-3-hydroxybenzoic acid (1.00 g, 7.00 mmol, 1.00 equiv) was dissolved in MeOH (15 mL) and acetyl chloride (0.90 mL, 14.0 mmol, 2.00 equiv) was added dropwise. The resulting dark-brown solution was refluxed for 14 h. All volatiles were removed under reduced pressure and the resulting solid was suspended in H_2O (5 mL). Sat. $NaHCO_3$ was added until a basic pH was reached, EtOAc (100 mL) was added and the phases were separated. The aqueous phase was extracted with EtOAc (3x50 mL) and the combined organics were washed with brine (50 mL), dried and concentrated under reduced pressure to obtain the desired product, which was used for the next steps without any further purifications. **^{1}H NMR** (400 MHz, $CDCl_3$): δ [ppm] = 7.53 – 7.43 (m, 1 H), 6.86 – 6.77 (m, 1H), 6.56 – 6.44 (m, 1H), 5.53 (br s, 2H), 3.87 (s, 3H); **^{13}C NMR** (100 MHz, $CDCl_3$): δ [ppm] = 169.2, 143.7, 140.9, 123.5, 118.3, 115.6, 118.8, 52.0. These spectral characteristics were identical to those previously reported.[1]

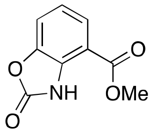

Methyl 2-oxo-2,3-dihydrobenzo[*d*]oxazole-4-carboxylate (13)

Methyl 2-amino-3-hydroxybenzoate (200 mg, 1.20 mmol, 1.00 equiv) was dissolved in CH_2Cl_2 (4 mL) and CDI (388 mg, 2.40 mmol, 2.00 equiv) was added as a solid. The resulting mixture was stirred for 14 h at 45 °C. Water (10 mL) was added and the mixture was extracted with EtOAc (3x20 mL). The combined organics were washed with 1 M HCl (3x5 mL) and brine (10 mL), dried over $MgSO_4$, and concentrated under reduced pressure. Purification by column chromatography (hexanes:EtOAc 10:1 to 1:1) to obtain the desired compound as an off-white solid. Yield: 170 mg (74%). **^{1}H NMR** (400 MHz, DMSO-d_6): *δ* [ppm] = 11.82 (br s, 1H), 7.67 – 7.60 (m, 1H), 7.58 – 7.51 (m, 1H), 7.22 – 7.13 (m, 1H), 3.88 (s, 3H); **^{13}C NMR** (100 MHz, DMSO-d_6): δ [ppm] = 164.5, 154.4, 143.9, 131.3, 124.3, 121.6, 113.9, 112.4, 52.2; **HRMS** (ESI): calculated for $[C_9H_8NO_4]^+$: *m/z* = 194.0448, found: *m/z* = 194.0449.

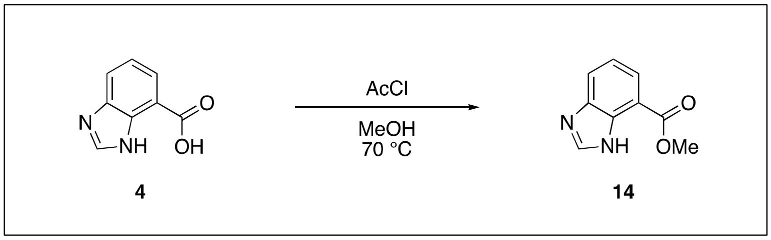

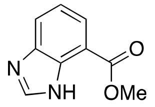

Methyl 1*H*-benzo[*d*]imidazole-7-carboxylate (14)

Compound **4** (200 mg, 1.23 mmol, 1.00 equiv) was dissolved in anhydrous MeOH (2.5 mL) and acetyl chloride (0.26 mL, 3.70 mmol, 3.00 equiv) was added dropwise. The mixture was stirred at 70 °C for 14 h. All volatiles were removed under reduced pressure and sat. $NaHCO_3$ was added until a neutral pH was reached. The aqueous phase was extracted with EtOAc (3x10 mL). The combined organics were washed with brine (10 mL), dried over $MgSO_4$, and

concentrated under reduced pressure. Purification by column chromatography (hexanes:EtOAc 10:1 to 1:1) to obtain the desired product as a yellow solid. Yield: 120 mg (55%). **^{1}H NMR** (400 MHz, $CDCl_3$): *δ* [ppm] = 8.17 (s, 1H), 8.04 (d, J = 8.1 Hz, 1H), 7.95 (d, J = 7.7 Hz, 1H), 7.37 – 7.30 (m, 1H), 4.00 (s, 3H); **^{13}C NMR** (100 MHz, $CDCl_3$): *δ* [ppm] = 167.0, 143.7, 141.6, 133.4, 125.6, 125.4, 122.0, 113.9, 52.4; **HRMS** (ESI): calculated for $[C_9H_9N_2O_2]^+$: *m/z* = 177.0659, found: *m/z* = 177.0660.

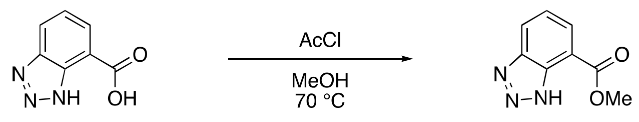

5 15

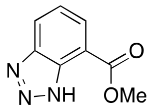

Methyl 1*H*-benzo[*d*][1,2,3]triazole-7-carboxylate (15)

Compound **5** (70.0 mg, 0.43 mmol, 1.00 equiv) was dissolved in anhydrous MeOH (1.00 mL) and acetyl chloride (0.09 mL, 1.30 mmol, 3.00 equiv) was added dropwise. The mixture was stirred at 70 °C for 14 h. All volatiles were removed under reduced pressure and sat. $NaHCO_3$ was added until a neutral pH was reached. The aqueous phase was extracted with EtOAc (3x10 mL). The combined organics were washed with brine (10 mL), dried over $MgSO_4$ and concentrated under reduced pressure. Purification by column chromatography (hexanes:EtOAc 10:1 to 1:1) to obtain the desired product as a yellow solid. Yield: 30 mg (40%). **^{1}H NMR** (400 MHz, $CDCl_3$): *δ* [ppm] = 8.41 – 8.23 (m, 1H), 8.23 – 8.15 (m, 1H), 7.54 – 7.44 (m, 1H), 4.10 (s, 3H); **^{13}C NMR** (100 MHz, $CDCl_3$): *δ* [ppm] = 166.0, 145.5, 132.4, 130.1, 125.4, 124.0, 114.0, 53.0; **HRMS** (ESI): calculated for $[C_8H_8N_3O_2]^+$: *m/z* = 178.0611, found: *m/z* = 178.0612.

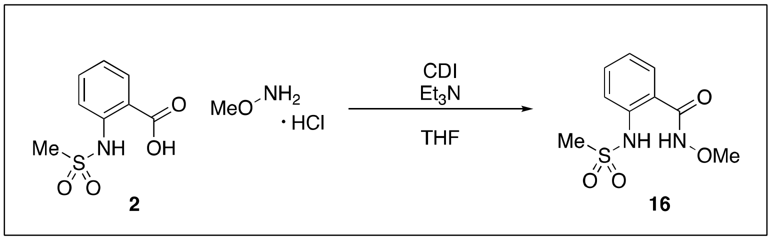

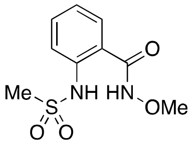

***N*-Methoxy-2-(methylsulfonamido)benzamide (16)**

Compound **2** (220 mg, 1.02 mmol, 1.00 equiv) was suspended in anhydrous THF (3 mL). CDI (249 mg, 1.53 mmol, 1.50 equiv) was added as a solid and the resulting mixture was stirred for 0.5 h at room temperature. *O*-methylhydroxylamine hydrochloride (142 mg, 2.04 mmol, 2.00 equiv) and Et_3N (0.30 mL, 2.04 mmol, 2.00 equiv) were added and the resulting suspension was stirred at room temperature for 14 h. All volatiles were removed and the resulting brownish oil was purified by column chromatography (hexanes:EtOAc 10:1 to 1:10) to obtain the desired product as a colorless solid. Yield: 120 mg (50%). **^{1}H NMR** (400 MHz, CD_3OD): δ [ppm] = 7.70 – 7.65 (m, 1 H), 7.63 – 7.59 (m, 1H), 7.58 – 7.52 (m, 1H), 7.23 – 7.16 (m, 1H), 3.82 (s, 3H), 3.03 (s, 3H); **^{13}C NMR** (100 MHz, CD_3OD): δ [ppm] =167.8, 139.8, 134.1, 129.2, 125.0, 122.0, 121.1 64.5, 39.8; **HRMS** (ESI): calculated for $[C_9H_{12}N_2O_4SNa]^+$: *m/z* = 267.0410, found: *m/z* = 267.0408.

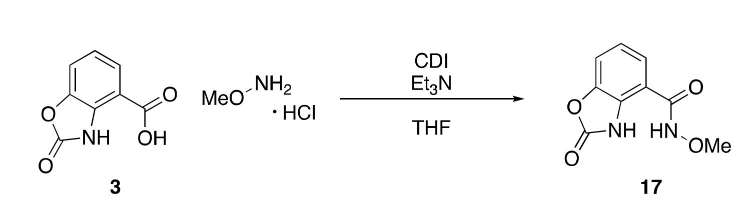

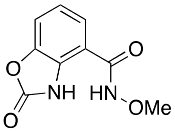

N-Methoxy-2-oxo-2,3-dihydrobenzo[*d*]oxazole-4-carboxamide (17)

Compound **3** (150 mg, 0.84 mmol, 1.00 equiv) was suspended in anhydrous THF (3 mL). CDI (163 mg, 1.00 mmol, 1.20 equiv) was added as a solid and the resulting mixture was stirred for 0.5 h at room temperature. *O*-methylhydroxylamine hydrochloride (116 mg, 1.68 mmol, 2.00 equiv) and Et_3N (0.23 mL, 1.68 mmol, 2.00 equiv) were added and the resulting suspension was stirred at room temperature for 14 h. All volatiles were removed and the resulting brownish oil was purified by column chromatography (CH_2Cl_2:MeOH 30:1 to 10:1) to obtain the desired product as a yellow solid. Yield: 60 mg (34%). **^{1}H NMR** (400 MHz, CD_3OD): δ [ppm] = 7.59 – 7.43 (m, 1H), 7.16 – 7.00 (m, 2H), 3.98 (s, 3H); **^{13}C NMR** (100 MHz, CD_3OD): δ [ppm] = 161.3, 149.9, 146.0, 129.4, 124.5, 120.0, 118.4, 116.7, 64.4; **HRMS** (ESI): calculated for $[C_9H_9N_2O_4]^+$: *m/z* = 209.0557, found: *m/z* = 209.0559.

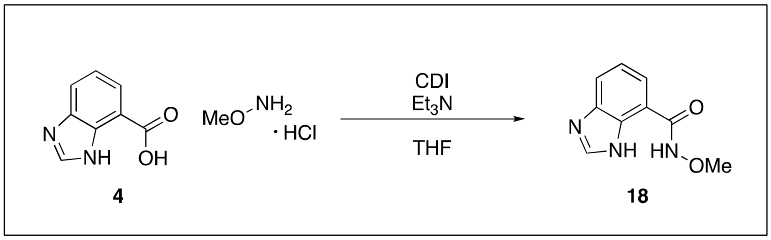

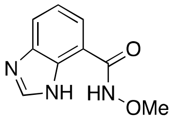

N-methoxy-1H-benzo[d]imidazole-7-carboxamide (18)

Compound **4** (170 mg, 1.05 mmol, 1.00 equiv) was suspended in anhydrous THF (3 mL). CDI (255 mg, 1.57 mmol, 1.50 equiv) was added as a solid and the resulting mixture was stirred for 0.5 h at room temperature. *O*-methylhydroxylamine hydrochloride (146 mg, 2.10 mmol, 2.00 equiv) and Et_3N (0.29 mL, 2.10 mmol, 2.10 equiv) were added and the resulting suspension was stirred at room temperature for 14 h. All volatiles were removed and the resulting brownish oil was purified by column chromatography (CH_2Cl_2:MeOH 30:1 to 10:1) to obtain the desired product as an orange solid. Yield: 110 mg (55%). **^{1}H NMR** (400 MHz, Acetone-d$_6$): δ [ppm] = 12.35 (br s, 1H), 8.43 (s, 1H), 8.01 (d, J = 7.6 Hz, 1H), 7.88 – 7.77 (m, 1H), 7.41 (t, J = 7.8 Hz, 1H), 3.88 (s, 3H); **^{13}C NMR** (100 MHz, Acetone-d$_6$): δ [ppm] = 163.9, 143.1, 140.9, 134.3, 123.7, 123.6, 122.3, 116.4, 64.4; **HRMS** (ESI): calculated for $[C_9H_{10}N_3O_2]^+$: *m/z* = 192.0768, found: *m/z* = 192.0767.

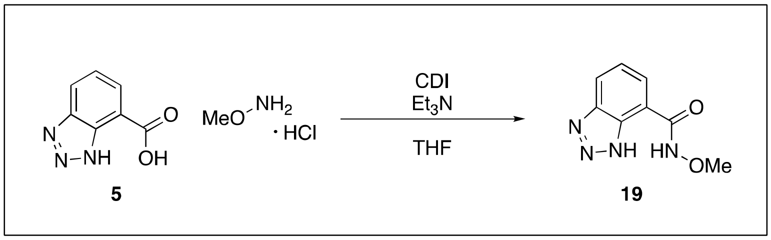

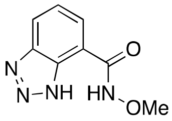

N-methoxy-1H-benzo[d][1,2,3]triazole-7-carboxamide (19)

Compound **5** (180 mg, 1.10 mmol, 1.00 equiv) was suspended in anhydrous THF (3 mL). CDI (268 mg, 1.65 mmol, 1.50 equiv) was added as a solid and the resulting mixture was stirred for 0.5 h at room temperature. *O*-methylhydroxylamine hydrochloride (153 mg, 2.21 mmol, 2.00 equiv) and Et_3N (0.31 mL, 2.21 mmol, 2.00 equiv) were added and the resulting suspension was stirred at room temperature for 14 h. All volatiles were removed and the resulting brownish oil was purified by column chromatography (CH_2Cl_2:MeOH 30:1 to 10:1) to obtain the desired product as a yellow solid. Yield: 125 mg (60%). **^{1}H NMR** (400 MHz, Acetone-d_6): δ [ppm] = 8.15 – 8.13 (m, 1H), 8.07 – 8.05 (m, 1H), 7.62 – 7.58 (m, 1H), 3.91 (s, 3H); **^{13}C NMR** (100 MHz, Acetone-d_6): δ [ppm] = 162.4, 140.2, 137.8, 126.4, 126.1, 119.9, 119.5, 64.3, **HRMS** (ESI): calculated for $[C_8H_8N_4O_2Na]^+$: m/z = 215.0539, found: m/z = 215.0540.

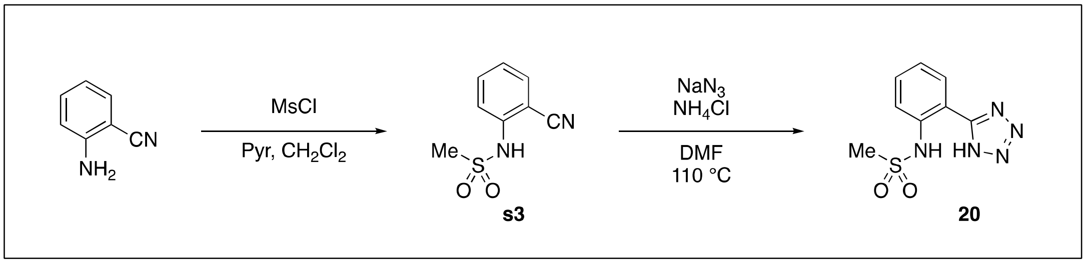

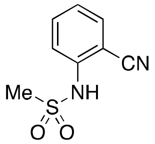

***N*-(2-cyanophenyl)methanesulfonamide (s3)**

2-aminobenzonitrile (300 mg, 2.50 mmol, 1.00 equiv) was dissolved in anhydrous CH_2Cl_2 (5 mL) and pyridine (0.30 mL, 3.80 mmol, 1.50 equiv) was added. The resulting solution was cooled to –78 °C and MsCl (0.21 mL, 2.80 mmol, 1.10 equiv) was added dropwise. The mixture was allowed to warm to room temperature and stirred for 14 h. H_2O (10 mL) and CH_2Cl_2 were added, the phases separated and the aqueous phase extracted with CH_2Cl_2 (3x20 mL). The combined organics were washed with brine (10 mL), dried over $MgSO_4$ and concentrated under reduced pressure. Purification by column chromatography (hexanes:EtOAc 10:1 to 3:1) to obtain the desired product, which was directly used for the next step. **^{1}H NMR** (400 MHz, $CDCl_3$): δ [ppm] = 7.77 – 7.69 (m, 1H), 7.68 – 7.59 (m, 2H), 7.34 – 7.20 (m, 1H), 7.00 (br s, 1H), 3.13 (s, 3H); These spectral characteristics were identical to those previously reported.[3]

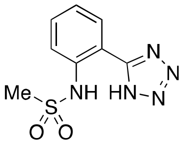

***N*-(2-(1*H*-tetrazol-5-yl)phenyl)methanesulfonamide (20)**

Compound **s3** (200 mg, 1.02 mmol, 1.00 equiv), NaN_3 (133 mg, 2.04 mmol, 2.00 equiv) and NH_4Cl (109 mg, 2.04 mmol, 2.00 equiv) were suspended in anhydrous DMF (3 mL) and the resulting mixture was stirred at 110 °C for 2 h (progress of the reaction monitored by TLC).

H_2O (15 mL) was added to obtain a clear solution. 6 M HCl was added dropwise until a slightly acidic pH was reached and a colorless solid crushed out. The colorless solid was collected by filtration, washed with H_2O (3x30 mL) and dried under high-vacuum to obtain the desired product as a colorless solid. Yield: 180 mg (74%). **^{1}H NMR** (400 MHz, DMSO-d$_6$): *δ* [ppm] = 10.25 (br s, 1H), 7.98 – 7.95 (m, 1H), 7.70 – 7.65 (m, 1H), 7.64 – 7.60 (m, 1H), 7.38 – 7.35 (m, 1H), 3.16 (s, 3H); **^{13}C NMR** (100 MHz, DMSO-d$_6$): *δ* [ppm] = 154.3, 136.6, 132.4, 129.3, 124.4, 120.5, 113.9; **HRMS** (ESI): calculated for $[C_8H_{10}N_5O_2S]^+$: *m/z* = 240.0550, found: *m/z* = 240.0551.

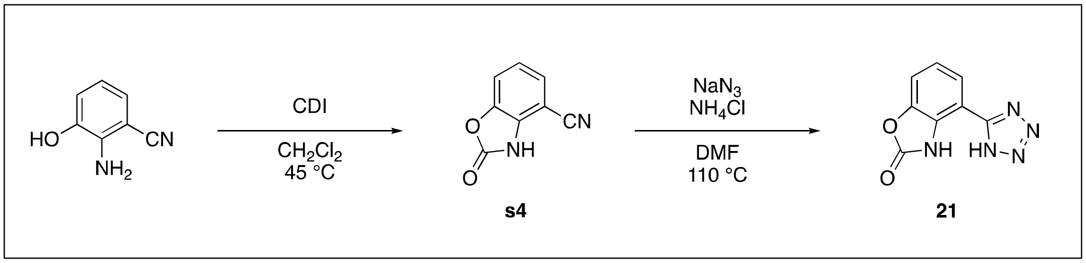

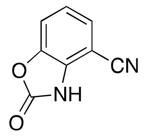

2-oxo-2,3-dihydrobenzo[*d*]oxazole-4-carbonitrile (s4)

2-amino-3-hydroxybenzonitrile (250 mg, 1.86 mmol, 1.00 equiv) was dissolved in CH_2Cl_2 (4 mL) and CDI (604 mg, 3.73 mmol, 2.00 equiv) was added as a solid. The resulting mixture was stirred for 14 h at 45 °C. Water (10 mL) was added and the mixture was extracted with EtOAc (3x20 mL). The combined organics were washed with 1 M HCl (3x5 mL) and brine (10 mL), dried over $MgSO_4$ and concentrated under reduced pressure. Purification by column chromatography (hexanes:EtOAc 10:1 to 1:1) to obtain the desired product, which was used directly for the next step. **^{1}H NMR** (400 MHz, DMSO-d$_6$): δ [ppm] = 12.83 (br s, 1H), 7.63 – 7.59 (m, 1H), 7.57 – 7.53 (m, 1H), 7.24 – 7.18 (m, 1H); **^{13}C NMR** (100 MHz, DMSO-d$_6$): δ [ppm] = 153.9, 143.6, 133.9, 126.9, 122.4, 115.5, 114.2, 92.5; **HRMS** (ESI): calculated for $[C_8H_3N_2O_2]^-$: *m/z* = 159.0200, found: *m/z* = 159.0199.

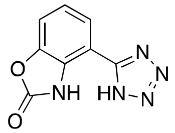

4-(1*H*-tetrazol-5-yl)benzo[*d*]oxazol-2(3*H*)-one (21)

Compound **s4** (90.0 mg, 0.56 mmol, 1.00 equiv), NaN_3 (73.0 mg, 1.10 mmol, 2.00 equiv) and NH_4Cl (60.0 mg, 1.10 mmol, 2.00 equiv) were suspended in anhydrous DMF (2 mL) and the resulting mixture was stirred at 110 °C for 2 h (progress of the reaction monitored by TLC). H_2O (10 mL) was added to obtain a clear solution. 6 M HCl was added dropwise until a slightly acidic pH was reached and a colorless solid crushed out. The colorless solid was collected by filtration, washed with H_2O (3 x 20 mL) and dried under high-vacuum. Purification by column chromatography to obtain the desired product as a yellow solid (EtOAc:MeOH 100:1 to 10:1). Yield: 40 mg (35%). **^{1}H NMR** (400 MHz, CD_3OD): δ [ppm] = 7.76 (dd, J = 8.0, 1.1 Hz, 1H), 7.34 (dd, J = 8.0, 1.1 Hz, 1H), 7.26 (t, J = 8.0 Hz, 1H); **^{13}C NMR** (100 MHz, CD_3OD): δ [ppm] = 156.1, 155.2, 144.5, 128.4, 122.2, 121.3, 110.6, 109.5; **HRMS** (ESI): calculated for $[C_8H_6N_5O_2]^+$: *m/z* = 204.0516, found: *m/z* = 204.0518.

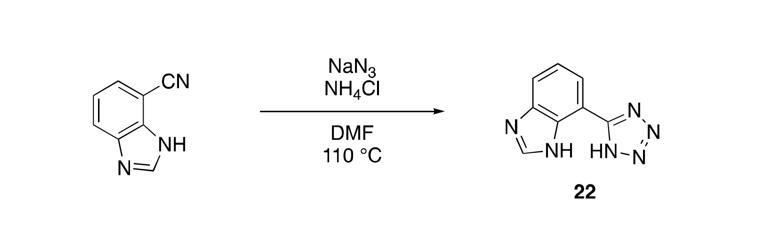

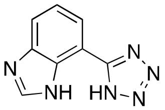

7-(1*H*-tetrazol-5-yl)-1*H*-benzo[*d*]imidazole (22)

1*H*-benzo[*d*]imidazole-7-carbonitrile (110 mg, 0.77 mmol, 1.00 equiv), NaN_3 (99.9 mg, 1.54 mmol, 2.00 equiv) and NH_4Cl (82.2 mg, 1.54 mmol, 2.00 equiv) were suspended in anhydrous DMF (2.5 mL) and the resulting mixture was stirred at 110 °C for 24 h (progress of the reaction

monitored by TLC). The mixture was concentrated under reduced pressure and H_2O (10 mL) was added. A solid crushed out which was collected by filtration, washed with H_2O (3x20 mL) and dried under high-vacuum. Purification by C18 column chromatography (H_2O:MeOH 10:1 to 1:10) to obtain the desired product as a greyish solid. Yield: 45 mg (31%). **^{1}H NMR** (400 MHz, DMSO-d$_6$): δ [ppm] = 8.42 (s, 1H), 7.94 (d, J = 7.5 Hz, 1H), 7.82 (d, J = 8.0 Hz, 1H), 7.43 (t, J = 7.8 Hz, 1H); **^{13}C NMR** (100 MHz, DMSO-d$_6$): δ [ppm] = 153.4, 145.0, 137.7, 134.4, 122.5, 121.4, 118.3, 111.2; **HRMS** (ESI): calculated for $[C_8H_7N_6]^+$: *m/z* = 187.0727, found: *m/z* = 187.0728.

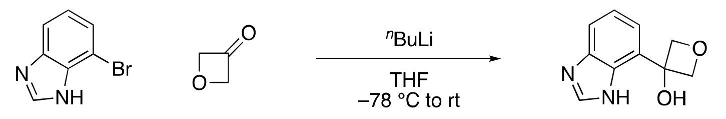

23

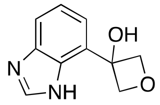

3-(1*H*-Benzo[*d*]imidazol-7-yl)oxetan-3-ol (23)

7-bromo-1*H*-benzo[*d*]imidazole (242 mg, 1.23 mmol, 1.00 equiv) was dissolved in anhydrous THF (6 mL) and ^{n}BuLi (1.6 M in hexanes, 2.70 mmol, 2.20 equiv) was added dropwise at –78 °C. The resulting mixture was stirred at this temperature for 2 h. Oxetane-3-one (121 mg, 1.68 mmol, 1.20 equiv) in THF (2 mL) was added dropwise. The resulting mixture was allowed to warm to room temperature and stirred for 24 h. Sat. NH_4Cl (10 mL) was added and the mixture was extracted with EtOAc (3x20 mL). The combined organics were washed with brine, dried over $MgSO_4$ and concentrated under reduced pressure. Purification by column chromatography (CH_2Cl_2:MeOH 100:1 to 10:1) to obtain the desired product as a colorless solid. Yield: 70 mg (30%). **^{1}H NMR** (400 MHz, CD_3OD): δ [ppm] = 8.14 (s, 1H), 7.61 (d, J = 7.9 Hz, 1H), 7.50 – 7.44 (m, 1H), 7.31 (t, J = 7.8 Hz, 1H), 5.10 – 5.00 (m, 4H); **^{13}C NMR** (100

MHz, CD_3OD): δ [ppm] =142.6, 141.6, 134.0, 130.3, 123.5, 120.2, 117.3, 86.7, 76.6; **HRMS** (ESI): calculated for $[C_{10}H_{11}N_2O_2]^+$: *m/z* = 191.0815, found: *m/z* = 191.0815.

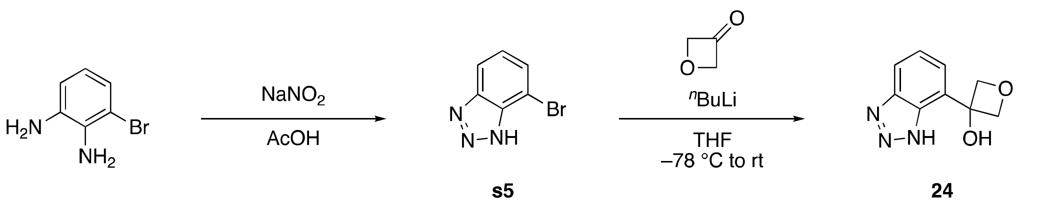

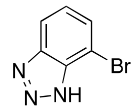

7-bromo-1*H*-benzo[*d*][1,2,3]triazole (s5)

3-bromobenzene-1,2-diamine (500 mg, 2.67 mmol, 1.00 equiv) was dissolved in H_2O (5 mL) and AcOH (2 mL) and stirred for 5 min. Thereto, a solution of $NaNO_2$ (211 mg, 3.06 mmol, 1.11 equiv) in H_2O (2 mL) was added dropwise. The resulting suspension was stirred for 18 h at room temperature. The brown solid was filtered off, washed with H_2O (3 x 20 mL) and dried under high-vacuum to obtain the desired product, which was used without any further purifications for the next step. **^{1}H NMR** (400 MHz, Acetone-d_6): δ [ppm] = 7.91 (d, J = 8.3 Hz, 1H), 7.67 (d, J = 7.4 Hz, 1H), 7.42 (dd, J = 8.3, 7.4 Hz, 1H); **^{13}C NMR** (100 MHz, Acetone-d_6): δ [ppm] = 140.9, 139.3, 128.7, 128.1, 114.3, 109.5; **HRMS** (ESI): calculated for $[C_6H_5BrN_3]^+$: *m/z* = 197.9661, found: *m/z* = 197.9659.

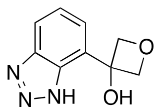

3-(1*H*-Benzo[*d*][1,2,3]triazol-7-yl)oxetan-3-ol (24)

Compound **s6** (242 mg, 1.22 mmol, 1.00 equiv) was dissolved in anhydrous THF (4 mL) and ^{n}BuLi (1.6 M in hexanes, 3.67 mmol, 3.00 equiv) was added dropwise at –78 °C. The resulting mixture was stirred at this temperature for 2 h. Oxetane-3-one (264 mg, 3.67 mmol, 3.00 equiv) in THF (2 mL) was added dropwise. The resulting mixture was allowed to warm to

room temperature and stirred for 24 h. Sat. NH_4Cl (10 mL) was added and the mixture was extracted with EtOAc (3x20 mL). The combined organics were washed with brine, dried over $MgSO_4$ and concentrated under reduced pressure. Purification by column chromatography (CH_2Cl_2:MeOH 100:1 to 10:1) to obtain the desired product as a colorless solid. Yield: 130 mg (56%). **^{1}H NMR** (400 MHz, CD_3OD): δ [ppm] = 7.90 – 7.82 (m, 1H), 7.70 – 7.64 (m, 1H), 7.55 – 7.44 (m, 1H), 5.19 – 5.00 (m, 4H); **^{13}C NMR** (100 MHz, CD_3OD): δ [ppm] = 142.9, 135.4, 131.1, 126.5, 123.3, 116.5, 86.2, 76.0; **HRMS** (ESI): calculated for $[C_9H_{10}N_3O_2]^+$: *m/z* = 192.0768, found: *m/z* = 192.0771.

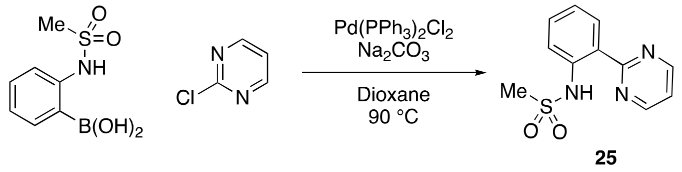

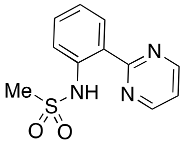

***N*-(2-(Pyrimidin-2-yl)phenyl)methanesulfonamide (25)**

(2-(Methylsulfonamido)phenyl)boronic acid (258 mg, 1.20 mmol, 1.20 equiv), 2-chloro-pyrimidine (115 mg, 1.00 mmol, 1.00 equiv) and $Pd(PPh_3)_2Cl_2$ (1.5 mol%) were suspended in Dioxane (2.5 mL). Thereto, 2 M Na_2CO_3 (2.5 mL) was added and the resulting mixture was stirred at 90 °C for 5 h (progress of the reaction monitored by TLC). H_2O (5 mL) and EtOAc (15 mL) were added, the phases were separated and the aqueous phase was extracted with EtOAc (3x10 mL). The combined organic phases were washed with brine, dried over $MgSO_4$ and concentrated under reduced pressure. Purification by column chromatography (hexanes:EtOAc 10:1 to 1:1) followed by C18 column chromatography (H_2O:MeOH 10:1 to 1:10 + 0.1 % HCO_2H) to obtain the desired product as a colourless solid. Yield: 180 mg (32%). **^{1}H NMR** (400 MHz, $CDCl_3$): δ [ppm] = 12.51 (br s, 1H), 8.86 (d, J = 4.9 Hz, 2H), 8.70

– 8.63 (m, 1H), 7.82 – 7.71 (m, 1H), 7.54 – 7.43 (m, 1H), 7.28 (t, J = 4.9 Hz, 1H), 7.26 – 7.18 (m, 1H), 3.92 (s, 3H); **^{13}C NMR** (100 MHz, $CDCl_3$): *δ* [ppm] = 164.4, 156.7, 139.4, 132.8, 131.2, 123.5, 122.7, 119.1, 118.9, 39.8; **HRMS** (ESI): calculated for $[C_{11}H_{12}N_3O_2S]^+$: *m/z* = 250.0645, found: *m/z* = 250.0647.

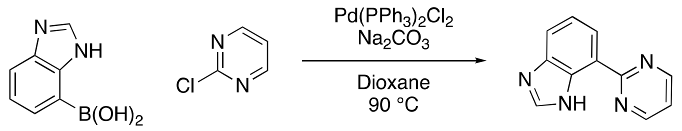

26

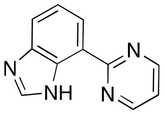

***N*-(2-(Pyrimidin-2-yl)phenyl)methanesulfonamide (26)**

(1*H*-Benzo[*d*]imidazol-7-yl)boronic acid (150 mg, 0.93 mmol, 1.20 equiv), 2-chloro-pyrimidine (90.0 mg, 0.78 mmol, 1.00 equiv) and $Pd(PPh_3)_2Cl_2$ (1.5 mol%) were suspended in Dioxane (2 mL). Thereto, 2 M Na_2CO_3 (2 mL) was added and the resulting mixture was stirred at 90 °C for 5 h (progress of the reaction monitored by TLC). H_2O (5 mL) and EtOAc (15 mL) were added, the phases were separated and the aqueous phase was extracted with EtOAc (3x10 mL). The combined organic phases were washed with brine, dried over $MgSO_4$ and concentrated under reduced pressure. Purification by column chromatography (hexanes:EtOAc 10:1 to 1:1) followed by C18 column chromatography (H_2O:MeOH 10:1 to 1:10 + 0.1% HCO_2H) to obtain the desired product as a colourless solid. Yield: 30 mg (20%). **^{1}H NMR** (400 MHz, Acetone-d_6): *δ* [ppm] = 12.36 (br s, 1H), 8.94 (d, *J* = 4.8 Hz, 2H), 8.50 – 8.44 (m, 1H), 8.29 (s, 1H), 7.92 – 7.86 (m, 1H), 7.43 – 7.36 (m, 2H); **^{13}C NMR** (100 MHz, Acetone-d_6): *δ* [ppm] = 164.8, 158.3, 145.5, 143.1, 133.5, 123.5, 123.4, 122.4, 122.3, 119.9; **HRMS** (ESI): calculated for $[C_{11}H_9N_4]^+$: *m/z* = 197.0822, found: *m/z* = 197.0820.

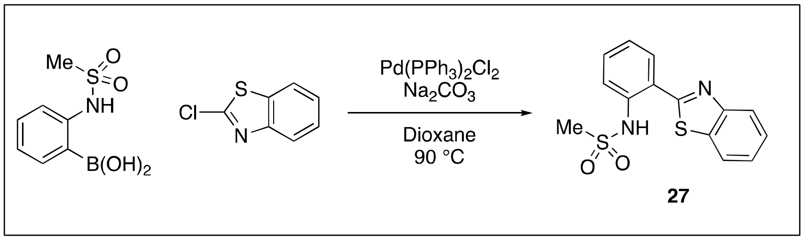

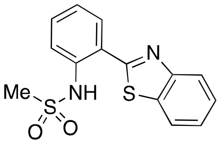

***N*-(2-(Benzo[*d*]thiazol-2-yl)phenyl)methanesulfonamide (27)**

(2-(Methylsulfonamido)phenyl)boronic acid (258 mg, 1.20 mmol, 1.20 equiv), 2-chlorobenzo[*d*]thiazole (170 mg, 1.00 mmol, 1.00 equiv) and $Pd(PPh_3)_2Cl_2$ (1.5 mol%) were suspended in Dioxane (2.5 mL). Thereto, 2 M Na_2CO_3 (2.5 mL) was added and the resulting mixture was stirred at 90 °C for 5 h (progress of the reaction monitored by TLC). H_2O (5 mL) and EtOAc (15 mL) were added, the phases were separated and the aqueous phase was extracted with EtOAc (3x10 mL). The combined organic phases were washed with brine, dried over $MgSO_4$ and concentrated under reduced pressure. Purification by column chromatography (hexanes:EtOAc 10:1 to 1:1) followed by C18 column chromatography (H_2O:MeOH 10:1 to 1:10 + 0.1% HCO_2H) to obtain the desired product as a colourless solid. Yield: 100 mg (33%). **^{1}H NMR** (400 MHz, $CDCl_3$): δ [ppm] = 12.17 (br s, 1H), 8.11 – 8.04 (m, 1H), 7.96 – 7.80 (m, 3H), 7.58 – 7.40 (m, 3H), 7.25 – 7.18 (m, 1H), 3.07 (s, 3H); **^{13}C NMR** (100 MHz, $CDCl_3$): δ [ppm] = 168.2, 152.6, 137.5, 133.2, 132.3, 130.5, 127.0, 126.2, 123.7, 123.3, 121.5, 119.7, 119.0, 40.0; **HRMS** (ESI): calculated for $[C_{14}H_{13}N_2O_2S_2]^+$: m/z = 305.0413, found: m/z = 305.0416.

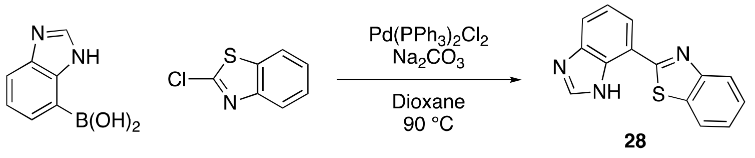

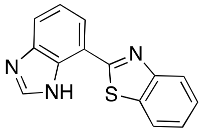

2-(1*H*-Benzo[*d*]imidazol-7-yl)benzo[*d*]thiazole (28)

(1*H*-Benzo[*d*]imidazol-7-yl)boronic acid (150 mg, 0.93 mmol, 1.20 equiv), 2-chlorobenzo[*d*]thiazole (133 mg, 0.78 mmol, 1.00 equiv) and $Pd(PPh_3)_2Cl_2$ (1.5 mol%) were suspended in Dioxane (2 mL). Thereto, 2 M Na_2CO_3 (2 mL) was added and the resulting mixture was stirred at 90 °C for 5 h (progress of the reaction monitored by TLC). H_2O (5 mL) and EtOAc (15 mL) were added, the phases were separated and the aqueous phase was extracted with EtOAc (3x10 mL). The combined organic phases were washed with brine, dried over $MgSO_4$ and concentrated under reduced pressure. Purification by column chromatography (hexanes:EtOAc 10:1 to 1:1) followed by C18 column chromatography (H_2O:MeOH 10:1 to 1:10 + 0.1% HCO_2H) to obtain the desired product as a colourless solid. Yield: 80 mg (40%). **^{1}H NMR** (400 MHz, benzene-d$_6$): δ [ppm] = 10.75 (br s, 1H), 8.05 – 7.99 (m, 2H), 7.58 – 7.53 (m, 1H), 7.45 – 7.41 (m, 1H), 7.40 (s, 1H), 7.26 – 7.21 (m, 1H), 7.07 – 6.97 (m, 2H); **^{13}C NMR** (100 MHz, benzene-d$_6$): δ [ppm] =167.4, 154.3, 144.8, 141.8, 134.3, 131.5, 126.6, 125.5, 123.9, 122.9, 122.8, 122.4, 122.0, 117.6; **HRMS** (ESI): calculated for $[C_{14}H_{10}N_3S]^+$: *m/z* = 252.0590, found: *m/z* = 252.0592.

3. Preparation of MBI 11 derivatives

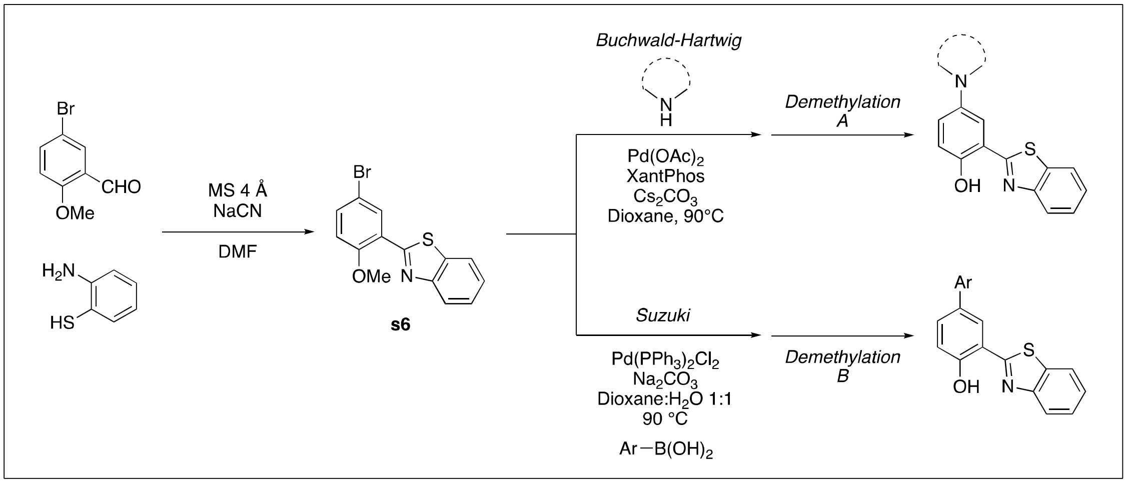

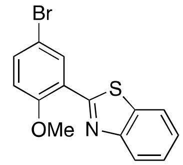

2-(5-bromo-2-methoxyphenyl)benzo[*d*]thiazole (s6)

5-Bromo-2-methoxybenzaldehyde (2.00 g, 8.00 mmol, 1.00 equiv) and 2-aminothiophenol (1.00 g, 8.00 mmol, 1.00 equiv) were dissolved in DMF (15 mL) and MS 4Å was added. The resulting mixture was stirred at 60 °C for 14 h. The mixture was allowed to cool to room temperature and NaCN (40.0 mg, 0.80 mmol, 0.10 equiv) was added as a solid. The resulting mixture was stirred for 14 h (open flask). All volatiles were removed under reduced pressure and the resulting oil was purified by column chromatography (hexanes:EtOAc 10:1) to obtain the desired product as a colourless solid. Yield: 2.2 g (90%). **^{1}H NMR** (400 MHz, $CDCl_3$): *δ* [ppm] = 8.70 – 8.65 (m, 1H), 8.12 – 8.07 (m, 1H), 7.96 – 7.88 (m, 1H), 7.56 – 7.46 (m, 2H), 7.43 – 7.33 (m, 1H), 6.93 (d, *J* = 8.8 Hz, 1H), 4.03 (s, 3H); **^{13}C NMR** (100 MHz, $CDCl_3$): *δ* [ppm] = 161.6, 156.3, 152.0, 136.2, 134.4, 131.9, 126.3, 125.1, 124.0, 123.0, 121.4, 113.9, 113.5, 56.1; **HRMS** (ESI): calculated for $[C_{14}H_{11}BrNOS]^+$: *m/z* = 319.9739, found: *m/z* = 319.9735.

General conditions for Buchwald-Hartwig cross-coupling:

Compound **s6** (100 mg, 0.31 mmol, 1.00 equiv), amine (0.31 mmol, 1.00 equiv), $Pd(OAc)_2$ (5.60 mg, 25.0 µmol, 0.08 equiv), XantPhos (22.0 mg, 37.5 µmol, 0.12 equiv) and Cs_2CO_3 (204 mg, 0.62 mmol, 2.00 equiv) were mixed in dioxane (1.0 mL). The resulting mixture was degassed (Argon bubbling) for 15 min and stirred at 90 °C for 14 h. The mixture was allowed to cool to room temperature, H_2O (5 mL) and EtOAc (15 mL) were added, the phases were separated and the aqueous phase was extracted with EtOAc (3x10 mL). The combined organic phases were washed with brine, dried over $MgSO_4$ and concentrated under reduced pressure. Purification by column chromatography.

General conditions for Suzuki cross-coupling:

Compound **s6** (50.0 mg, 0.16 mmol, 1.00 equiv) and boronic acid (0.19 mmol, 1.20 equiv) were mixed in dioxane (0.5 mL) and aq. Na_2CO_3 (2 M, 0.5 mL) was added. The resulting mixture was degassed (Argon bubbling) for 15 min and stirred at 90 °C for 14 h. The mixture was allowed to cool to room temperature, H_2O (5 mL) and EtOAc (15 mL) were added, the phases were separated and the aqueous phase was extracted with EtOAc (3x10 mL). The combined organic phases were washed with brine, dried over $MgSO_4$ and concentrated under reduced pressure. Purification by column chromatography.

General conditions for demethylation A:

Phenol ether (1.00 equiv) was dissolved in CH_2Cl_2 (1.0 mL) and BBr_3 (1 M in heptane, 4.00 equiv) was added dropwise at 0 °C. The resulting mixture was stirred for 14 h at room temperature. Sat. aq. Na_2CO_3 (5 mL) and EtOAc (15 mL) were added, the phases were separated and the aqueous phase was extracted with EtOAc (3x10 mL). The combined organic phases were washed with brine, dried over $MgSO_4$ and concentrated under reduced pressure. Purification by column chromatography.

General conditions for demethylation B:

Phenol ether (1.00 equiv) and pyridine hydrochloride (4.00 equiv) were heated to 200 °C and stirred at this temperature for 2 h. The mixture was allowed to cool to room temperature and the residue was dissolved in EtOAc (15 mL), washed with sat. $NaHCO_3$ (5 mL), brine (5 mL), dried over $MgSO_4$ and concentrated under reduced pressure. Purification by column chromatography.

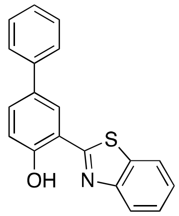

3-(benzo[*d*]thiazol-2-yl)-[1,1'-biphenyl]-4-ol (11a)

Prepared according to the *general conditions for Suzuki cross-coupling* and *general conditions for demethylation B*. Yield (over two steps): 30 mg (71%). **^{1}H NMR** (400 MHz, $CDCl_3$): δ [ppm] = 12.58 (br s, 1H), 8.05 – 7.97 (m, 1H), 7.95 – 7.91 (m, 1H), 7.90 – 7.87 (m, 1H), 7.65 – 7.59 (m, 3H), 7.55 – 7.50 (m, 1H), 7.50 – 7.40 (m, 3H), 7.40 – 7.35 (m, 1H), 7.19 (d, *J* = 8.6 Hz, 1H); **^{13}C NMR** (100 MHz, $CDCl_3$): δ [ppm] = 169.4, 157.6, 153.9, 151.9, 140.2, 133.0, 132.7, 131.8, 129.1, 127.3, 126.9, 126.8, 125.8, 122.4, 121.7, 118.5, 117.0; **HRMS** (ESI): calculated for $[C_{19}H_{14}NOS]^+$: *m/z* = 304.0791, found: *m/z* = 304.0790.

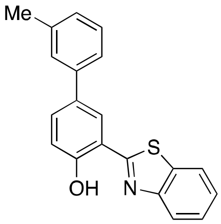

3-(benzo[*d*]thiazol-2-yl)-3'-methyl-[1,1'-biphenyl]-4-ol (11b)

Prepared according to the *general conditions for Suzuki cross-coupling* and *general conditions for demethylation B*. Yield (over two steps): 30 mg (67%). **^{1}H NMR** (400 MHz, $CDCl_3$): δ [ppm] = 8.04 – 8.00 (m, 1H), 7.95 – 7.90 (m, 1H), 7.90 – 7.86 (m, 1H), 7.64 – 7.59 (m, 1H),

7.56 – 7.50 (m, 1H), 7.46 – 7.39 (m, 3H), 7.38 – 7.34 (m, 1H), 7,18 (d, *J* = 8.5 Hz, 2H), 2.45 (s, 3H); **^{13}C NMR** (100 MHz, $CDCl_3$): *δ* [ppm] = 169.5, 157.5, 151.8, 140.2, 138.7, 133.2, 132.9, 132.7, 131.9, 129.0, 128.0, 127.7, 126.9, 125.8, 124.0, 122.3, 121.7, 118.4, 117.0, 21.7; **HRMS** (ESI): calculated for $[C_{20}H_{16}NOS]^-$: *m/z* = 304.0791, found: *m/z* = 304.0790.

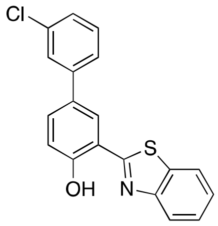

3-(benzo[*d*]thiazol-2-yl)-3'-chloro-[1,1'-biphenyl]-4-ol (11c)

Prepared according to the *General conditions for Suzuki cross-coupling* and *General conditions for demethylation B*. Yield (over two steps): 30 mg (57%). **^{1}H NMR** (400 MHz, $CDCl_3$): *δ* [ppm] = 8.03 – 7.99 (m, 1H), 7.95 – 7.90 (m, 1H), 7.85 – 7.82 (m, 1H), 7.61 – 7.44 (m, 2H), 7.55 – 7.50 (m, 1H), 7.48 – 7.36 (m, 3H), 7.34 – 7.29 (m, 1H), 7.18 (d, *J* = 8.6 Hz, 1H); **^{13}C NMR** (100 MHz, $CDCl_3$): *δ* [ppm] = 169.1, 158.0, 151.9, 142.0, 134.9, 132.7, 131.6, 131.5, 130.3, 127.2, 127.0, 126.9, 126.8, 125.9, 125.0, 122.4, 121.7, 118.7, 117.1; **HRMS** (ESI): calculated for $[C_{19}H_{11}ClNOS]^-$: *m/z* = 336.0255, found: *m/z* = 336.0253.

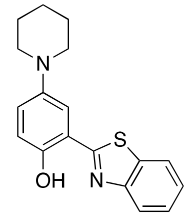

2-(benzo[*d*]thiazol-2-yl)-4-(piperidin-1-yl)phenol (11d)

Prepared according to the *General conditions for Buchwald-Hartwig cross-coupling* and *General conditions for demethylation A*. Yield (over two steps): 40 mg (41%). **^{1}H NMR** (400 MHz, $CDCl_3$): *δ* [ppm] = 12.13 (br s, 1H), 8.00 – 7.95 (m, 1H), 7.93 – 7.86 (m, 1H), 7.52 – 7.45 (m, 1H), 7.43 – 7.37 (m, 1H), 7.27 – 7.17 (m, 1H), 7.13 – 7.07 (m, 1H), 7.03 (d, *J* = 9.0 Hz, 1H), 3.16 – 2.99 (m, 4H), 1.88 – 1.68 (m, 4H), 1.67 – 1.51 (m, 2H); **^{13}C NMR** (100 MHz,

$CDCl_3$): *δ* [ppm] = 169.7, 152.4, 152.1, 145.9, 132.7, 126.8, 125.5, 124.3, 122.3, 121.6, 118.4, 116.5, 52.6, 26.2, 24.2; **HRMS** (ESI): calculated for $[C_{18}H_{19}N_2OS]^+$: *m/z* = 311.1213, found: *m/z* = 311.1214.

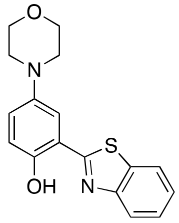

2-(benzo[*d*]thiazol-2-yl)-4-morpholinophenol (11e)

Prepared according to the *General conditions for Buchwald-Hartwig cross-coupling* and *General conditions for demethylation A*. Yield (over two steps): 25 mg (28%). ^{1}**H NMR** (400 MHz, $CDCl_3$): *δ* [ppm] = 12.16 (br s, 1H), 8.02 – 7.96 (m, 1H), 7.93 – 7.88 (m, 1H), 7.54 – 7.48 (m, 1H), 7.44 – 7.39 (m, 1H), 7.21 – 7.13 (m, 1H), 7.12 – 7.03 (m, 2H), 4.01 – 3.74 (m, 4H), 3.26 – 2.90 (m, 4H); ^{13}**C NMR** (100 MHz, $CDCl_3$): *δ* [ppm] = 169.4, 152.7, 152.1, 144.7, 132.7, 126.9, 125.7, 123.2, 122.3, 121.7, 118.7, 116.6, 115.6, 67.1, 51.1; **HRMS** (ESI): calculated for $[C_{17}H_{17}N_2O_2S]^+$: *m/z* = 313.1005, found: *m/z* = 313.1002.

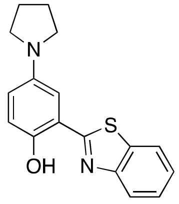

2-(benzo[*d*]thiazol-2-yl)-4-(pyrrolidin-1-yl)phenol (11f)

Prepared according to the *General conditions for Buchwald-Hartwig cross-coupling* and *General conditions for demethylation A*. Yield (over two steps): 35 mg (39%). ^{1}**H NMR** (400 MHz, $CDCl_3$): *δ* [ppm] = 11.81 (br s, 1H), 8.00 – 7.96 (m, 1H), 7.91 – 7.86 (m, 1H), 7.52 – 7.46 (m, 1H), 7.42 – 7.36 (m, 1H), 7.06 – 7.00 (m, 1H), 6.84 – 6.70 (m, 2H), 3.48 – 3.13 (m, 4H), 2.18 – 1.90 (m, 4H); ^{13}**C NMR** (100 MHz, $CDCl_3$): *δ* [ppm] = 170.00, 152.3, 149.3, 142.1,

132.8, 126.7, 125.4, 122.3, 121.6, 118.6, 118.1, 116.7, 109.4, 48.4, 25.5; **HRMS** (ESI): calculated for $[C_{17}H_{17}N_2OS]^+$: *m/z* = 297.1056, found: *m/z* = 297.1055.

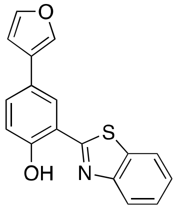

2-(benzo[*d*]thiazol-2-yl)-4-(furan-3-yl)phenol (11g)

Prepared according to the *General conditions for Suzuki cross-coupling* and *General conditions for demethylation A*. Yield (over two steps): 15 mg (33%). **^{1}H NMR** (400 MHz, $CDCl_3$): *δ* [ppm] = 12.56 (br s, 1H), 8.03 – 7.99 (m, 1H), 7.95 – 7.89 (m, 1H), 7.78 – 7.71 (m, 2H), 7.56 – 7.48 (m, 3H), 7.46 – 7.41 (m, 1H), 7.16 – 7.10 (m, 1H), 6.74 – 6.69 (m, 1H); **^{13}C NMR** (100 MHz, $CDCl_3$): *δ* [ppm] = 169.2, 157.2, 151.9, 143.9, 138.1, 132.7, 130.7, 126.9, 125.8, 125.6, 125.5, 124.3, 122.4, 121.7, 118.5, 117.0, 109.0; **HRMS** (ESI): calculated for $[C_{17}H_{12}NO_2S]^+$: *m/z* = 294.0583, found: *m/z* = 294.0582.

4. Computational docking

The geometry of the molecule was determined using density-functional theory (DFT) calculations with the Gaussian software package (Gaussian, Inc., Wallingford CT, 2016). The calculations were performed using the Pople double-zeta basis set with a single set of polarization functions on non-hydrogen atoms (6-31G(d)). Solvent effects were included using a polarizable continuum model (PCM). The structures of all calculated molecules correspond to minima on the ground state potential energy surfaces with no imaginary frequencies present. The protein structure (PDB: 3VW9)[4] was downloaded from the Protein Data Bank (https://www.rcsb.org/) and prepared for the docking experiment using the Molecular Operating Environment (MOE, Chemical Computing Group ULC, Montreal, QC, Canada, 2019) software package. Water molecules, other small molecules, inhibitors, and ions were removed. Hydrogen atoms were added and the side chains protonated at physiological pH. The structurally optimized molecules were docked using the Genetic Optimization for Ligand Docking (GOLD, Cambridge Crystallographic Data Centre, Cambridge, United Kingdom, 2019) software package. The protein structure was considered rigid. The binding pose of the compounds was predicted using a genetic algorithm with a population size of 200, selection pressure of 1.2, number of operations of 500000, number of islands of 5, niche size of 2, crossover frequency of 95, mutation frequency of 95, and a migration frequency of 10. The genetic algorithm was set to 100 runs. During the docking procedure the binding poses were evaluated using the ChemPLP scoring function. After docking, the obtained solutions were re-scored using the GoldScore fitness function.

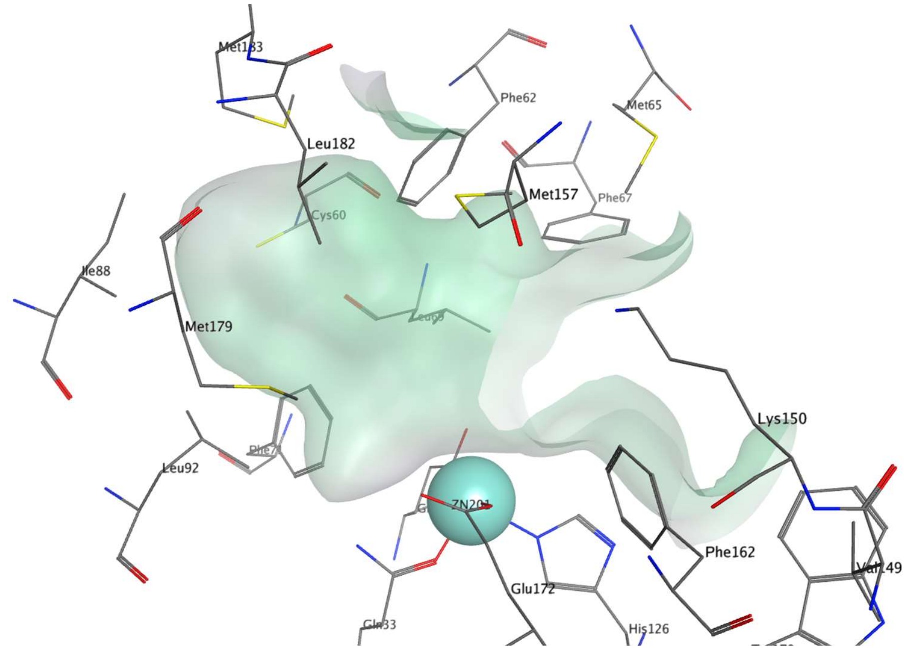

Figure S1. GLO1 active site without inhibitor bound.

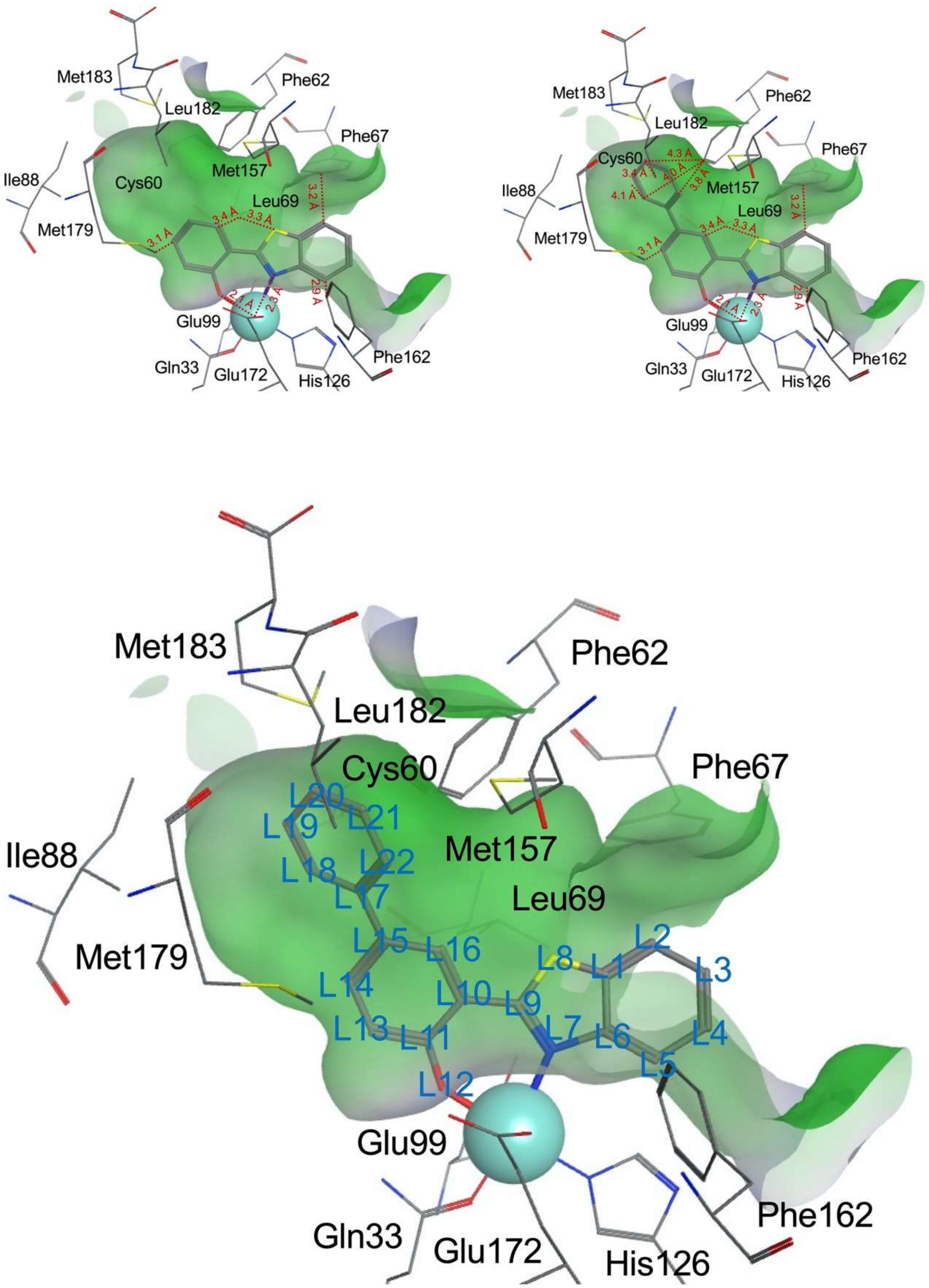

Figure S2. GLO1 active site with inhibitors **11** (*top left*) and **11a** (*top right*) bound and contacts shown; with atom labeling (*bottom*) (see Table S4).

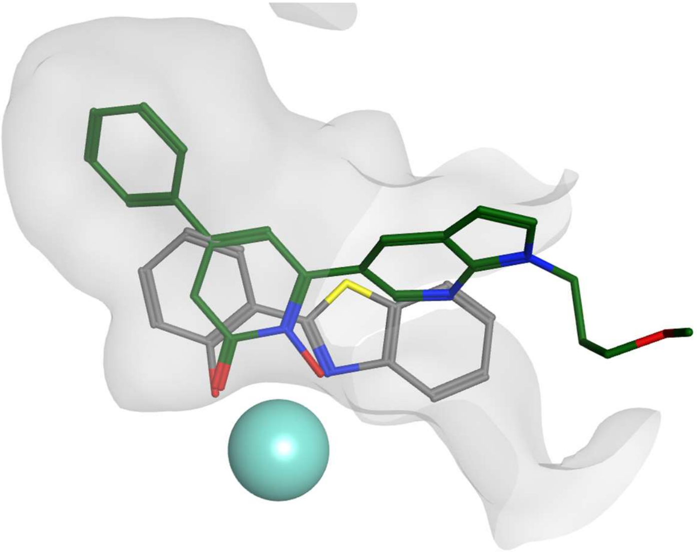

Figure S3. GLO1 active site with inhibitor **11** and literature known inhibitor **Chugai-3d**[4] show similar binding mode to the Zn^{2+} cation.

5. Single Point screening

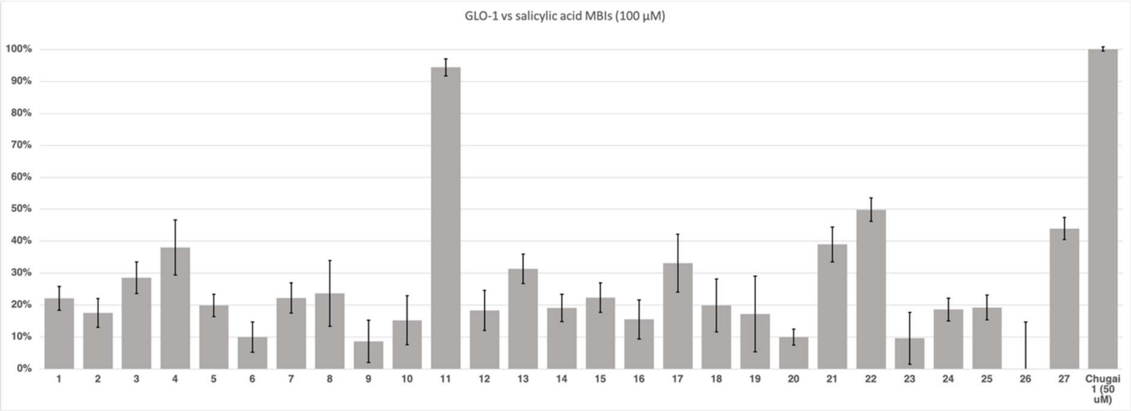

Figure S4. Single point screening of salicylate MBI library against GLO1 at 100 µM inhibitor concentration. **Chugai 1** (4,6-diphenyl-N-hydroxypyridone)[4] as positive control.

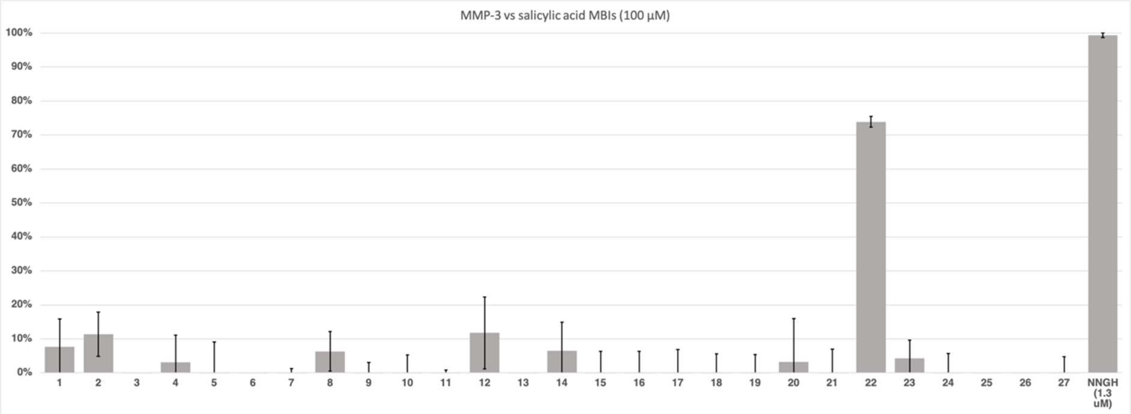

Figure S5. Single point screening of salicylate MBI library against MMP-3 at 100 µM inhibitor concentration. NNGH as positive control.

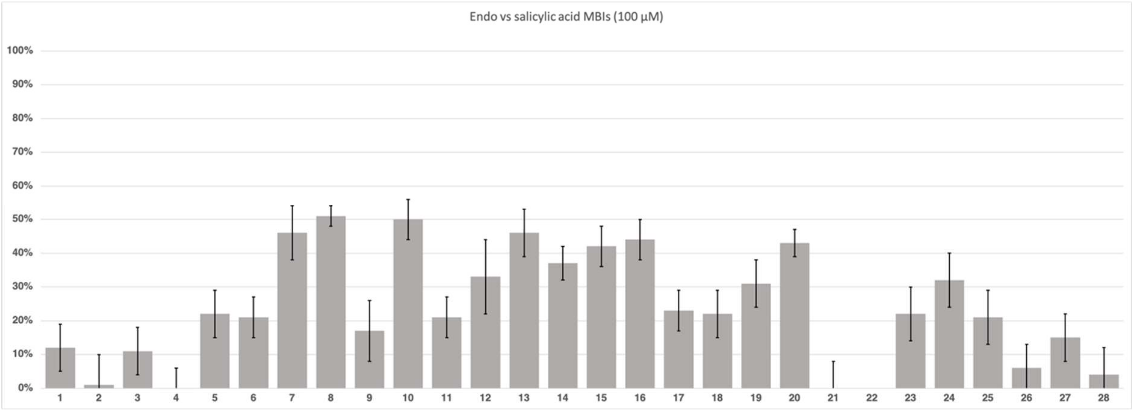

Figure S6. Single point screening of salicylate MBI library against Endo at 100 µM inhibitor concentration.

6. Dose-response curves against GLO1

The raw data from the GLO1 assay was background corrected and normalized from 0 to 100 with 100 being the largest mean of each dataset. A variable slope log-logistic model was chosen for curve fitting. Some compounds did not dissolve well at higher concentrations (>50 μM). The corresponding datapoints are shown as empty bullets and were not included in the curve-fitting. The resulting restricted datasets were fitted according to a literature procedure setting the lower limit of the dose-response curve to zero. This compensates for the loss of information caused by trimming of the dataset.[5,6]

11

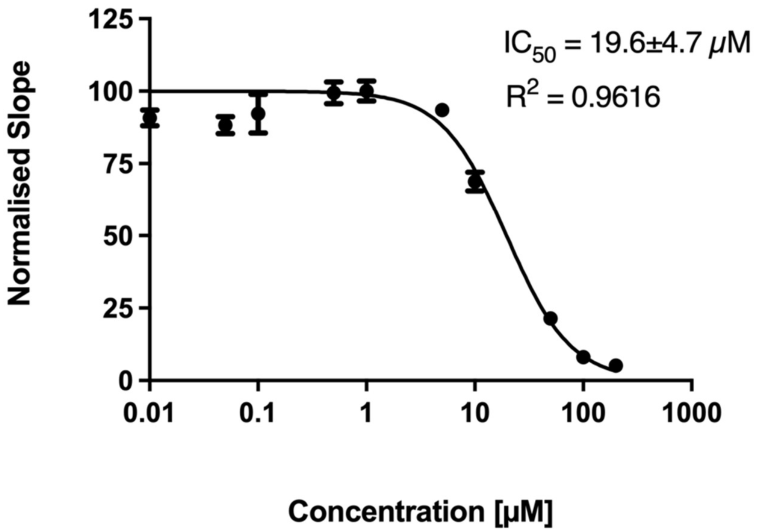

Figure S7. Dose-response curve of **11** against GLO1.

11a

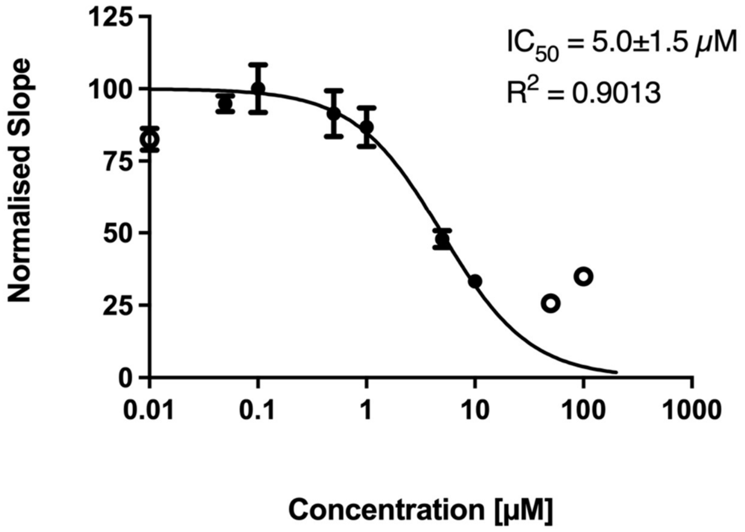

Figure S8. Dose-response curve of **11a** against GLO1.

11b

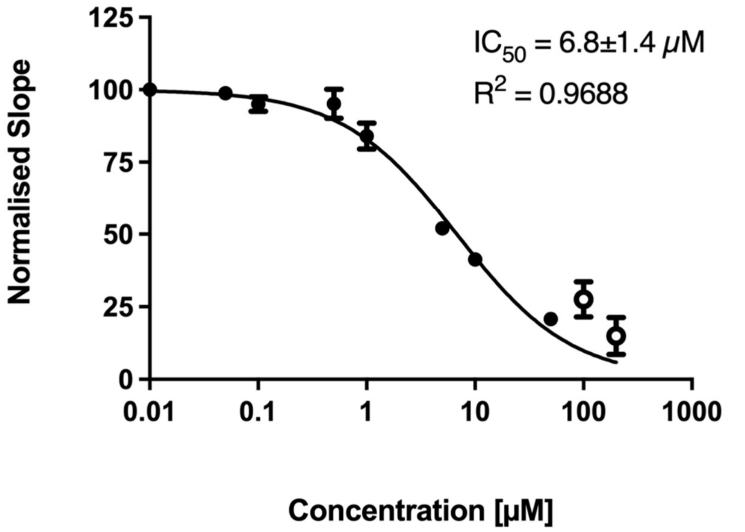

Figure S9. Dose-response curve of **11b** against GLO1.

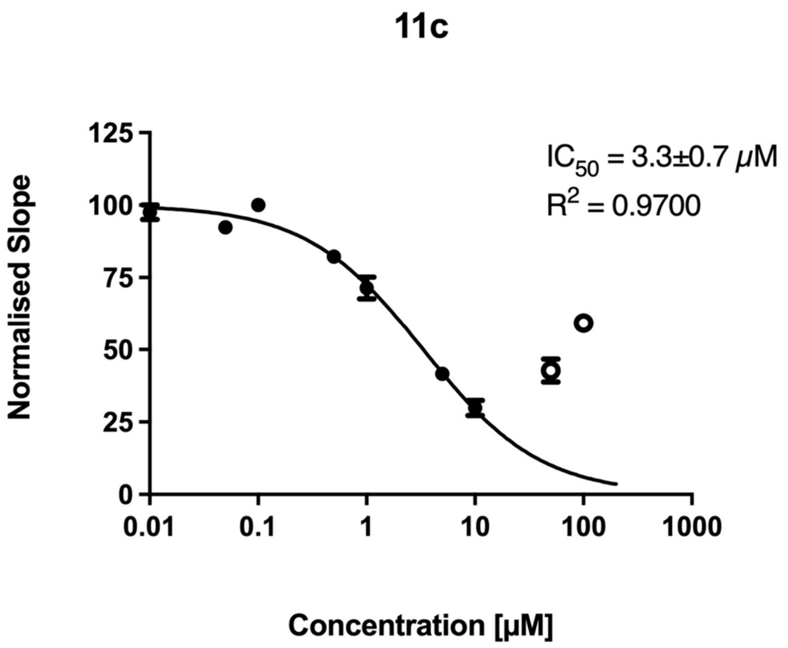

Figure S10. Dose-response curve of **11c** against GLO1.

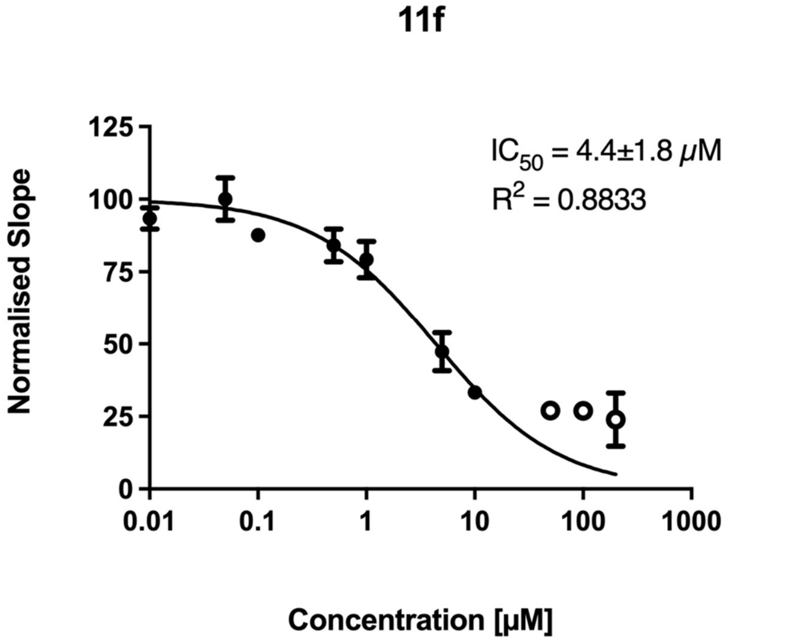

Figure S11. Dose-response curve of **11f** against GLO1.

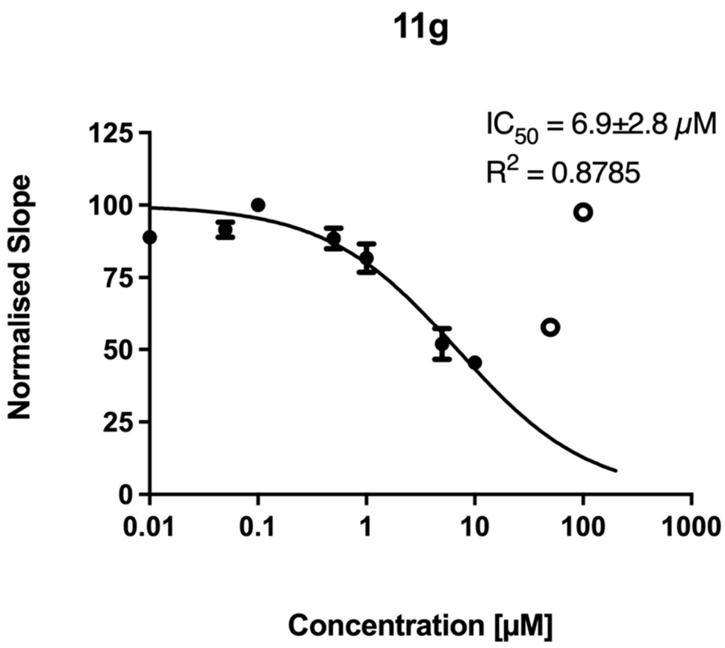

Figure S12. Dose-response curve of **11g** against GLO1.

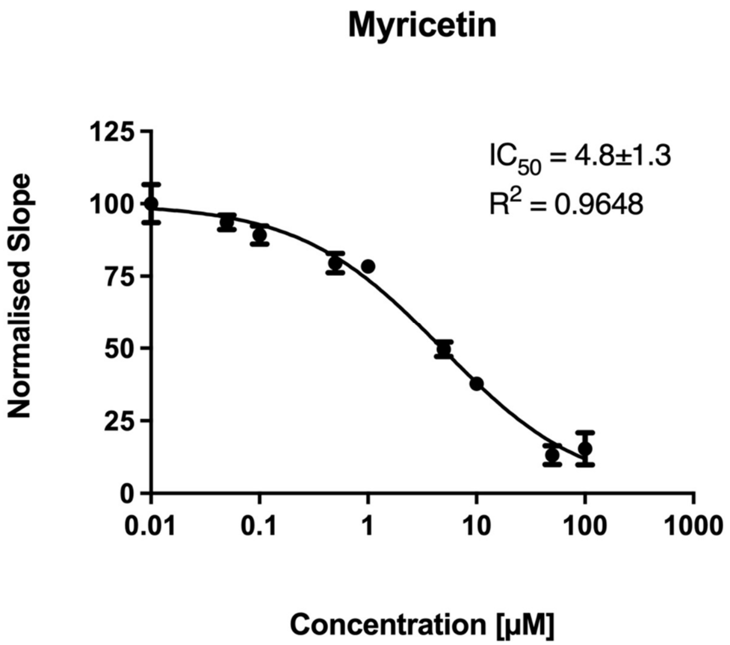

Figure S13. Dose-response curve of Myricetin against GLO1.

7. X-Ray crystallography data

Single Crystal X-ray Diffraction

Suitable crystals of [(Tp^{Ph,Me})Zn(MBI)] complexes were selected and data was collected at 100 K on a Bruker APEX-II Ultra diffractometer with a Mo-Kα Microfocus Rotating Anode and a APEX-II CCD area detector or a Bruker Kappa diffractometer equipped with a Bruker X8 APEX II Mo sealed tube and a Bruker APEX-II CCD. The data were integrated using the Bruker SAINT Software program and scaled using the SADABS software program. The structure was solved with the ShelXT[7] structure solution program using direct methods and refined with the XL[8] refinement package using least squares minimization using Olex2.[9] The crystal data files were deposited into the Cambridge Crystallographic Data Centre (CCDC). Crystallographic data collection and refinement information is listed in Table S1. Disordered solvent was treated with the PLATON SQUEEZE[10] function in [(Tp^{Ph,Me})Zn(**2**)], [(Tp^{Ph,Me})Zn(**11**)], and [(Tp^{Ph,Me})Zn(**15**)]. A pentane solvent molecule is positionally disordered in [(Tp^{Ph,Me})Zn(**3**)].

Table S1. Crystal data and structure refinement for [($Tp^{Ph,Me}$)Zn(**MBI**)] complexes.

Compound	**[($Tp^{Ph,Me}$)Zn(2)]**	**[($Tp^{Ph,Me}$)Zn(3)]**	**[($Tp^{Ph,Me}$)Zn(10)]**
Identification code	2117775	2117778	2117773
Empirical formula	$C_{38}H_{36}BN_7O_4SZn$	$C_{40.62}H_{37.25}BN_7O_4Zn$	$C_{40}H_{35}BN_8OZn$
Formula weight	762.98	763.7	719.94
Temperature/K	100	100	100
Crystal system	triclinic	monoclinic	monoclinic
Space group	P-1	P21/n	P21/n
a/Å	9.780(2)	11.6546(9)	11.4421(7)
b/Å	17.966(4)	14.6697(11)	22.4577(12)
c/Å	23.194(5)	21.3858(17)	13.4441(9)
α/°	87.678(3)	90	90
β/°	87.319(3)	97.912(3)	96.516(2)
γ/°	84.581(3)	90	90
Volume/Å^3	4050.0(16)	3621.5(5)	3432.3(4)
Z	4	4	4
ρ_{calc}g/cm^3	1.251	1.401	1.393
μ/mm^{-1}	0.704	1.373	0.762
F(000)	1584	1588	1496
Crystal size/mm^3	0.1 × 0.1 × 0.1	0.24 × 0.22 × 0.19	0.1 × 0.1 × 0.1
Radiation	MoKα (λ = 0.71073)	CuKα (λ = 1.54178)	MoKα (λ = 0.71073)
2Θ range for data collection/°	2.828 to 52.91	7.33 to 141.156	4.434 to 52.296
Index ranges	-12 ≤ h ≤ 12, -22 ≤ k ≤ 22, -28 ≤ l ≤ 27	-13 ≤ h ≤ 14, -17 ≤ k ≤ 17, -19 ≤ l ≤ 26	-14 ≤ h ≤ 14, -27 ≤ k ≤ 27, -16 ≤ l ≤ 16
Reflections collected	82013	22461	51774
Independent reflections	16482 [R_{int} = 0.0905, R_{sigma} = 0.0776]	6808 [R_{int} = 0.0401, R_{sigma} = 0.0518]	6846 [R_{int} = 0.1286, R_{sigma} = 0.0706]
Data/restraints/parameters	16482/0/945	6808/33/526	6846/0/463
Goodness-of-fit on F^2	1.097	1.087	1.038
Final R indexes [I>=2σ (I)]	R_1 = 0.0760, wR_2 = 0.1697	R_1 = 0.0418, wR_2 = 0.1122	R_1 = 0.0409, wR_2 = 0.0878
Final R indexes [all data]	R_1 = 0.1103, wR_2 = 0.1829	R_1 = 0.0478, wR_2 = 0.1168	R_1 = 0.0608, wR_2 = 0.0954
Largest diff. peak/hole / e Å^{-3}	3.29/-0.89	0.25/-0.62	0.42/-0.76

Table S1 (continued). Crystal data and structure refinement for [(Tp^{Ph,Me})Zn(**MBI**)] complexes.

Compound	**[(Tp^{Ph,Me})Zn(11)]**	**[(Tp^{Ph,Me})Zn(12)]**	**[(Tp^{Ph,Me})Zn(13)]**
Identification code	2117776	2117770	2117767
Empirical formula	$C_{43}H_{36}BN_7OSZn$	$C_{39}H_{38}BN_7O_4SZn$	$C_{39}H_{34}BN_7O_4Zn$
Formula weight	775.03	777	740.91
Temperature/K	100	100	100
Crystal system	orthorhombic	orthorhombic	triclinic
Space group	Pbca	Pna21	P-1
a/Å	16.4658(6)	11.9864(16)	11.8862(8)
b/Å	22.9868(10)	16.941(2)	12.1596(8)
c/Å	23.1548(9)	18.040(2)	13.5975(9)
α/°	90	90	115.6050(10)
β/°	90	90	90.7810(10)
γ/°	90	90	98.5870(10)
Volume/Å^3	8764.0(6)	3663.3(8)	1745.7(2)
Z	8	4	2
ρ_{calc}g/cm^3	1.175	1.409	1.41
μ/mm^{-1}	0.647	0.779	0.757
F(000)	3216	1616	768
Crystal size/mm^3	0.1 × 0.1 × 0.1	0.2 × 0.1 × 0.1	0.1 × 0.1 × 0.1
Radiation	MoKα (λ = 0.71073)	MoKα (λ = 0.71073)	MoKα (λ = 0.71073)
2Θ range for data collection/°	4.652 to 50.848	3.298 to 52.256	3.334 to 52.876
Index ranges	-19 ≤ h ≤ 19, -27 ≤ k ≤ 27, -27 ≤ l ≤ 27	-11 ≤ h ≤ 14, -20 ≤ k ≤ 20, -22 ≤ l ≤ 21	-14 ≤ h ≤ 14, -15 ≤ k ≤ 15, -17 ≤ l ≤ 17
Reflections collected	208770	20238	22366
Independent reflections	8053 [R_{int} = 0.1453, R_{sigma} = 0.0348]	6978 [R_{int} = 0.0703, R_{sigma} = 0.0973]	7180 [R_{int} = 0.0391, R_{sigma} = 0.0353]
Data/restraints/parameters	8053/0/490	6978/1/483	7180/491/473
Goodness-of-fit on F^2	1.06	0.76	1.06
Final R indexes [I>=2σ (I)]	R_1 = 0.0389, wR_2 = 0.0906	R_1 = 0.0431, wR_2 = 0.1068	R_1 = 0.0279, wR_2 = 0.0724
Final R indexes [all data]	R_1 = 0.0620, wR_2 = 0.1076	R_1 = 0.0681, wR_2 = 0.1278	R_1 = 0.0295, wR_2 = 0.0735
Largest diff. peak/hole / e Å^{-3}	0.41/-0.46	0.43/-0.65	0.35/-0.29

Table S1 (continued). Crystal data and structure refinement for [(Tp^{Ph,Me})Zn(**MBI**)] complexes.

Compound	**[(Tp^{Ph,Me})Zn(14)]**	**[(Tp^{Ph,Me})Zn(15)]**	**[(Tp^{Ph,Me})Zn(16)]**
Identification code	2117768	2117769	2117774
Empirical formula	$C_{39}H_{35}BN_8O_2Zn$	$C_{38}H_{34}BN_9O_2Zn$	$C_{44.58}H_{43.62}BN_8O_4SZn$
Formula weight	723.93	724.92	863.69
Temperature/K	100	100	100
Crystal system	orthorhombic	monoclinic	monoclinic
Space group	Pbca	C2/c	P21/c
a/Å	17.7615(5)	24.7054(13)	24.9170(10)
b/Å	17.8877(6)	12.3535(7)	12.6257(7)
c/Å	21.4370(7)	25.1484(14)	27.9346(11)
α/°	90	90	90
β/°	90	107.2460(10)	100.3670(10)
γ/°	90	90	90
Volume/Å^3	6810.8(4)	7330.2(7)	8644.6(7)
Z	8	8	8
ρ_{calc}g/cm^3	1.412	1.314	1.327
μ/mm^{-1}	0.77	0.717	0.669
F(000)	3008	3008	3601
Crystal size/mm^3	0.1 × 0.1 × 0.1	0.1 × 0.1 × 0.1	0.1 × 0.1 × 0.1
Radiation	MoKα (λ = 0.71073)	MoKα (λ = 0.71073)	MoKα (λ = 0.71073)
2Θ range for data collection/°	3.748 to 52.77	3.392 to 52.828	4.032 to 52.122
Index ranges	-22 ≤ h ≤ 13, -17 ≤ k ≤ 22, -26 ≤ l ≤ 19	-30 ≤ h ≤ 30, -15 ≤ k ≤ 15, -31 ≤ l ≤ 29	-30 ≤ h ≤ 30, -15 ≤ k ≤ 15, -34 ≤ l ≤ 34
Reflections collected	29933	45312	170610
Independent reflections	6969 [R_{int} = 0.0536, R_{sigma} = 0.0430]	7531 [R_{int} = 0.0526, R_{sigma} = 0.0323]	17087 [R_{int} = 0.0922, R_{sigma} = 0.0448]
Data/restraints/parameters	6969/0/464	7531/84/434	17087/0/1109
Goodness-of-fit on F^2	1.045	1.037	1.023
Final R indexes [I>=2σ (I)]	R_1 = 0.0314, wR_2 = 0.0807	R_1 = 0.0393, wR_2 = 0.0915	R_1 = 0.0449, wR_2 = 0.0958
Final R indexes [all data]	R_1 = 0.0377, wR_2 = 0.0848	R_1 = 0.0480, wR_2 = 0.0971	R_1 = 0.0717, wR_2 = 0.1089
Largest diff. peak/hole / e Å^{-3}	0.36/-0.45	1.01/-0.84	0.98/-0.71

Table S1 (continued). Crystal data and structure refinement for [($Tp^{Ph,Me}$)Zn(**MBI**)] complexes.

Compound	**[($Tp^{Ph,Me}$)Zn(19)]**	**[($Tp^{Ph,Me}$)Zn(23)]**	**[($Tp^{Ph,Me}$)Zn(24)]**
Identification code	2117772	2117777	2117771
Empirical formula	$C_{44}H_{42}BN_{10}O_2Zn$	$C_{40}H_{37}BN_8O_{2.29}Zn$	$C_{39}H_{36}BN_9O_2Zn$
Formula weight	819.05	742.63	738.95
Temperature/K	100	100	100
Crystal system	triclinic	monoclinic	triclinic
Space group	P-1	P21/c	P-1
a/Å	12.4046(17)	12.7201(5)	11.4528(7)
b/Å	12.5836(17)	22.4184(9)	11.6697(6)
c/Å	14.653(2)	13.4325(5)	13.8501(7)
α/°	88.179(5)	90	80.236(2)
β/°	66.003(4)	110.4160(10)	88.814(2)
γ/°	74.862(4)	90	73.751(2)
Volume/$Å^3$	2009.8(5)	3589.9(2)	1750.69(17)
Z	2	4	2
ρ_{calc}g/cm^3	1.353	1.374	1.402
μ/mm^{-1}	0.663	0.733	0.752
F(000)	854	1545	768
Crystal size/mm^3	0.1 × 0.1 × 0.1	0.2 × 0.1 × 0.1	0.2 × 0.04 × 0.03
Radiation	MoKα (λ = 0.71073)	MoKα (λ = 0.71073)	MoKα (λ = 0.71073)
2Θ range for data collection/°	4.732 to 52.01	4.866 to 52.82	3.69 to 51.368
Index ranges	-15 ≤ h ≤ 15, -15 ≤ k ≤ 15, -18 ≤ l ≤ 18	-15 ≤ h ≤ 15, -26 ≤ k ≤ 28, -16 ≤ l ≤ 16	-13 ≤ h ≤ 13, -14 ≤ k ≤ 14, -16 ≤ l ≤ 16
Reflections collected	48102	55492	54561
Independent reflections	7869 [R_{int} = 0.0683, R_{sigma} = 0.0402]	7360 [R_{int} = 0.0628, R_{sigma} = 0.0342]	6643 [R_{int} = 0.0783, R_{sigma} = 0.0392]
Data/restraints/parameters	7869/0/527	7360/0/486	6643/0/475
Goodness-of-fit on F^2	1.046	1.084	1.034
Final R indexes [I>=2σ (I)]	R_1 = 0.0314, wR_2 = 0.0810	R_1 = 0.0462, wR_2 = 0.0941	R_1 = 0.0354, wR_2 = 0.0725
Final R indexes [all data]	R_1 = 0.0396, wR_2 = 0.0854	R_1 = 0.0611, wR_2 = 0.1011	R_1 = 0.0503, wR_2 = 0.0803
Largest diff. peak/hole / e $Å^{-3}$	0.31/-0.35	0.97/-0.39	0.33/-0.33

8. Calculated physicochemical properties

Table S2. Calculated physicochemical properties of salicylic acid and salicylic acid MBIs. Calculator plugins were used for physicochemical property prediction and calculation, Marvin 21.11.2021, ChemAxon (http://www.chemaxon.com).

Compound	pK_a	logP	log$D_{7.4}$
**1**	2.79, 13.23	1.98	-1.52
**2**	3.55, 9.62	0.00	-3.34
**3**	2.96, 9.44	0.99	-2.49
**4**	2.00, 5.87, 12.64	0.92	-1.40
**5**	3.43, 9.24	0.96	–2.45
**6**	9.72	2.32	2.32
**7**	8.03, 13.54	1.55	1.45
**8**	5.72, 8.66	0.95	-0.66
**9**	9.82, 12.83	0.67	0.67
**10**	1.06, 7.32	2.12	1.78
**11**	1.79, 8.79	3.83	3.82
**12**	7.22	0.35	0.02
**13**	8.96	1.34	1.32
**14**	4.40, 10.45	1.26	1.26
**15**	8.32	1.30	0.82
**16**	4.99, 10.41	-0.43	-1.36
**17**	8.98, 12.01	0.56	0.55
**18**	4.79, 10.26, 13.18	0.48	0.48
**19**	8.48, 10.86	0.53	0.03
**20**	5.82, 7.24	-0.37	-2.02
**21**	5.72, 9.07	0.61	-1.00
**22**	4.73, 5.64, 11.81	0.54	-1.07
**23**	5.69, 11.69, 12.91	0.26	0.25
**24**	0.03, 9.01, 12.68	0.30	0.23
**25**	1.30, 7.71	0.96	0.81
**26**	0.70, 4.90, 11.85	1.69	1.69
**27**	7.88, 1.92	2.51	2.40
**28**	1.28, 4.98, 11.89	3.42	3.42

Table S3. Calculated physicochemical properties of MBI **11** derivatives. Calculator plugins were used for physicochemical property prediction and calculation, Marvin 21.11.2021, ChemAxon (http://www.chemaxon.com).

Compound	pK_a	logP	log$D_{7.4}$
**11a**	1.77, 8.69	5.48	5.46
**11b**	1.77, 8.69	6.00	5.97
**11c**	1.77, 8.67	6.09	6.06
**11d**	1.39, 9.12, 6.26	4.79	4.72
**11e**	1.53, 2.32, 9.06	3.72	3.71
**11f**	1.38, 5.86, 9.09	4.35	4.32
**11g**	1.77, 8.49	4.62	4.59

Table S4. Possible protein-ligand contacts for compounds **11** and **11a** upon docking with the active site of GLO1. Residue and atom labels can be found in Figure S2.

Inhibitor	Protein Residue – Atom of Ligand	Distance / Å	Type of Interaction
**11**	Phe67-L2	3.2	hydrophobic, π-π interaction
	Phe162-L5	2.9	hydrophobic, π-π interaction
	Zn-L7	2.3	metal-ligand
	Leu69-L8	3.3	hydrophobic
	Zn-L12	2.1	metal-ligand
	Met179-L14	3.1	hydrophobic
	Leu69-L16	3.4	hydrophobic
**11a**	Phe67-L2	3.2	hydrophobic, π-π interaction
	Phe162-L5	2.9	hydrophobic, π-π interaction
	Zn-L7	2.3	metal-ligand
	Leu69-L8	3.3	hydrophobic
	Zn-L12	2.1	metal-ligand
	Met179-L14	3.1	hydrophobic
	Leu69-L16	3.4	hydrophobic
	Phe62-L18	4.0	hydrophobic, π-π interaction
	Cys60-L18	4.1	hydrophobic
	Phe62-L20	4.3	hydrophobic, π-π interaction
	Leu182-L20	3.4	hydrophobic
	Phe62-L22	3.8	hydrophobic, π-π interaction

9. References

(1) Tipparaju, S. K.; Joyasawal, S.; Pieroni, M.; Kaiser, M.; Brun, R.; Kozikowski, A. P. In Pursuit of Natural Product Leads: Synthesis and Biological Evaluation of 2-[3-hydroxy-2-[(3-hydroxypyridine-2- carbonyl)amino]phenyl]benzoxazole- 4-carboxylic acid (A-33853) and Its Analogues: Discovery of N-(2-Benzoxazol-2-ylphenyl)benzamides as Novel Antileishmanial Chemotypes. *J. Med. Chem.* **2008**, *51*, 7344-7347.

(2) Noda, H.; Bode, J. W. Synthesis of Chemically and Configurationally Stable Monofluoro Acylboronates: Effect of Ligand Structure on their Formation, Properties, and Reactivities. *J. Am. Chem. Soc.* **2015**, *137*, 3958-3966.

(3) Kloeckner, U.; Nachtsheim, B. J. Mild hypervalent iodine mediated oxidative nitration of N-aryl sulfonamides. *Chem. Commun.* **2014**, *50*, 10485-10487.

(4) Chiba, T.; Ohwada, J.; Sakamoto, H.; Kobayashi, T.; Fukami, T. A.; Irie, M.; Miura, T.; Ohara, K.; Koyano, H. Design and evaluation of azaindole-substituted N-hydroxypyridones as glyoxalase I inhibitors. *Bioorg. Med. Chem. Lett.* **2012**, *22*, 7486-7489.

(5) Weimer, M.; Jiang, X.; Ponta, O.; Stanzel, S.; Freyberger, A.; Kopp-Schneider, A. The impact of data transformation on concentration-response modeling. *Toxicol. Lett.* **2012**, *213*, 292-298.

(6) GraphPad Software. Incomplete dose-response curves. https://www.graphpad.com/guides/prism/latest/curve-fitting/reg_incomplete_dose-respone_curves.htm (accessed Oct 21, 2021).

(7) Sheldrick G. Crystal structure refinement with SHELXL *Acta Crystallogr., Sect. C: Struct. Chem.*, **2015**, *71*, 3-8.

(8) Sheldrick G. A short history of SHELX. *Acta Crystallogr. Sect. A: Found. Crystallogr.*, **2008**, 64, 112-122.

(9) Dolomanov O. V.; Bourhis L. J.; Gildea R. J.; Howard J. A. K; Puschmann H. OLEX2: a complete structure solution, refinement and analysis program *J. Appl. Crystallogr.*, **2009**, 42, 339-341.

(10) Spek A. L. *PLATON* SQUEEZE: a tool for the calculation of the disordered solvent contribution to the calculated structure factors. *Acta Crystallogr. Sect. C: Cryst. Struct. Commun.*, **2015**, 71, 9-18.

10. NMR Spectra

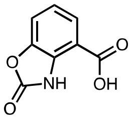

3

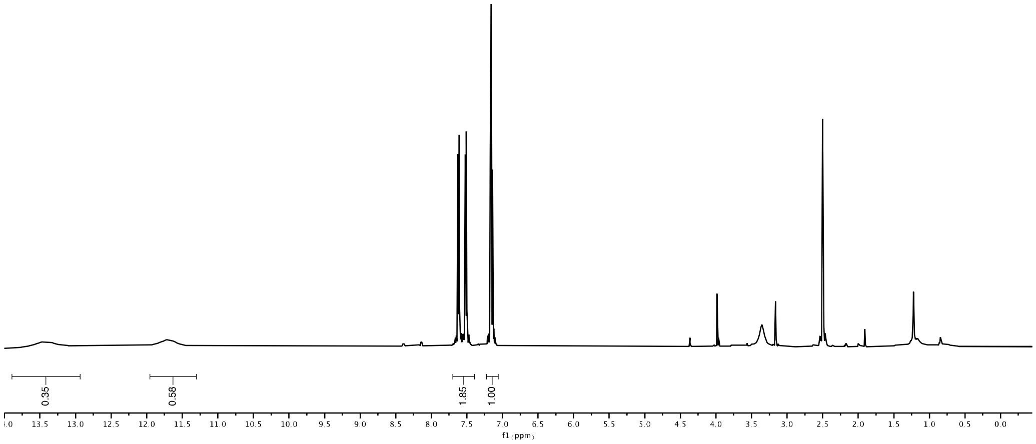

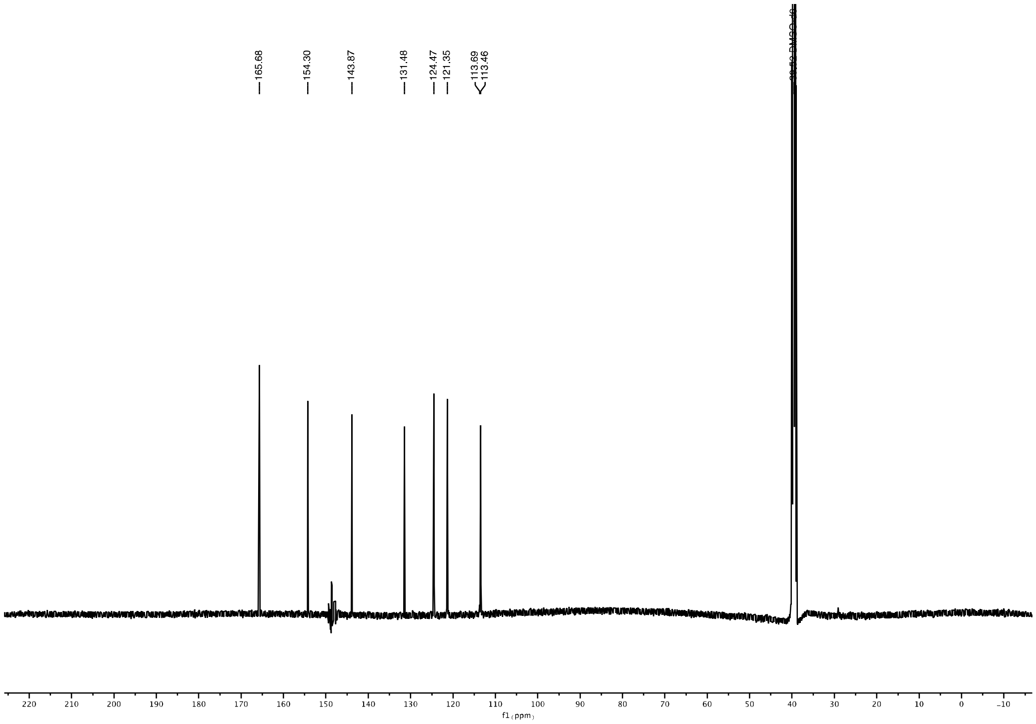

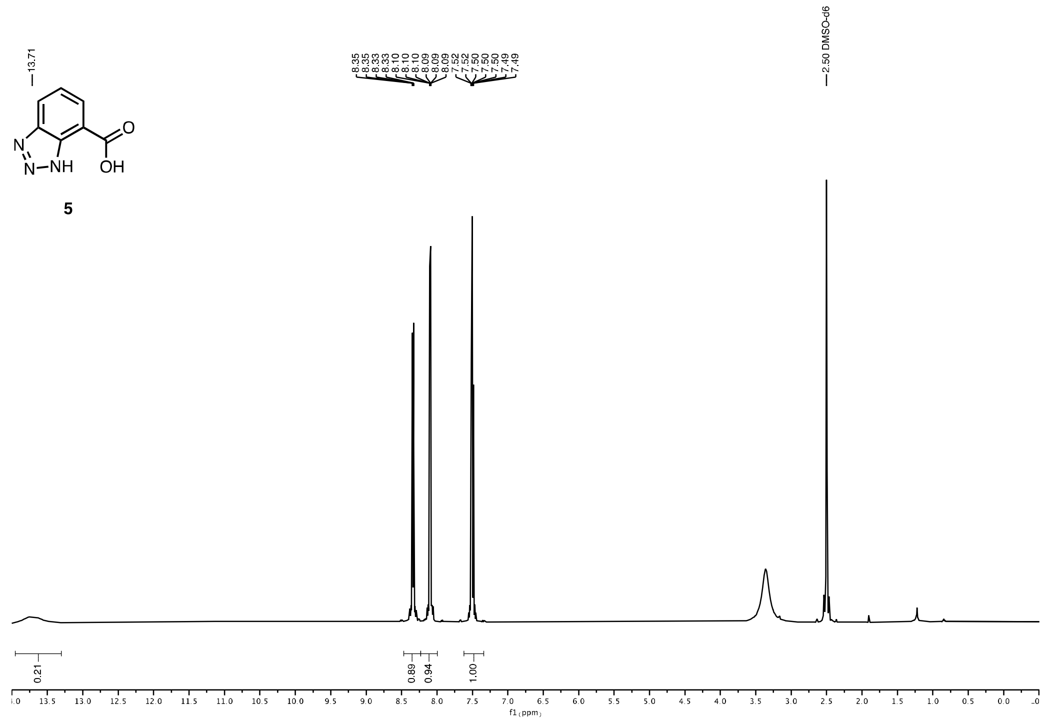
13.71
8.35
8.35
8.33
8.33
8.10
8.10
8.09
8.09
7.52
7.52
7.50
7.50
7.49
7.49
2.50 DMSO-d6
O
N
N
NH
OH
5
0.21
0.89
0.94
1.00
f1 (ppm)

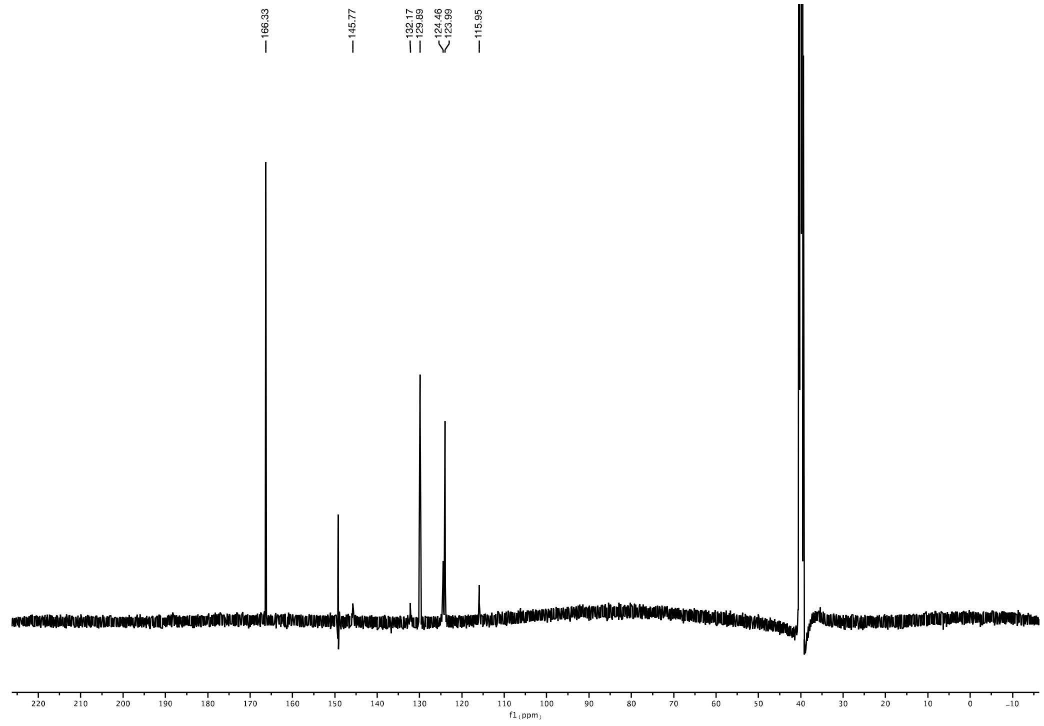
166.33
145.77
132.17
129.89
124.46
123.99
115.95
f1 (ppm)

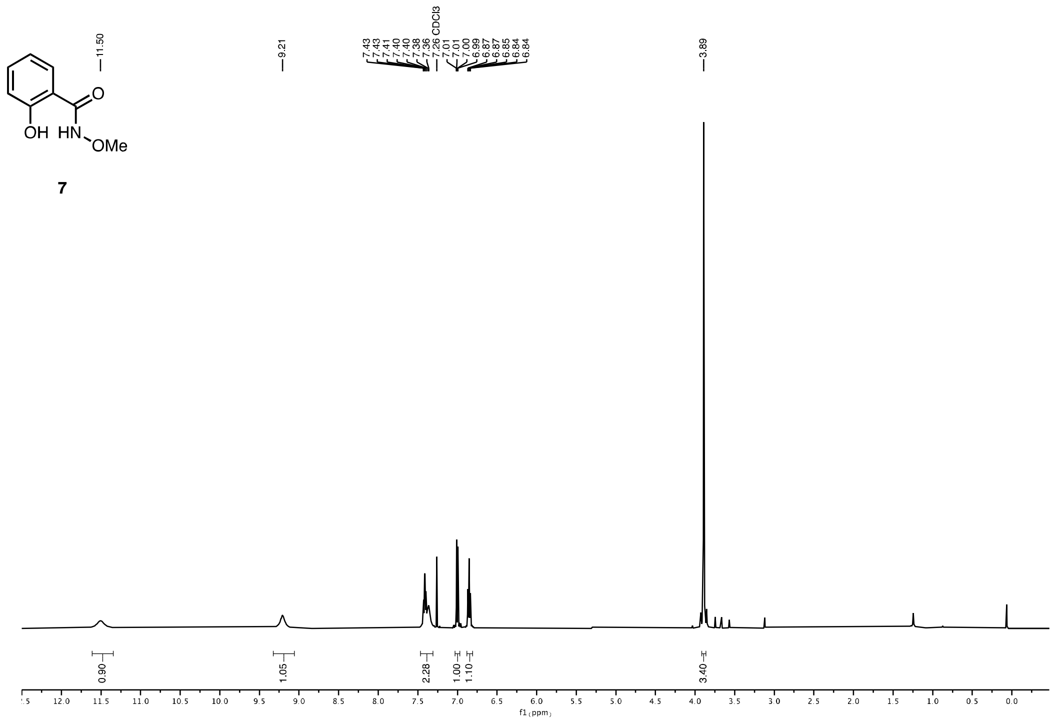
OH HN OMe
O
11.50
9.21
7.43 7.43 7.41 7.40 7.40 7.38 7.36 7.26 CDCl3 7.01 7.01 7.00 6.99 6.87 6.85 6.84 6.84
3.89
0.90
1.05
2.28
1.00
1.10
3.40
f1 (ppm)

7

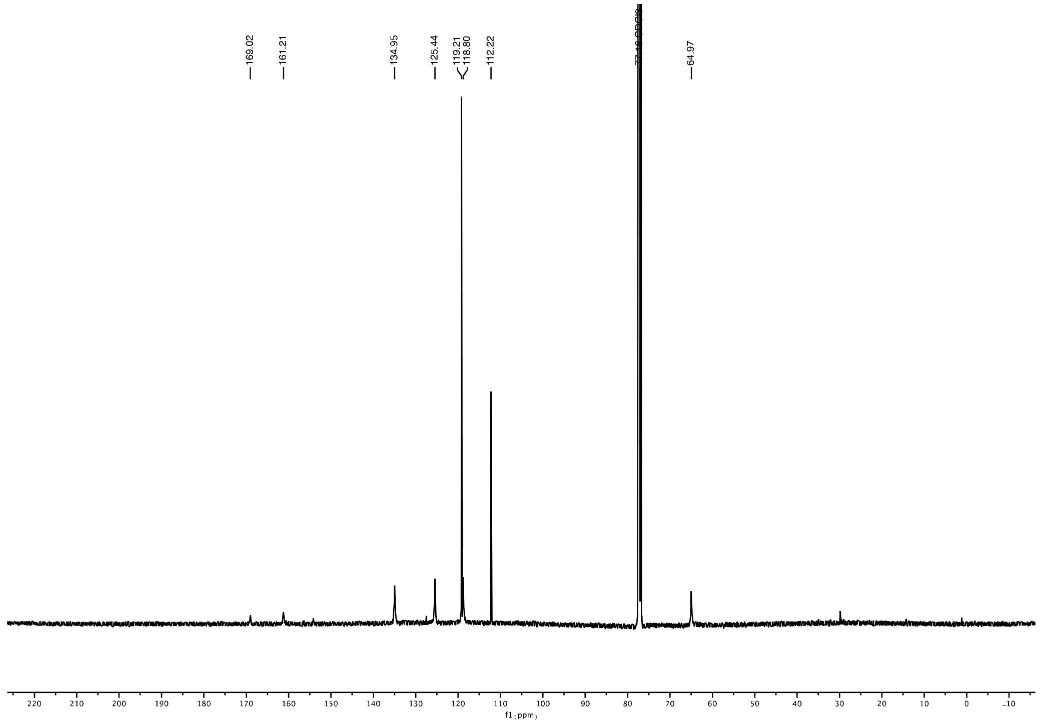
169.02
161.21
134.95
125.44
119.21
118.80
112.22
77.16 CDCl3
64.97
f1 (ppm)

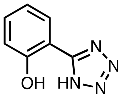

8

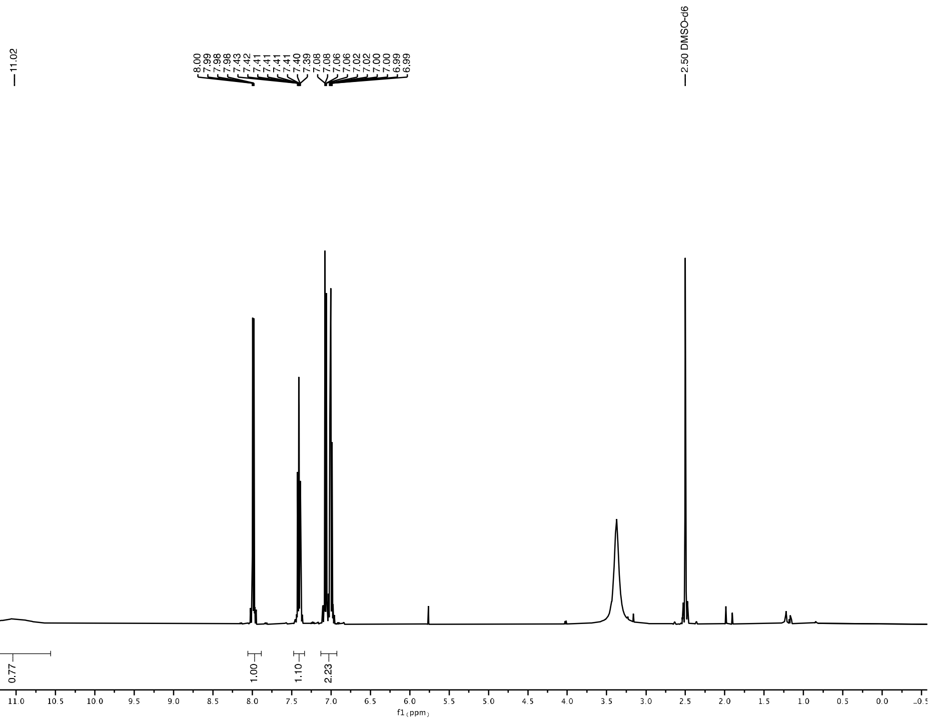

11.02
8.00
7.99
7.98
7.98
7.43
7.42
7.41
7.41
7.41
7.40
7.39
7.08
7.08
7.06
7.06
7.02
7.02
7.00
7.00
6.99
6.99
2.50 DMSO-d6
0.77
1.00
1.10
2.23
f1 (ppm)

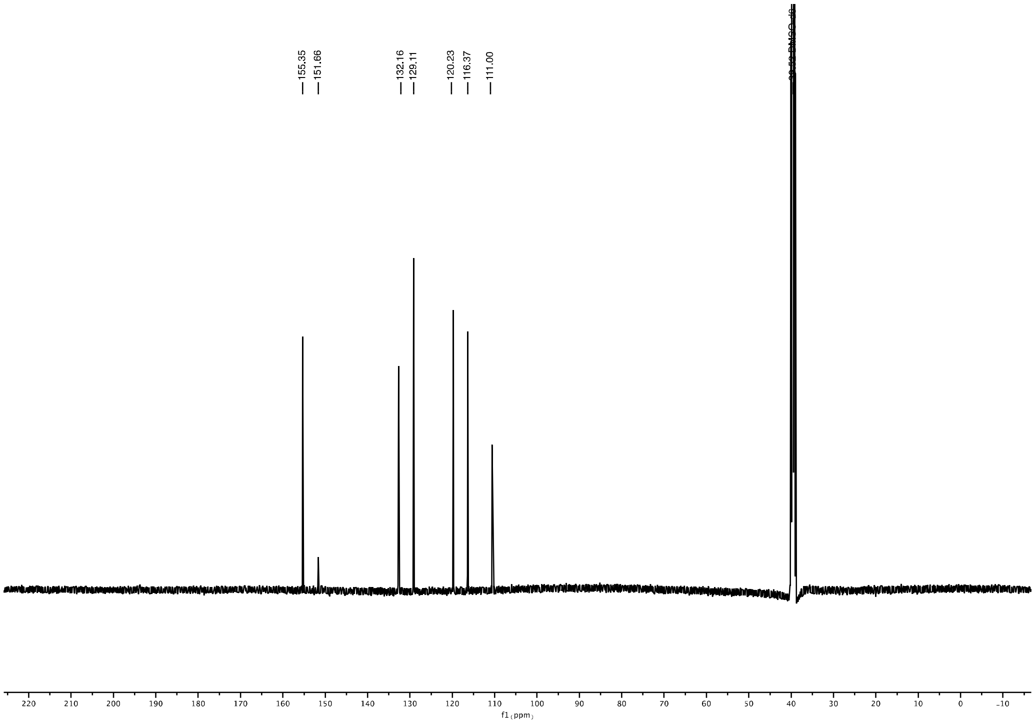

155.35
151.66
132.16
129.11
120.23
116.37
111.00
f1 (ppm)

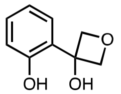

9

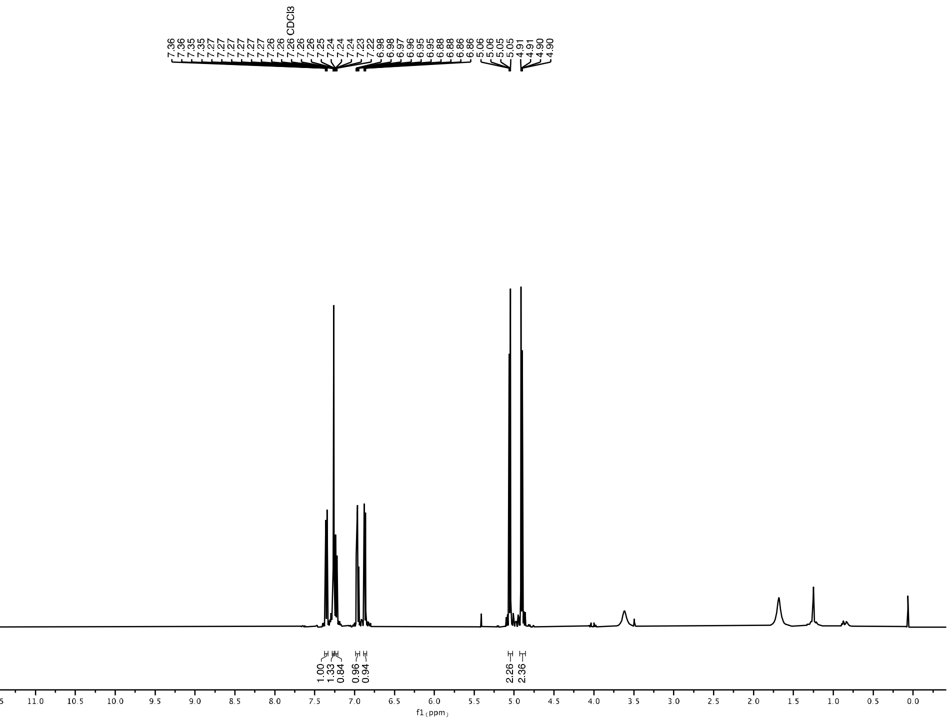
7.36
7.36
7.35
7.35
7.27
7.27
7.27
7.27
7.27
7.27
7.26
7.26 CDCl3
7.26
7.26
7.25
7.24
7.24
7.24
7.23
7.22
6.98
6.98
6.97
6.96
6.95
6.95
6.88
6.88
6.86
6.86
5.06
5.06
5.05
5.05
4.91
4.91
4.90
4.90
1.00
1.33
0.84
0.96
0.94
2.26
2.36
12.0
11.5
11.0
10.5
10.0
9.5
9.0
8.5
8.0
7.5
7.0
6.5
6.0
5.5
5.0
4.5
4.0
3.5
3.0
2.5
2.0
1.5
1.0
0.5
0.0
f1 (ppm)

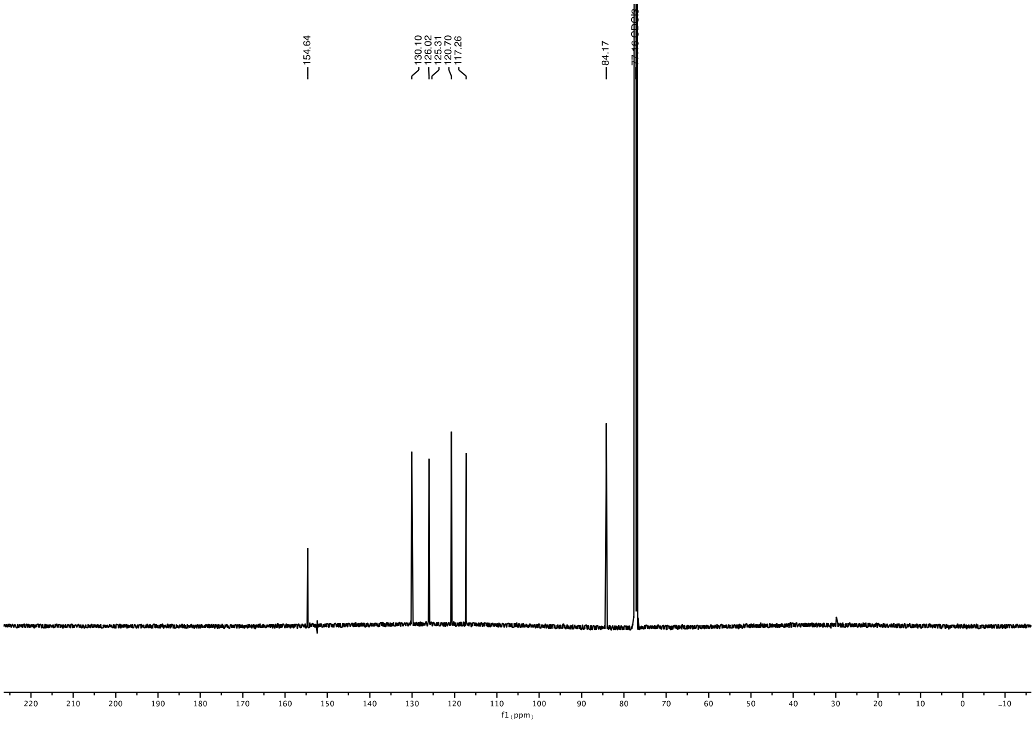
154.64
130.10
126.02
125.31
120.70
117.26
84.17
220
210
200
190
180
170
160
150
140
130
120
110
100
90
80
70
60
50
40
30
20
10
0
-10
f1 (ppm)

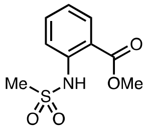

Me
NH
O
OMe
S
O
O

12

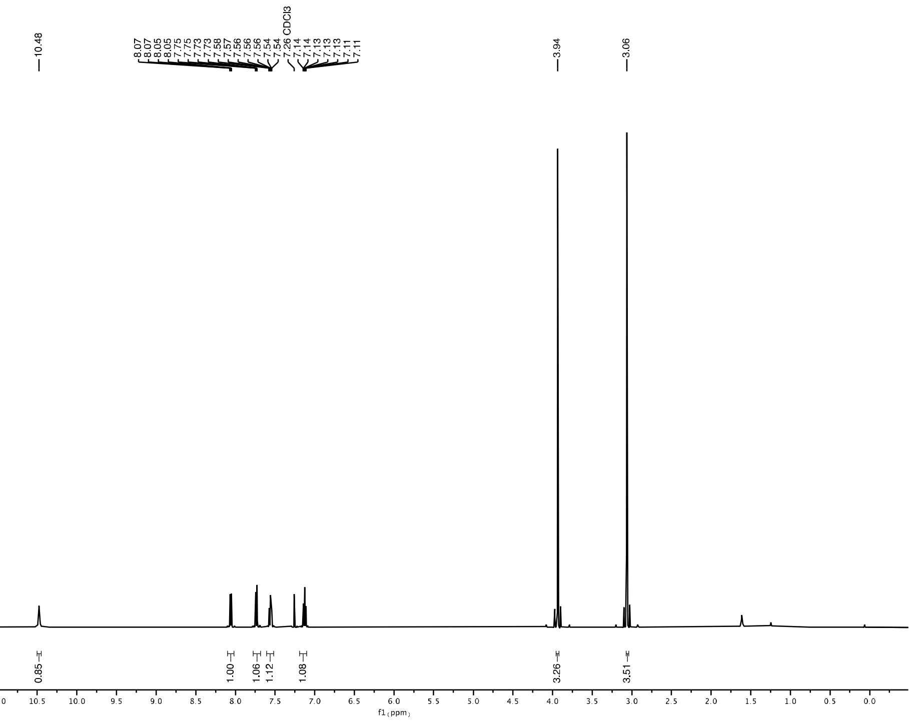

10.48
8.07
8.07
8.05
8.05
7.75
7.75
7.73
7.73
7.58
7.57
7.56
7.56
7.54
7.54
7.26 CDCl3
7.14
7.14
7.13
7.13
7.11
7.11
3.94
3.06
0.85
1.00
1.06
1.12
1.08
3.26
3.51
12.0 11.5 11.0 10.5 10.0 9.5 9.0 8.5 8.0 7.5 7.0 6.5 6.0 5.5 5.0 4.5 4.0 3.5 3.0 2.5 2.0 1.5 1.0 0.5 0.0
f1 (ppm)

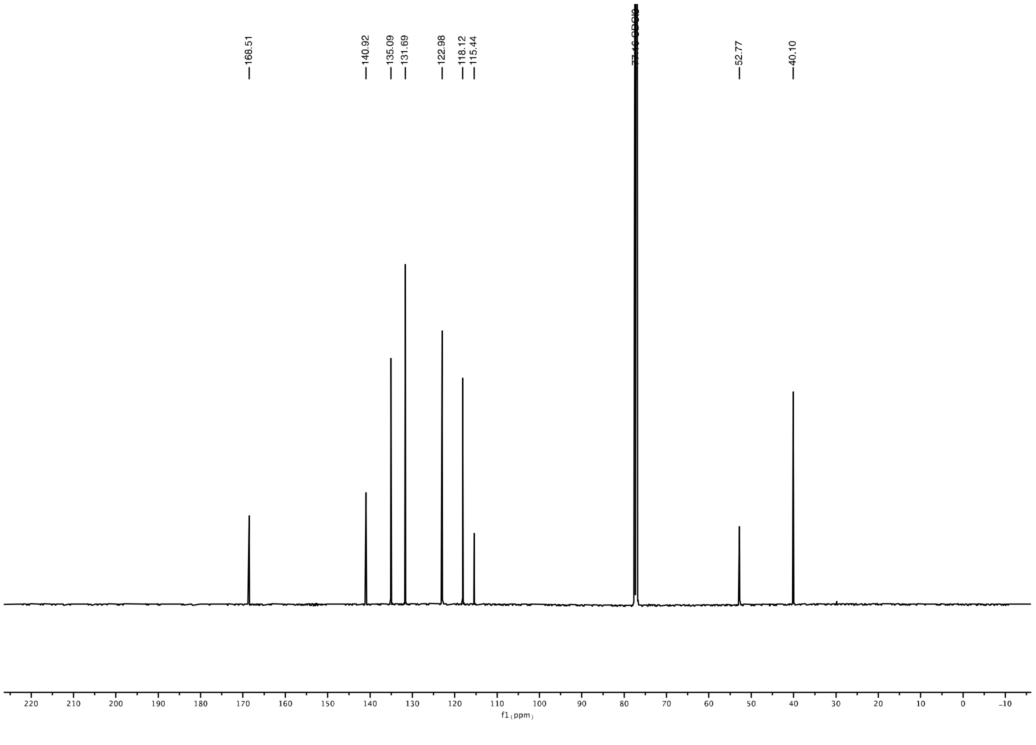

168.51
140.92
135.09
131.69
122.98
118.12
115.44
52.77
40.10
220 210 200 190 180 170 160 150 140 130 120 110 100 90 80 70 60 50 40 30 20 10 0 -10
f1 (ppm)

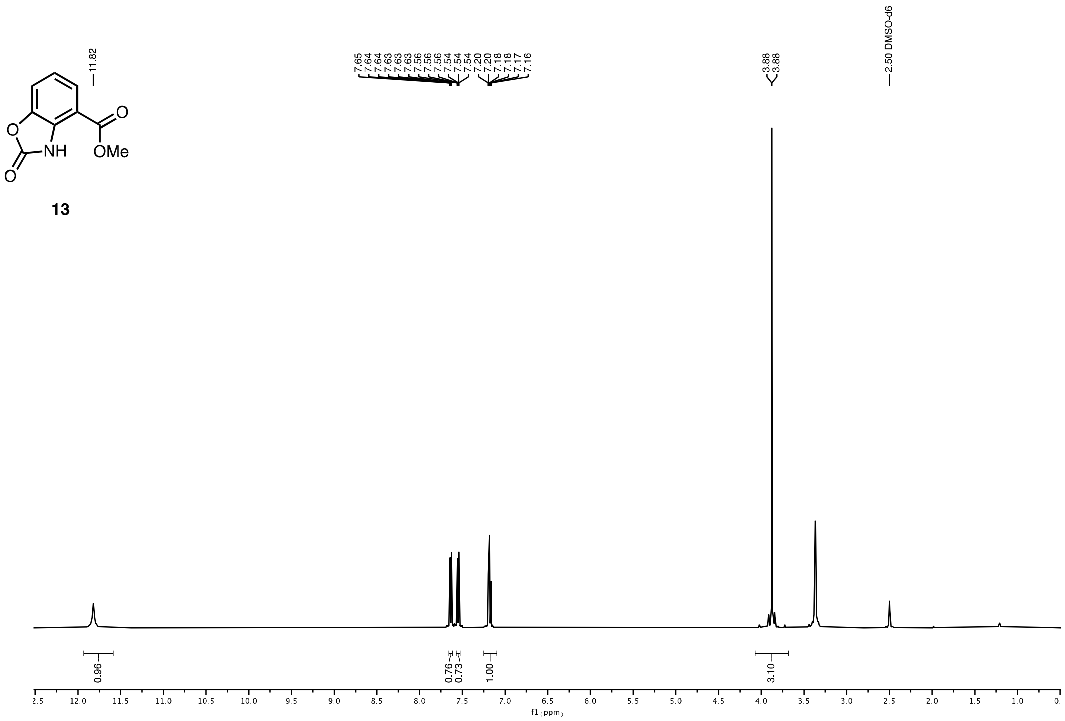
O
NH
O
O
OMe
13
11.82
7.65
7.64
7.64
7.63
7.63
7.63
7.56
7.56
7.54
7.54
7.54
7.20
7.20
7.18
7.18
7.17
7.16
3.88
3.88
2.50 DMSO-d6
0.96
0.76
0.73
1.00
3.10
f1 (ppm)

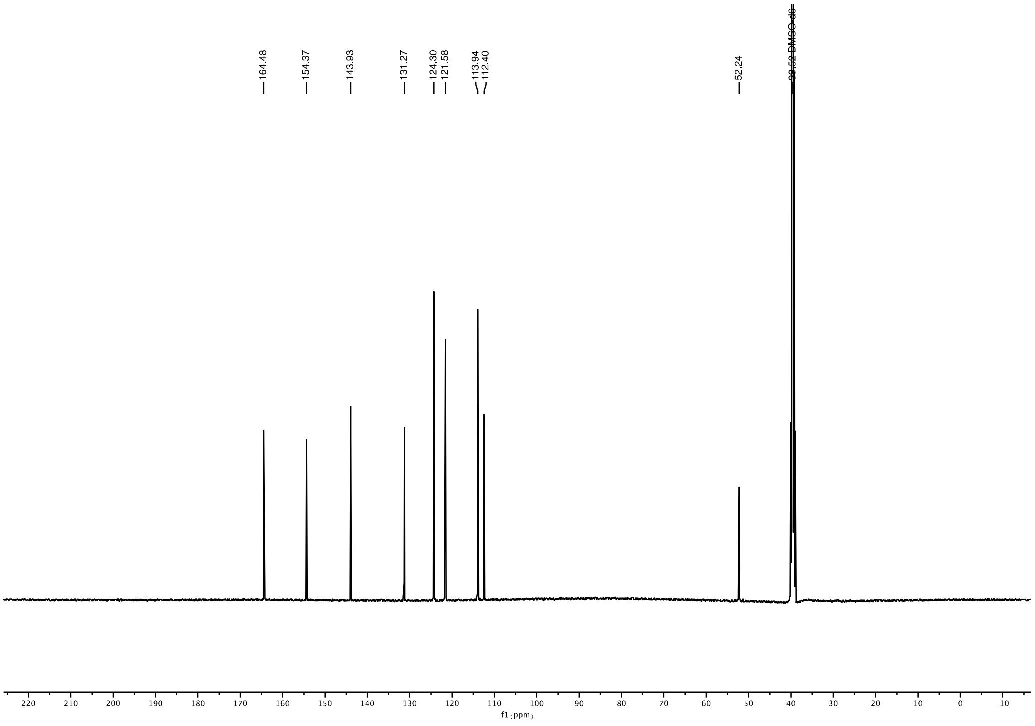
164.48
154.37
143.93
131.27
124.30
121.58
113.94
112.40
52.24
39.52 DMSO-d6
220
210
200
190
180
170
160
150
140
130
120
110
100
90
80
70
60
50
40
30
20
10
0
-10
f1 (ppm)

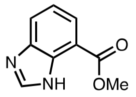

14

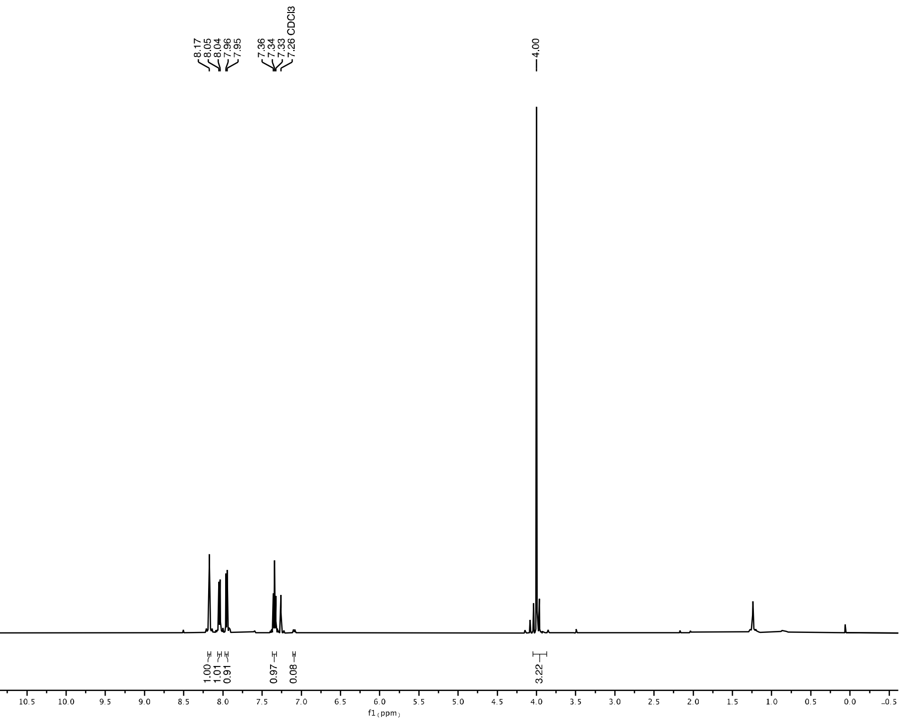
8.17
8.05
8.04
7.96
7.95
7.36
7.34
7.33
7.26 CDCl3
4.00
1.00
1.01
0.91
0.97
0.08
3.22
f1 (ppm)

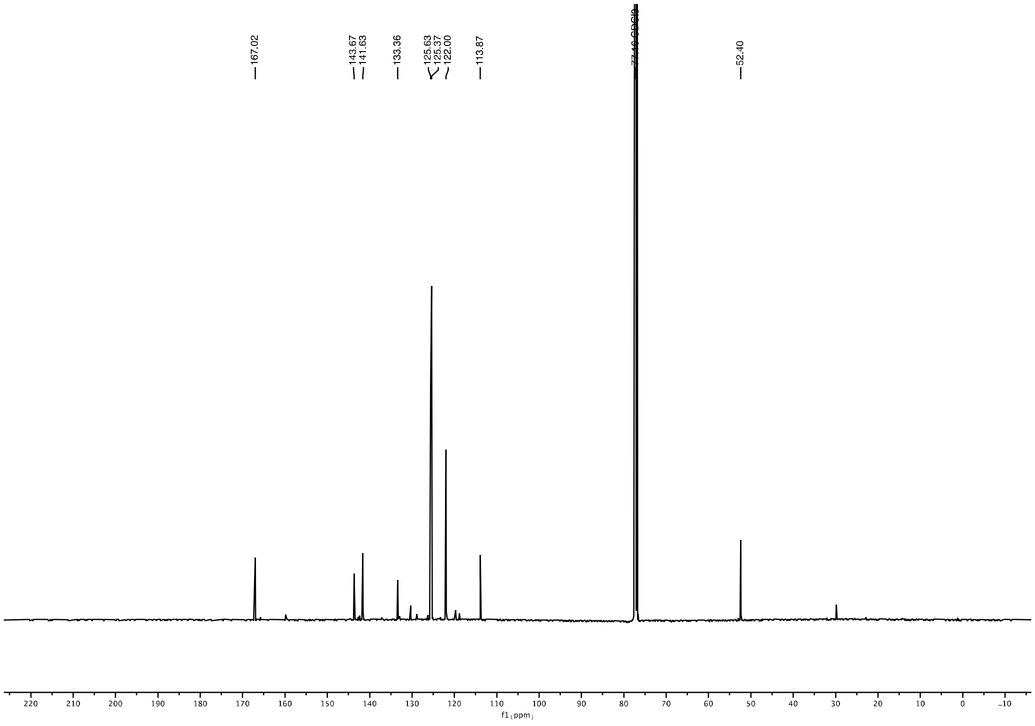
167.02
143.67
141.63
133.36
125.63
125.37
122.00
113.87
52.40
f1 (ppm)

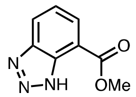

15

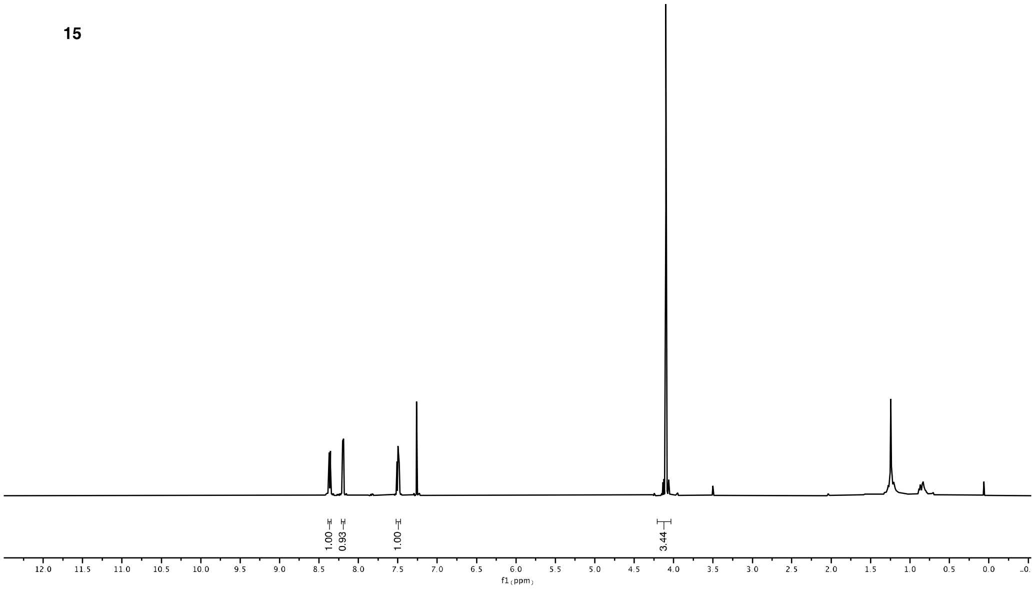

8.37
8.37
8.36
8.35
8.20
8.20
8.19
8.18
7.51
7.50
7.49
7.48
7.26 CDCl3
4.10
1.00
0.93
1.00
3.44
f1 (ppm)

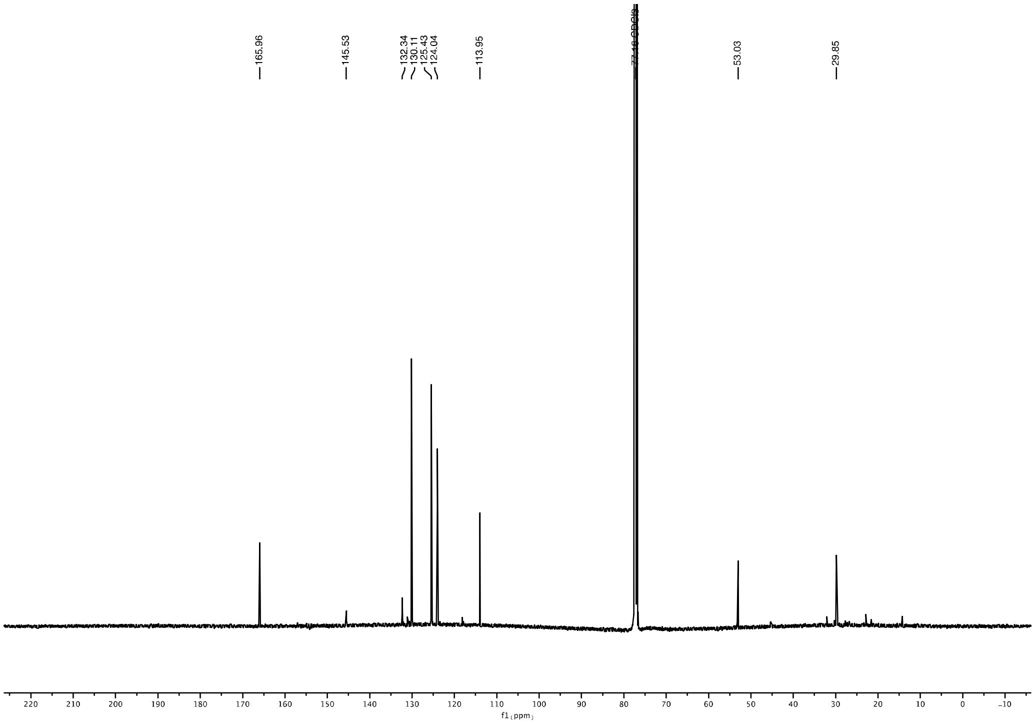

165.96
145.53
132.34
130.11
125.43
124.04
113.95
77.16 CDCl3
53.03
29.85
f1 (ppm)

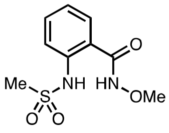
O
Me
S
NH
HN
OMe
O O

16

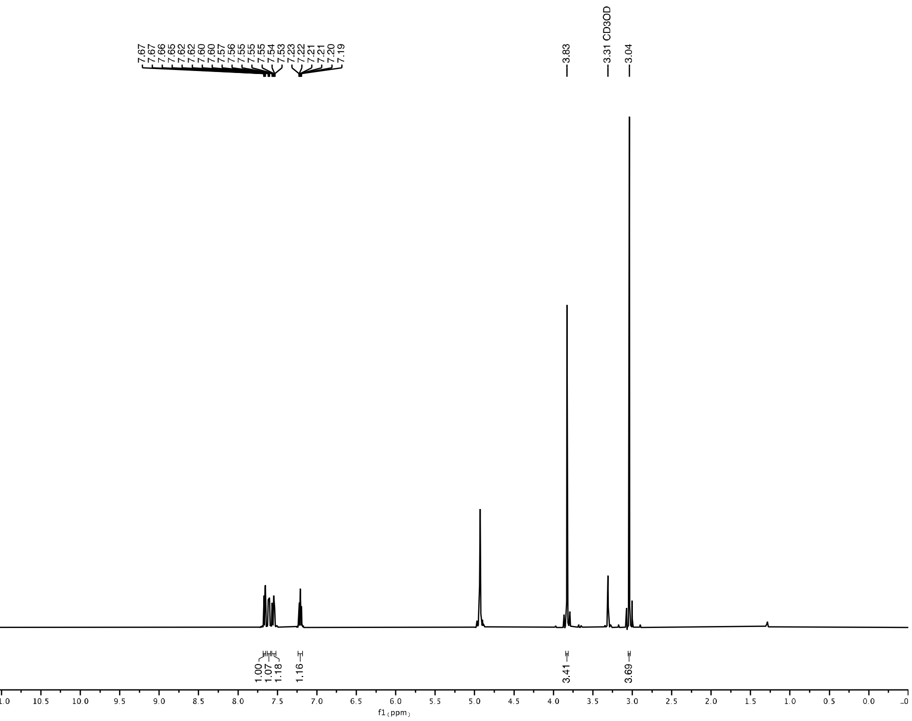
7.67
7.67
7.66
7.65
7.62
7.62
7.60
7.60
7.57
7.56
7.55
7.55
7.54
7.53
7.23
7.22
7.21
7.21
7.20
7.19
3.83
3.31 CD3OD
3.04
1.00
1.07
1.18
1.16
3.41
3.69
f1 (ppm)

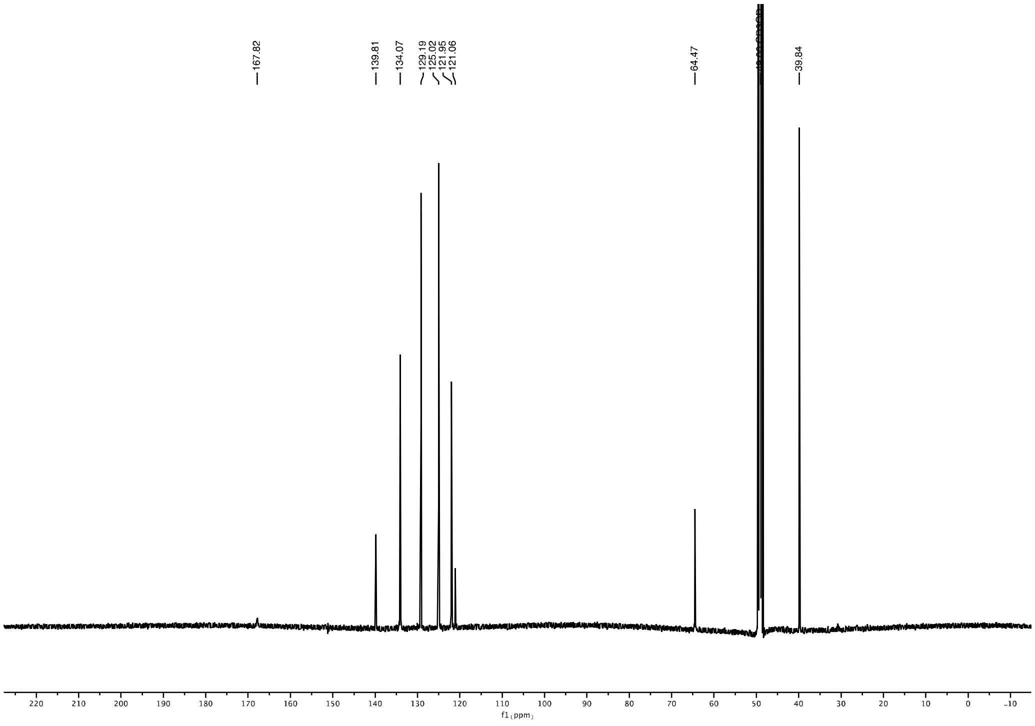
167.82
139.81
134.07
129.19
125.02
121.95
121.06
64.47
39.84
f1 (ppm)

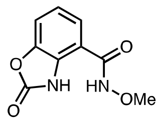

17

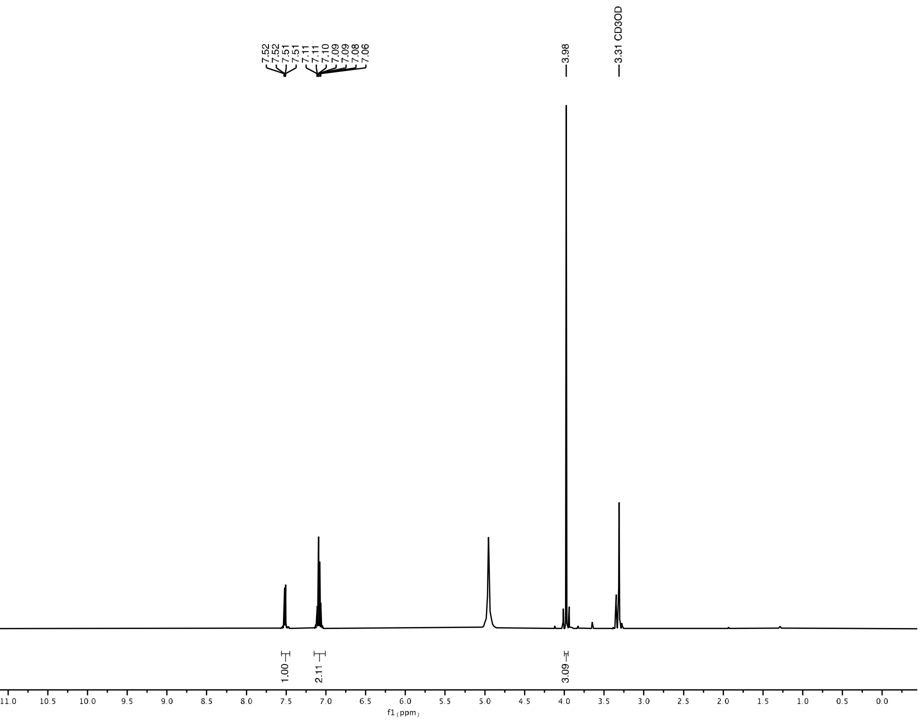

7.52
7.52
7.51
7.11
7.10
7.09
7.09
7.08
7.06
3.98
3.31 CD3OD
1.00
2.11
3.09
f1 (ppm)

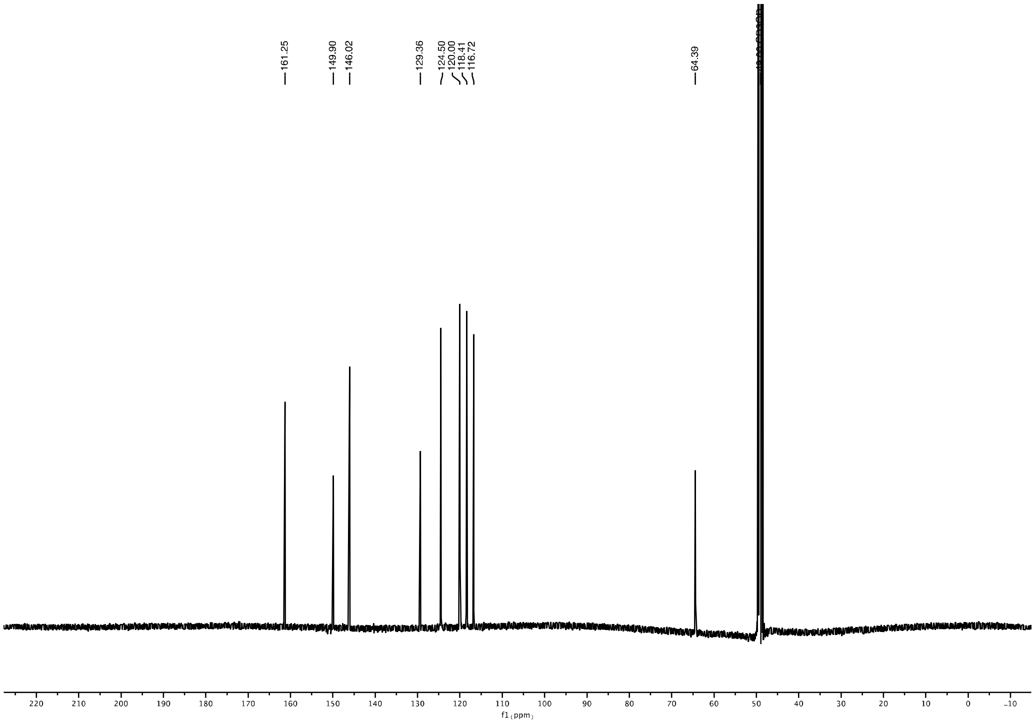

161.25
149.90
146.02
129.36
124.50
120.00
118.41
116.72
64.39
f1 (ppm)

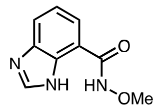

18

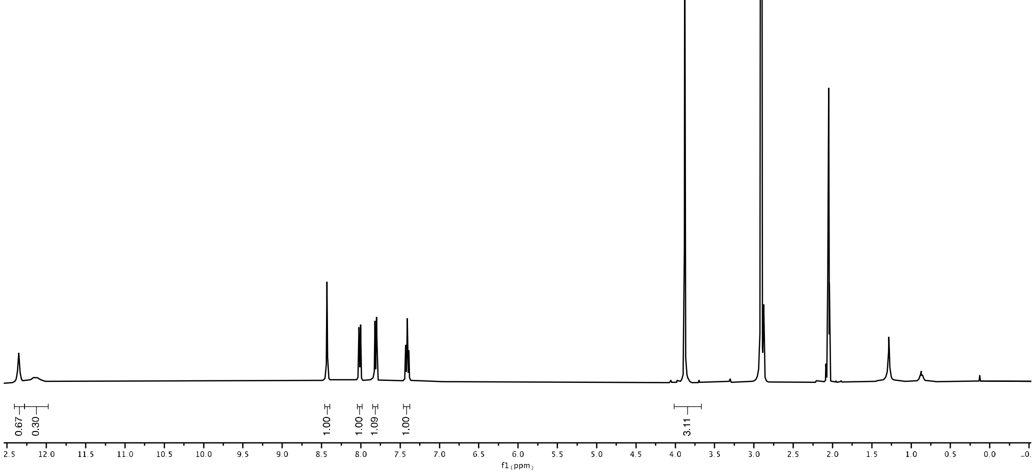
12.35
12.14
8.43
8.02
8.00
7.82
7.82
7.80
7.80
7.43
7.41
7.39
3.88
2.05 (CD3)2CO
0.67
0.30
1.00
1.00
1.09
1.00
3.11
f1 (ppm)

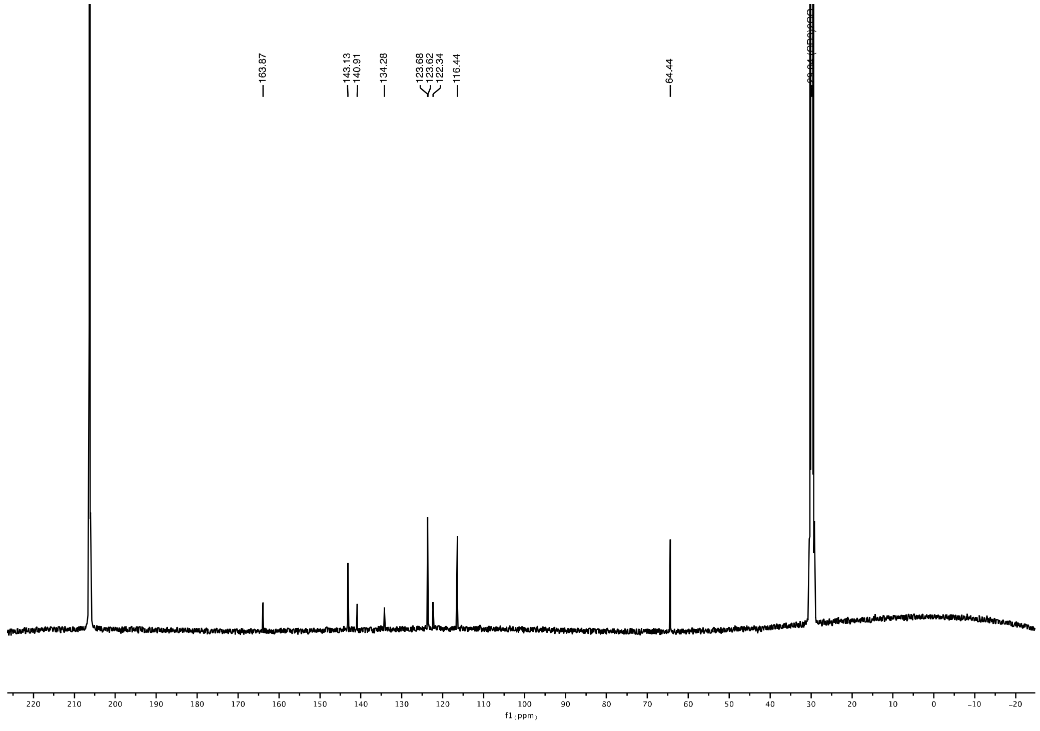
163.87
143.13
140.91
134.28
123.68
123.62
122.34
116.44
64.44
f1 (ppm)

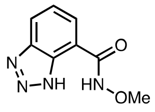

19

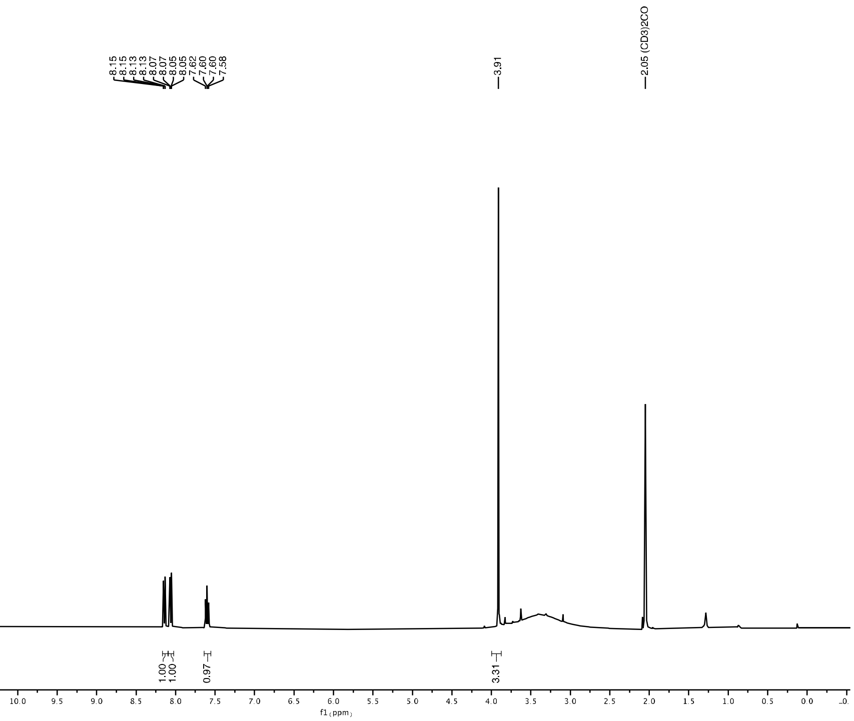

8.15
8.15
8.13
8.13
8.07
8.07
8.05
8.05
7.62
7.60
7.60
7.58
3.91
2.05 (CD3)2CO
1.00
1.00
0.97
3.31
f1 (ppm)

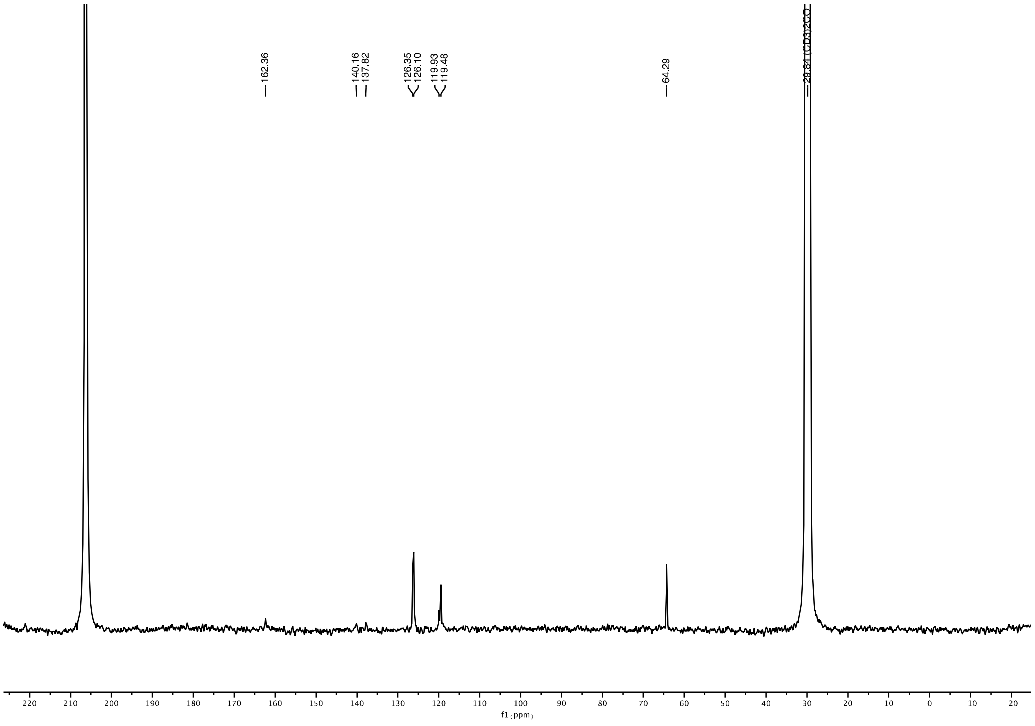

162.36
140.16
137.82
126.35
126.10
119.93
119.48
64.29
29.84 (CD3)2CO
f1 (ppm)

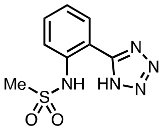
Me
NH
S
O O
HN
N
N
N

20

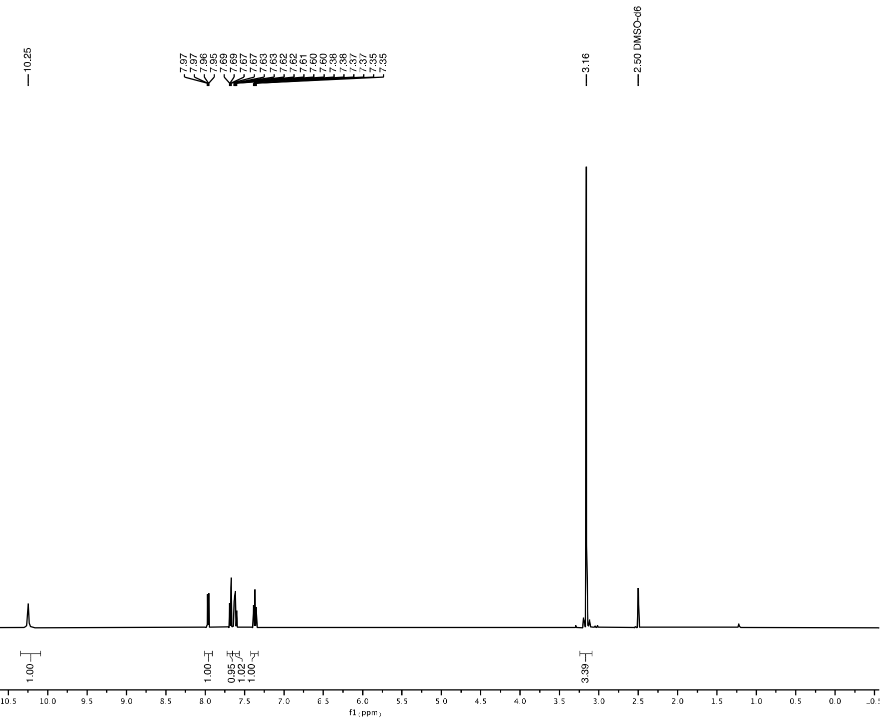
10.25
7.97
7.97
7.96
7.95
7.69
7.69
7.67
7.67
7.63
7.63
7.62
7.62
7.61
7.60
7.60
7.38
7.38
7.37
7.37
7.35
7.35
3.16
2.50 DMSO-d6
1.00
1.00
0.95
1.02
1.00
3.39
12.0 11.5 11.0 10.5 10.0 9.5 9.0 8.5 8.0 7.5 7.0 6.5 6.0 5.5 5.0 4.5 4.0 3.5 3.0 2.5 2.0 1.5 1.0 0.5 0.0
f1 (ppm)

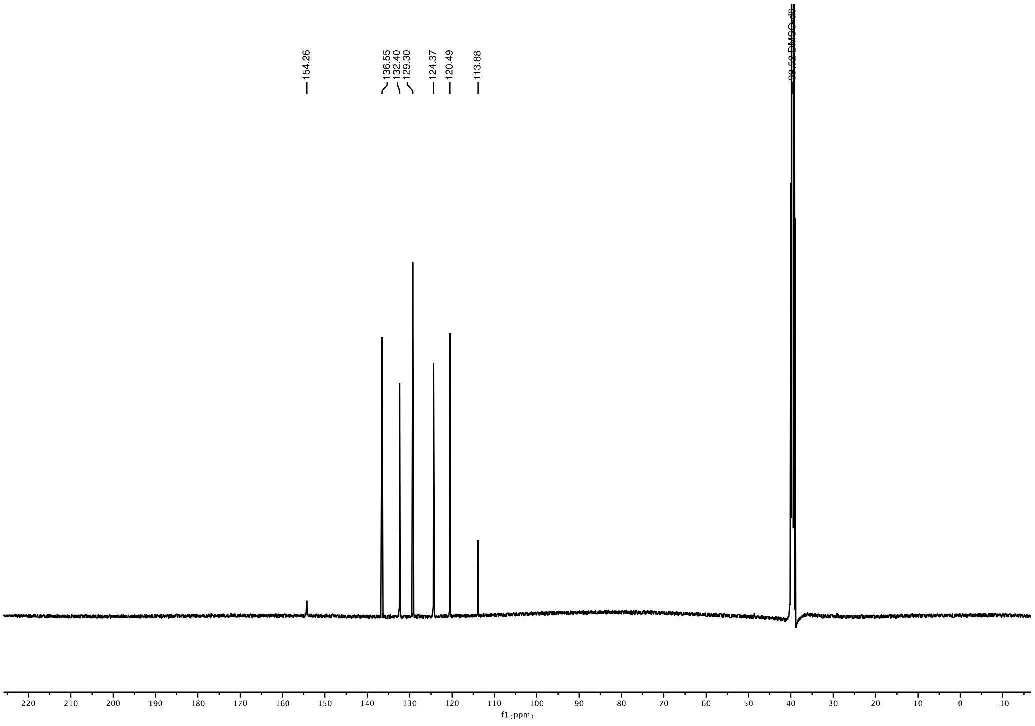
154.26
136.55
132.40
129.30
124.37
120.49
113.88
220 210 200 190 180 170 160 150 140 130 120 110 100 90 80 70 60 50 40 30 20 10 0 -10
f1 (ppm)

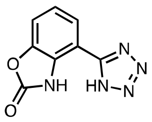
O
NH
HN
N
N
N
O

21

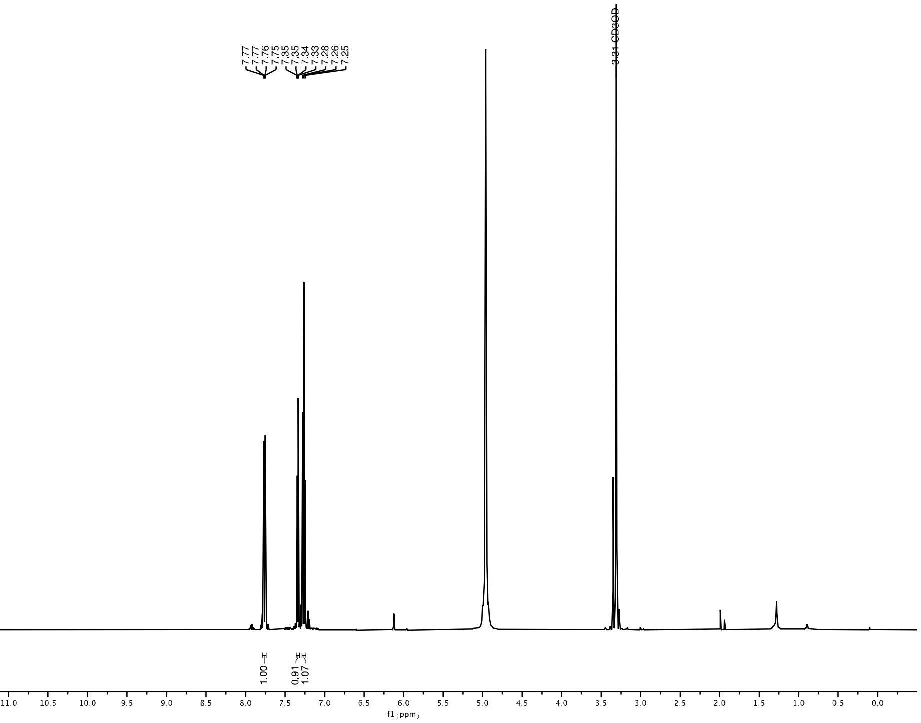
7.77
7.77
7.76
7.75
7.35
7.35
7.34
7.33
7.28
7.26
7.25
3.31 CD3OD
1.00
0.91
1.07
f1 (ppm)

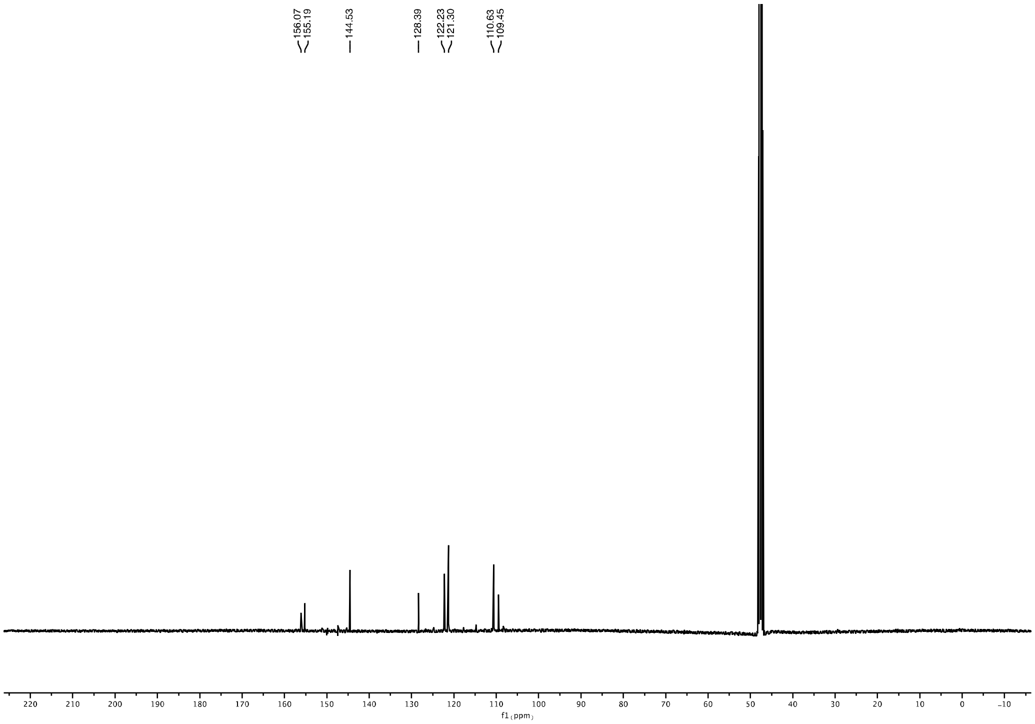
156.07
155.19
144.53
128.39
122.23
121.30
110.63
109.45
f1 (ppm)

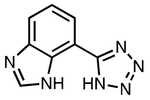
N
NH
HN
N
N
N

22

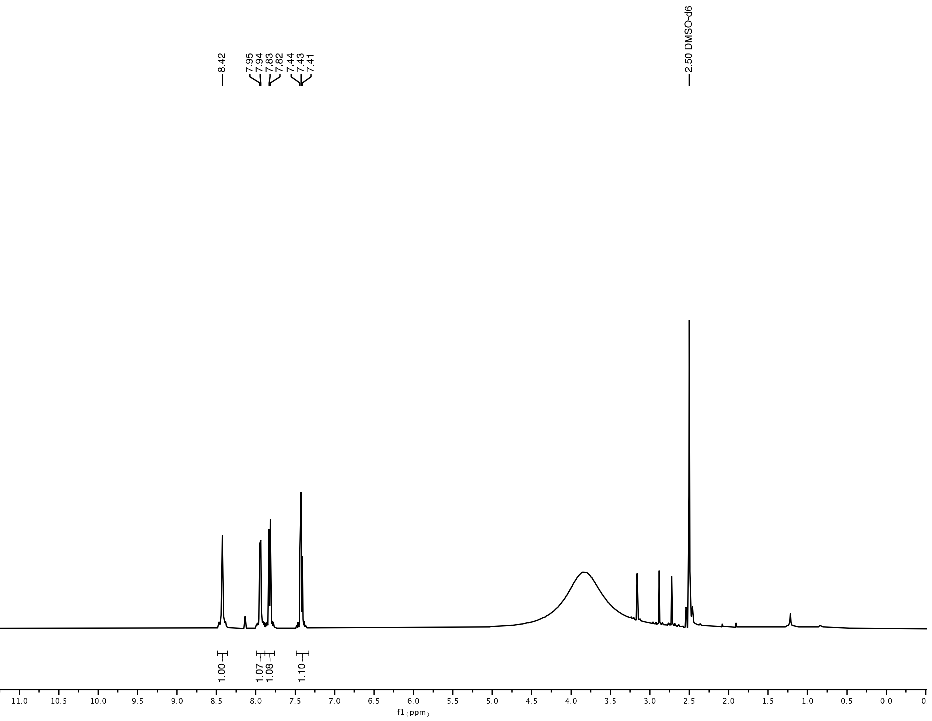
8.42
7.95
7.94
7.83
7.82
7.44
7.43
7.41
2.50 DMSO-d6
1.00
1.07
1.08
1.10
f1 (ppm)

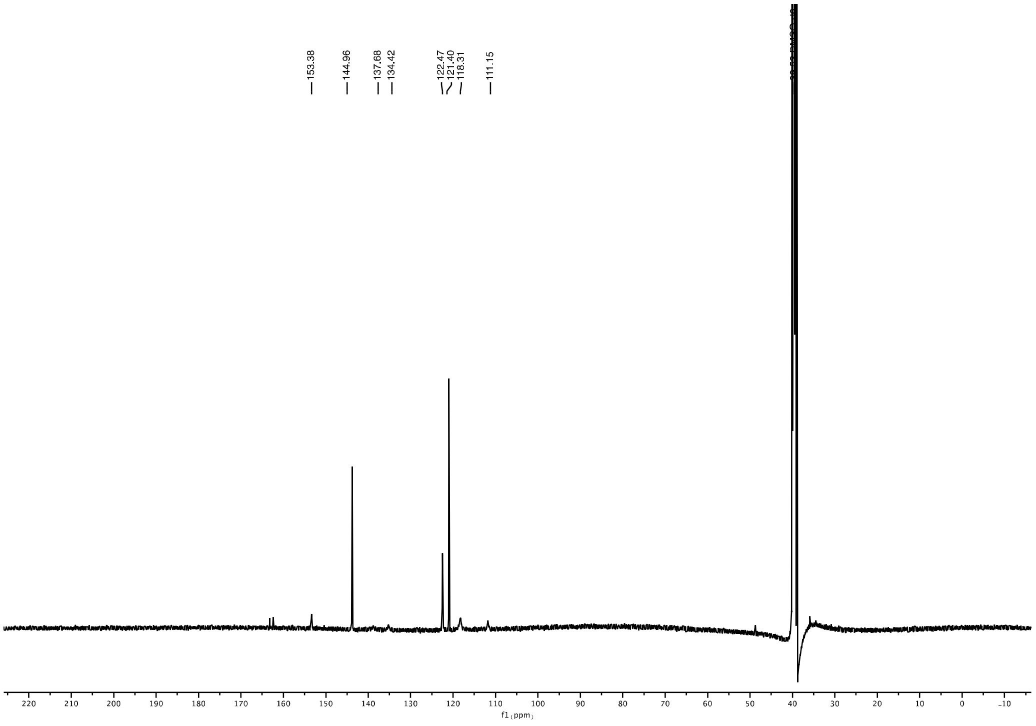
153.38
144.96
137.68
134.42
122.47
121.40
118.31
111.15
f1 (ppm)

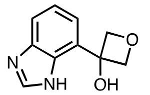

N
NH
O
OH

23

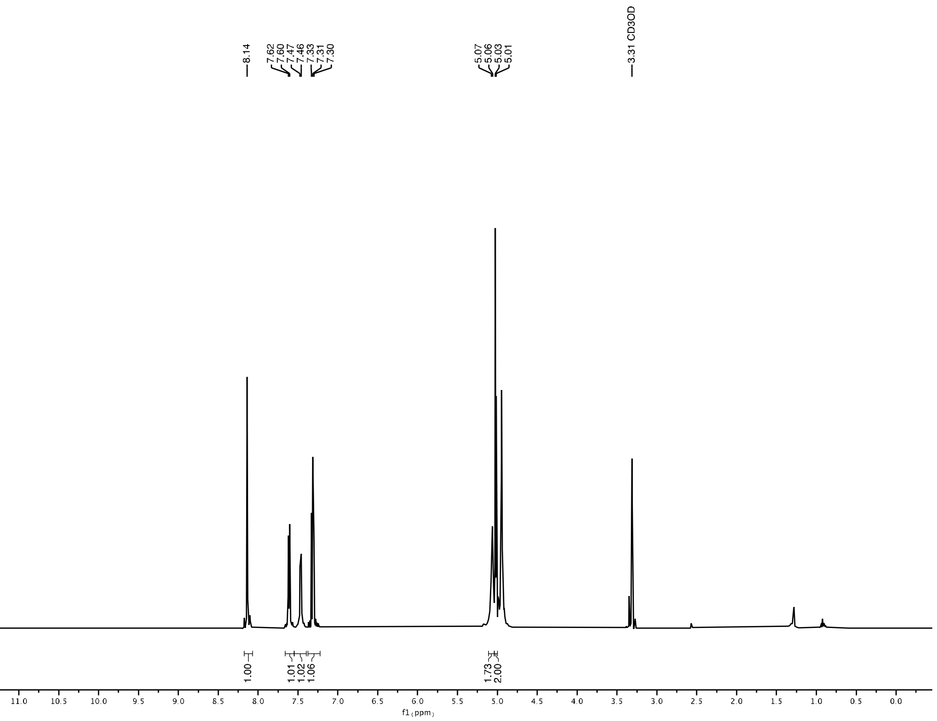

8.14
7.62
7.60
7.47
7.46
7.33
7.31
7.30
5.07
5.06
5.03
5.01
3.31 CD3OD
1.00
1.01
1.02
1.06
1.73
2.00
12.0 11.5 11.0 10.5 10.0 9.5 9.0 8.5 8.0 7.5 7.0 6.5 6.0 5.5 5.0 4.5 4.0 3.5 3.0 2.5 2.0 1.5 1.0 0.5 0.0
f1 (ppm)

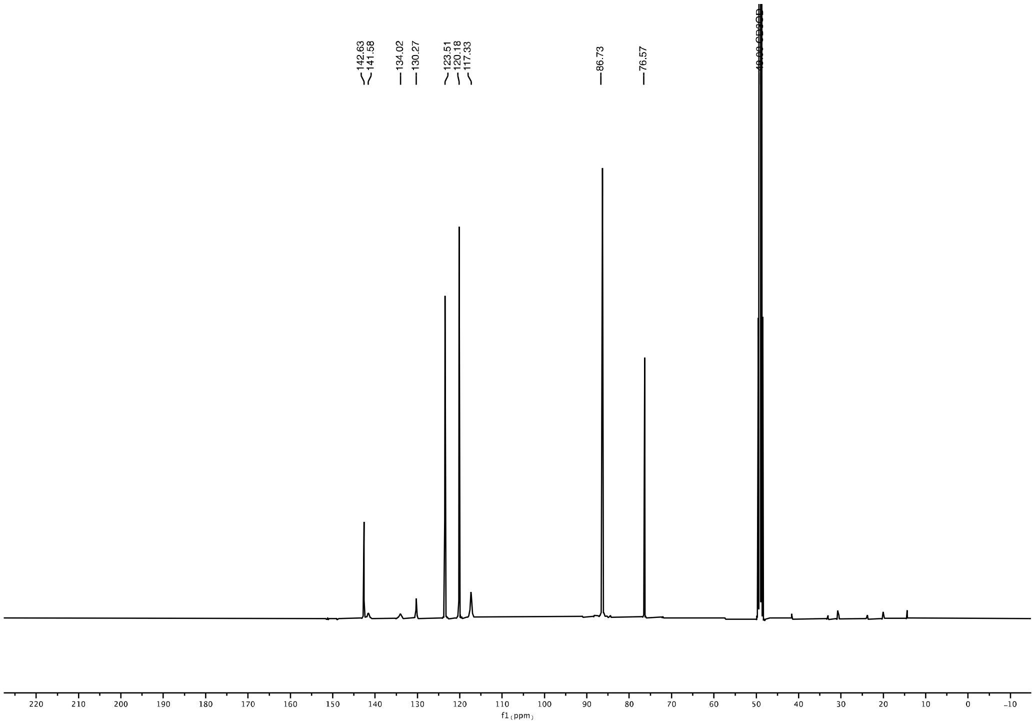

142.63
141.58
134.02
130.27
123.51
120.18
117.33
86.73
76.57
220 210 200 190 180 170 160 150 140 130 120 110 100 90 80 70 60 50 40 30 20 10 0 -10
f1 (ppm)

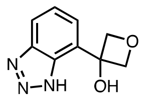

24

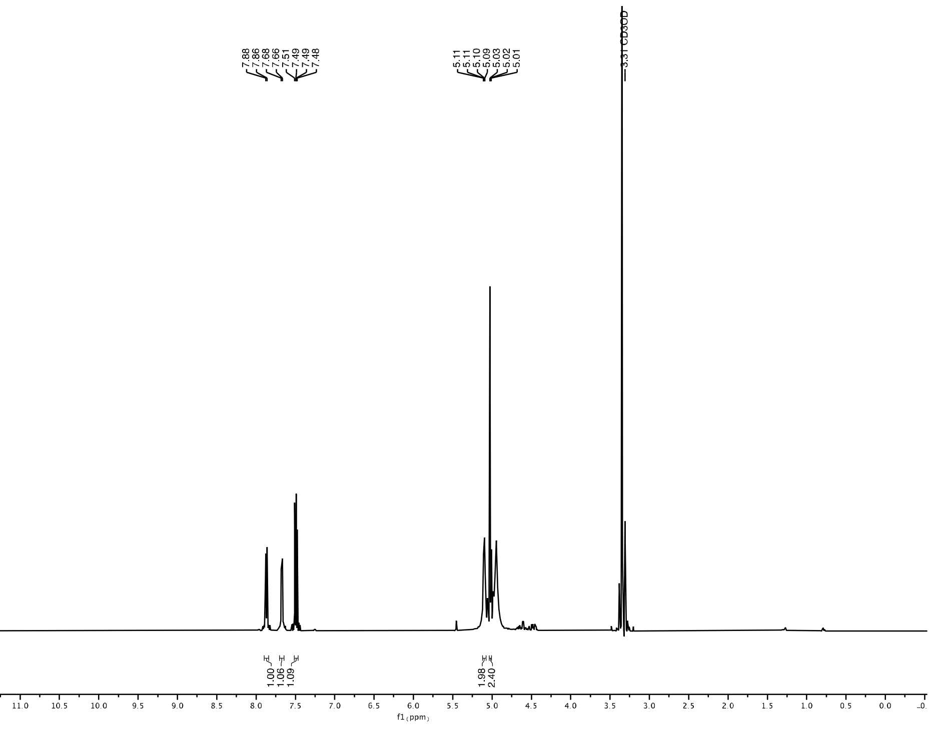

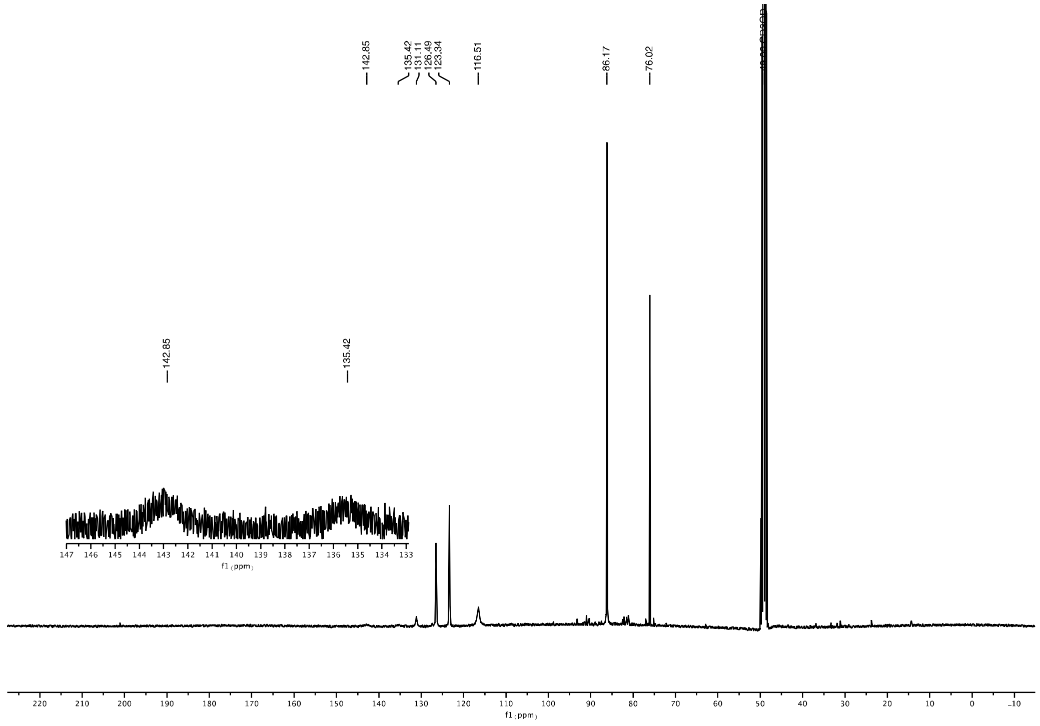

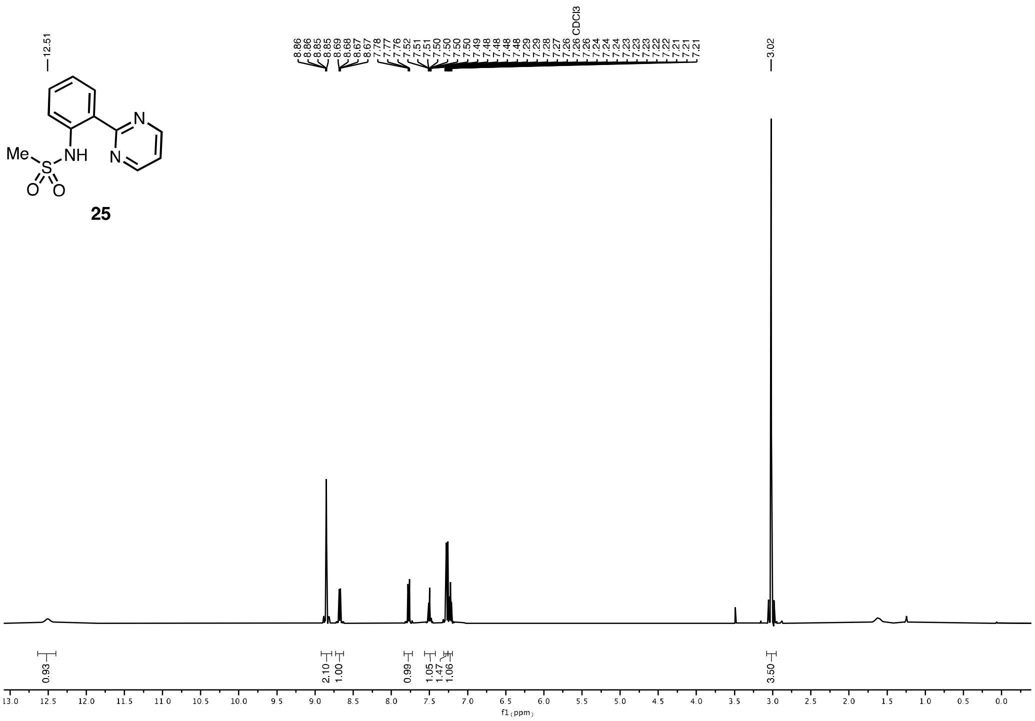
12.51
8.86
8.86
8.85
8.85
8.69
8.68
8.67
8.67
7.78
7.77
7.76
7.52
7.51
7.51
7.50
7.50
7.50
7.50
7.49
7.48
7.48
7.48
7.29
7.29
7.28
7.28
7.27
7.26 CDCl3
7.26
7.24
7.24
7.24
7.23
7.23
7.23
7.22
7.22
7.21
7.21
7.21
3.02
Me
NH
N
N
S
O
O
25
0.93
2.10
1.00
0.99
1.05
1.47
1.06
3.50
f1 (ppm)

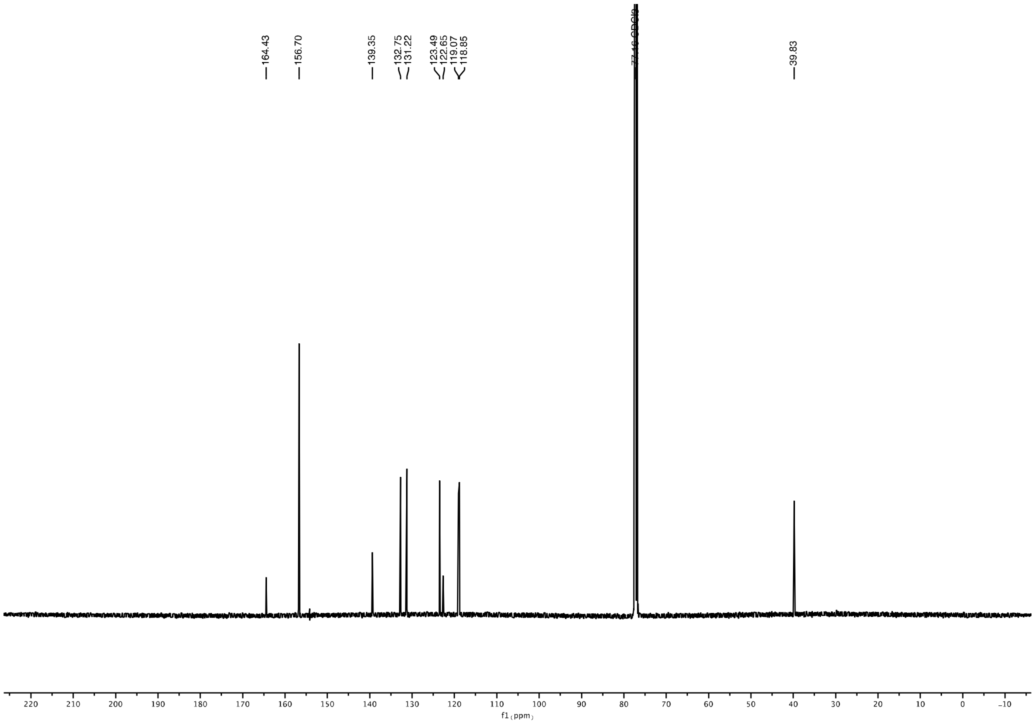
164.43
156.70
139.35
132.75
131.22
123.49
122.65
119.07
118.85
39.83
f1 (ppm)

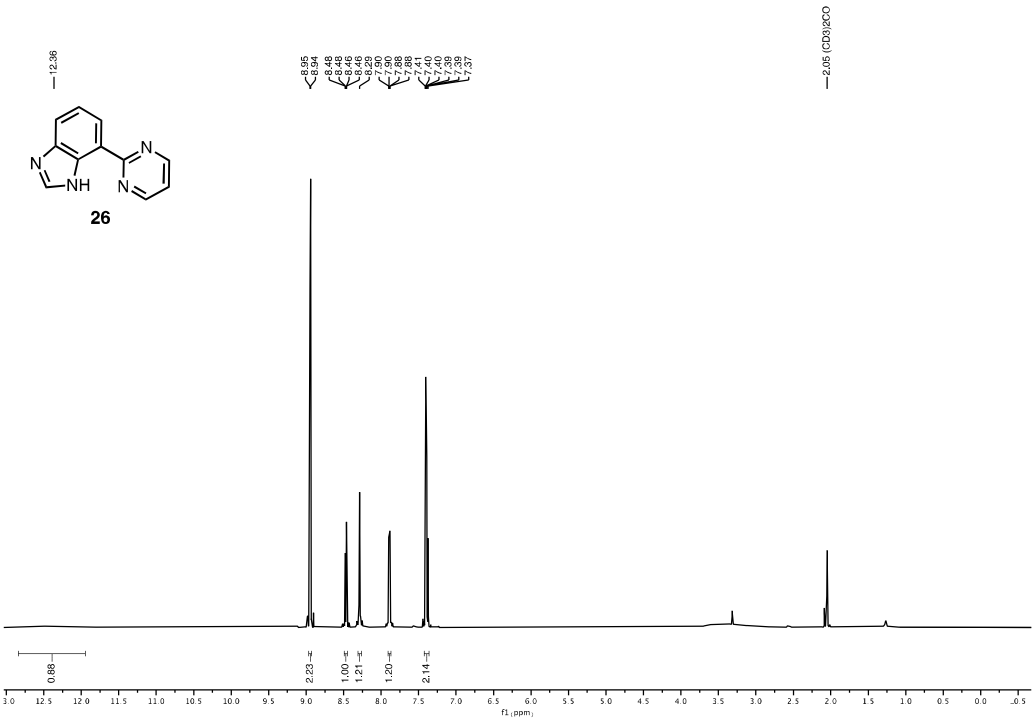

N
NH
N
N
26
12.36
8.95
8.94
8.48
8.48
8.46
8.46
8.29
7.90
7.90
7.88
7.88
7.41
7.40
7.40
7.39
7.39
7.37
2.05 (CD3)2CO
0.88
2.23
1.00
1.21
1.20
2.14
f1 (ppm)

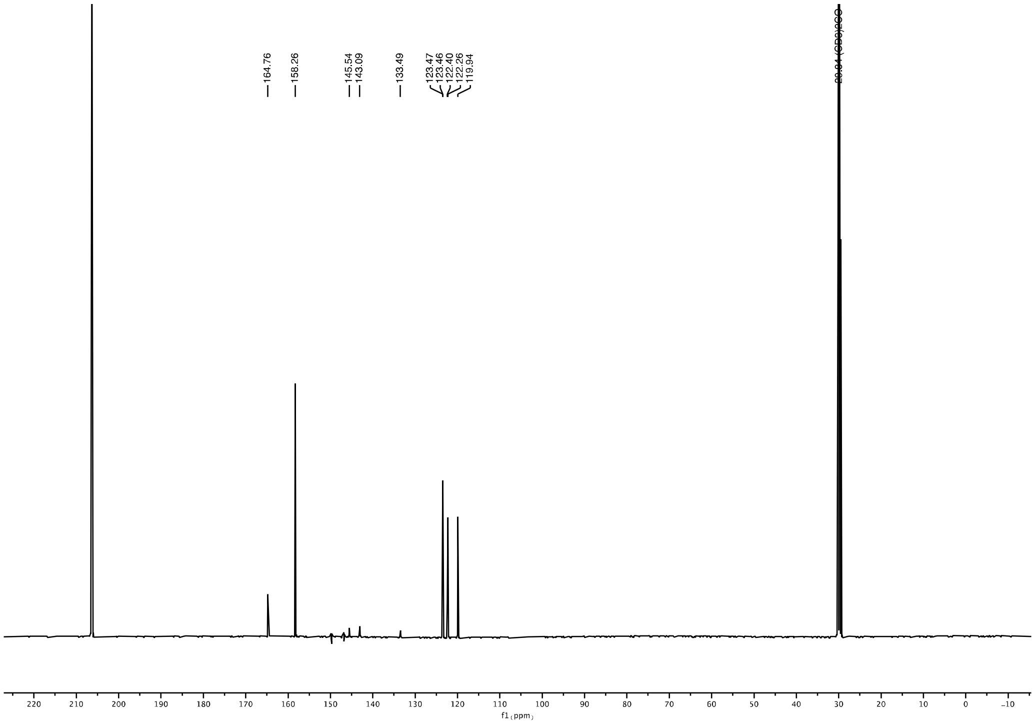

164.76
158.26
145.54
143.09
133.49
123.47
123.46
122.40
122.26
119.94
f1 (ppm)

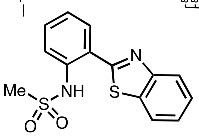

27

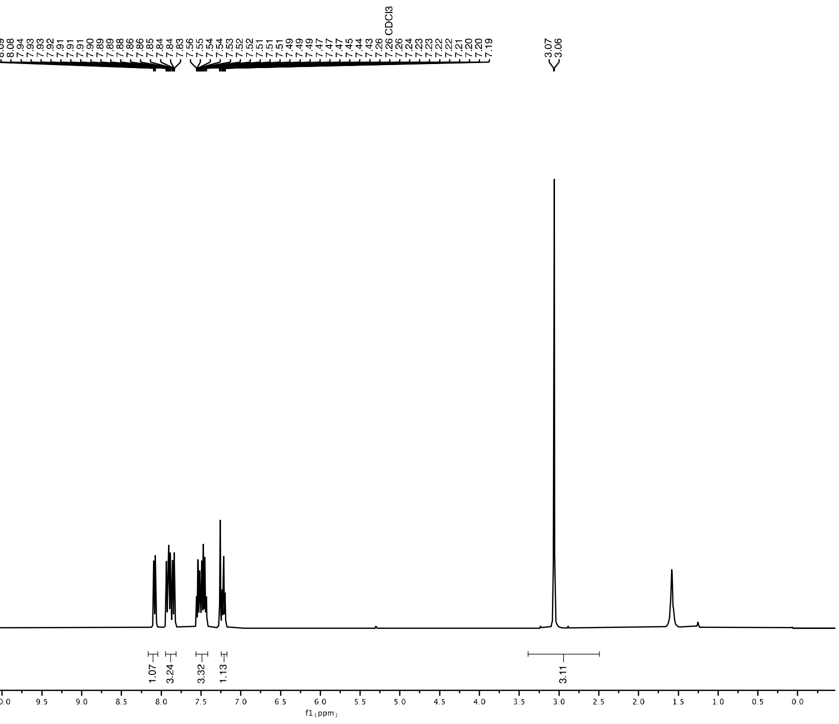
12.17
8.10 8.09 8.08 7.94 7.93 7.93 7.92 7.91 7.91 7.91 7.90 7.89 7.88 7.88 7.86 7.86 7.85 7.84 7.84 7.83 7.56 7.55 7.54 7.54 7.53 7.52 7.52 7.51 7.51 7.51 7.49 7.49 7.49 7.47 7.47 7.45 7.44 7.43 7.26 CDCl3 7.26 7.24 7.24 7.23 7.23 7.22 7.22 7.21 7.20 7.20 7.19
3.07 3.06
1.00
1.07
3.24
3.32
1.13
3.11
f1 (ppm)

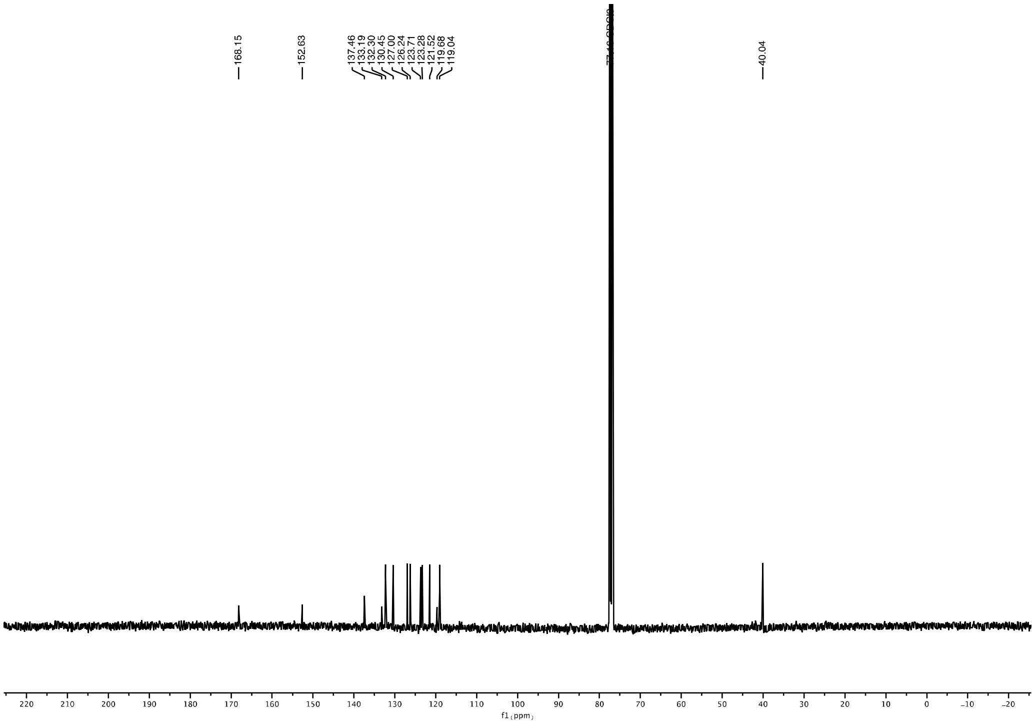
168.15
152.63
137.46 133.19 132.30 130.45 127.00 126.24 123.71 123.28 121.52 119.68 119.04
40.04
f1 (ppm)

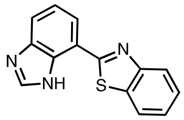
N
NH
S
N

28

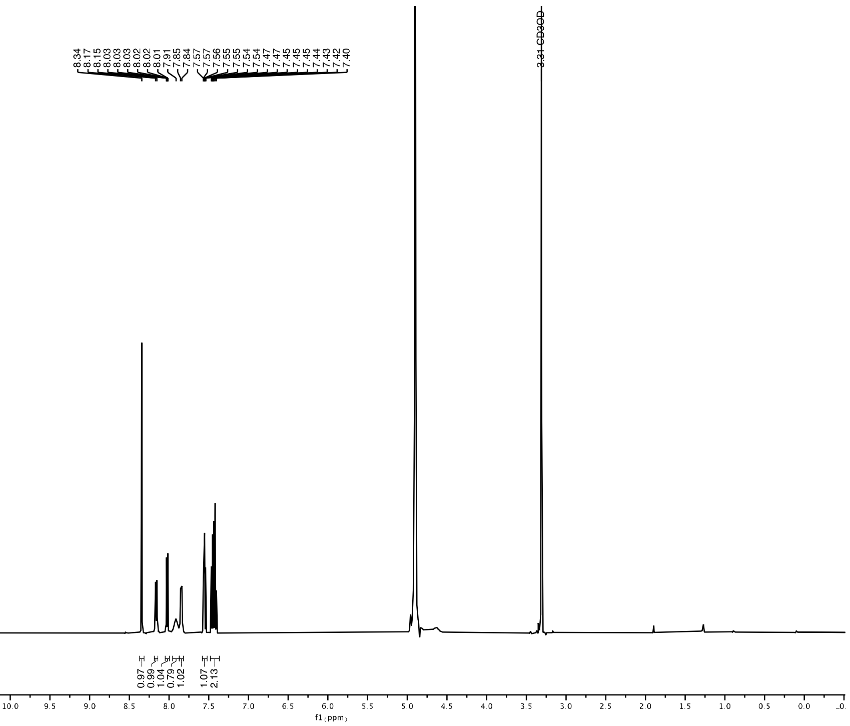
8.34
8.17
8.15
8.03
8.03
8.03
8.02
8.01
7.91
7.85
7.84
7.57
7.57
7.56
7.55
7.55
7.54
7.54
7.47
7.45
7.45
7.44
7.43
7.42
7.40
3.31 CD3OD
0.97
0.99
1.04
0.79
1.02
1.07
2.13
f1 (ppm)

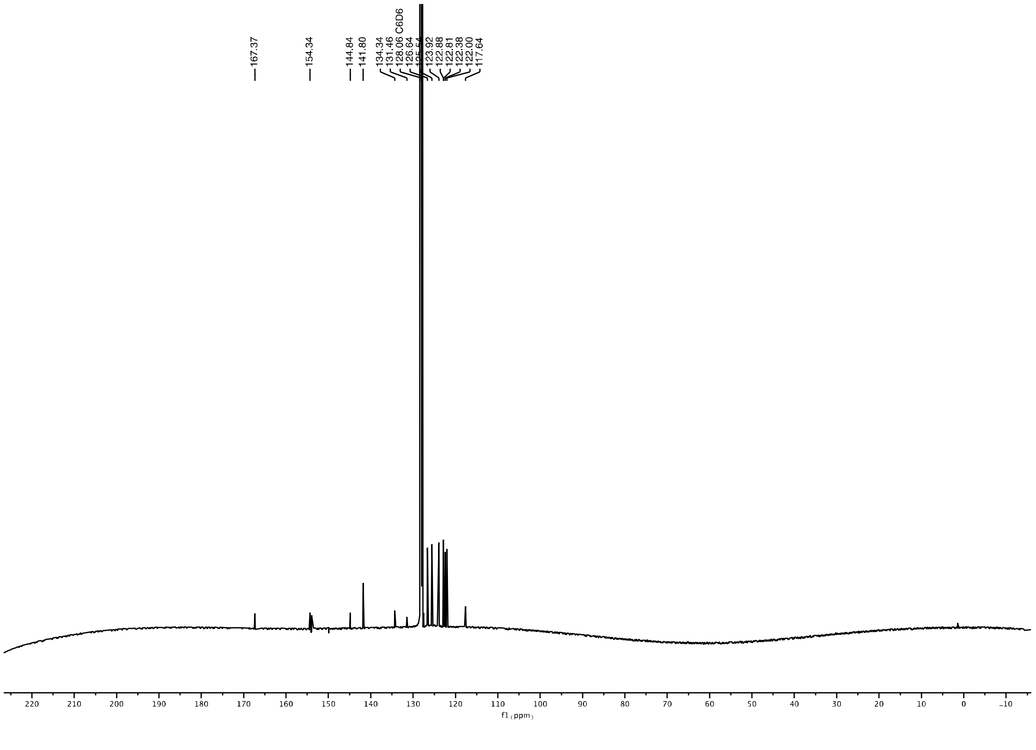
167.37
154.34
144.84
141.80
134.34
131.46
128.06 C6D6
126.64
123.92
122.88
122.81
122.38
122.00
117.64
f1 (ppm)

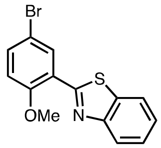

Br
S
N
OMe

s6

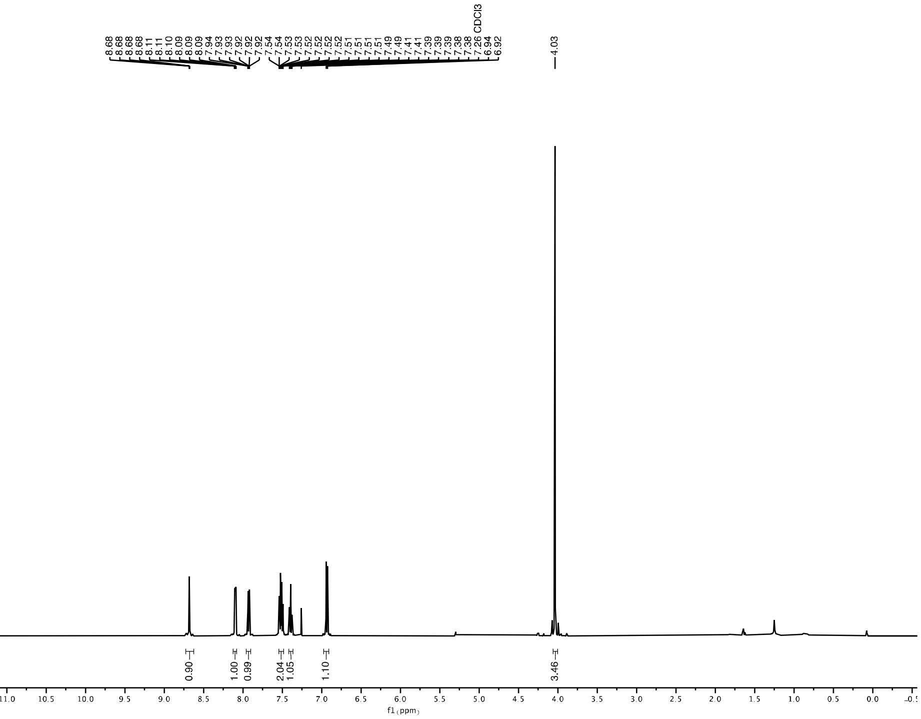

8.68 8.68 8.68 8.68 8.11 8.11 8.10 8.09 8.09 8.09 7.94 7.93 7.93 7.92 7.92 7.92 7.54 7.54 7.53 7.53 7.52 7.52 7.52 7.52 7.51 7.51 7.51 7.51 7.49 7.49 7.41 7.41 7.39 7.39 7.39 7.38 7.26 CDCl3 6.94 6.92
4.03
0.90
1.00
0.99
2.04
1.05
1.10
3.46
f1 (ppm)

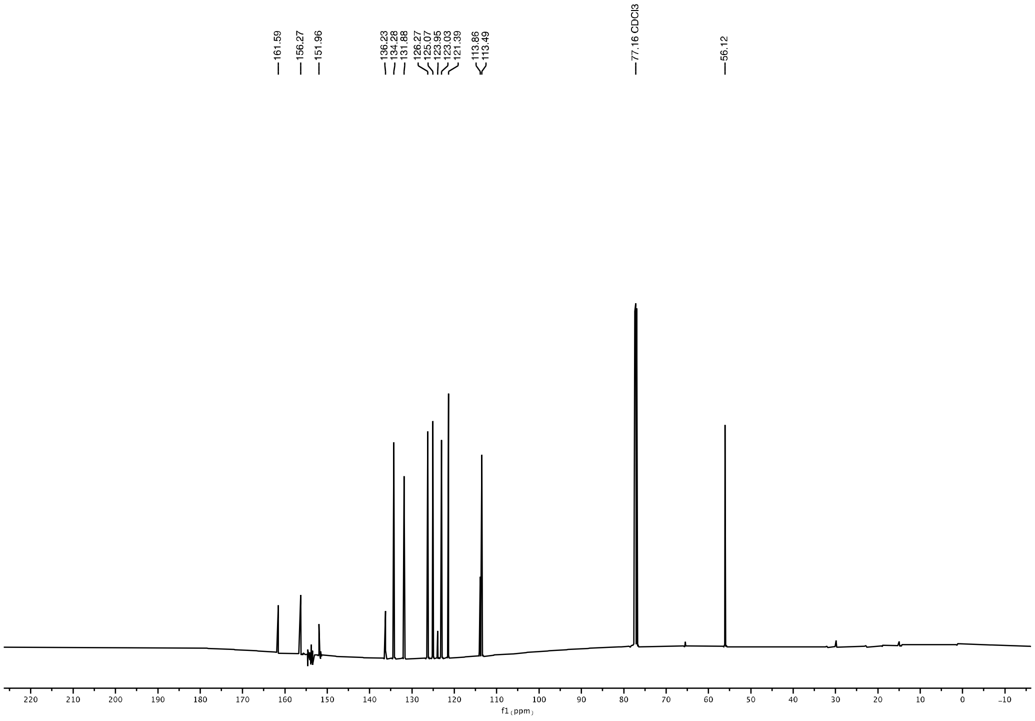

161.59
156.27
151.96
136.23
134.28
131.88
126.27
125.07
123.95
123.03
121.39
113.86
113.49
77.16 CDCl3
56.12
f1 (ppm)

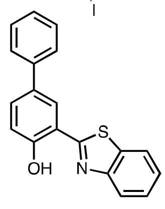

11a

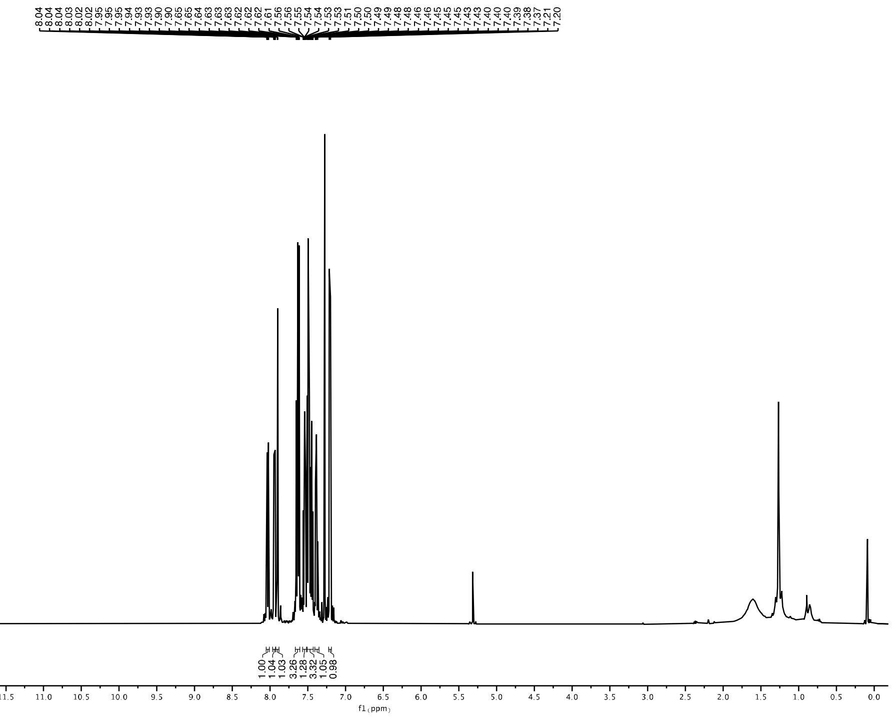

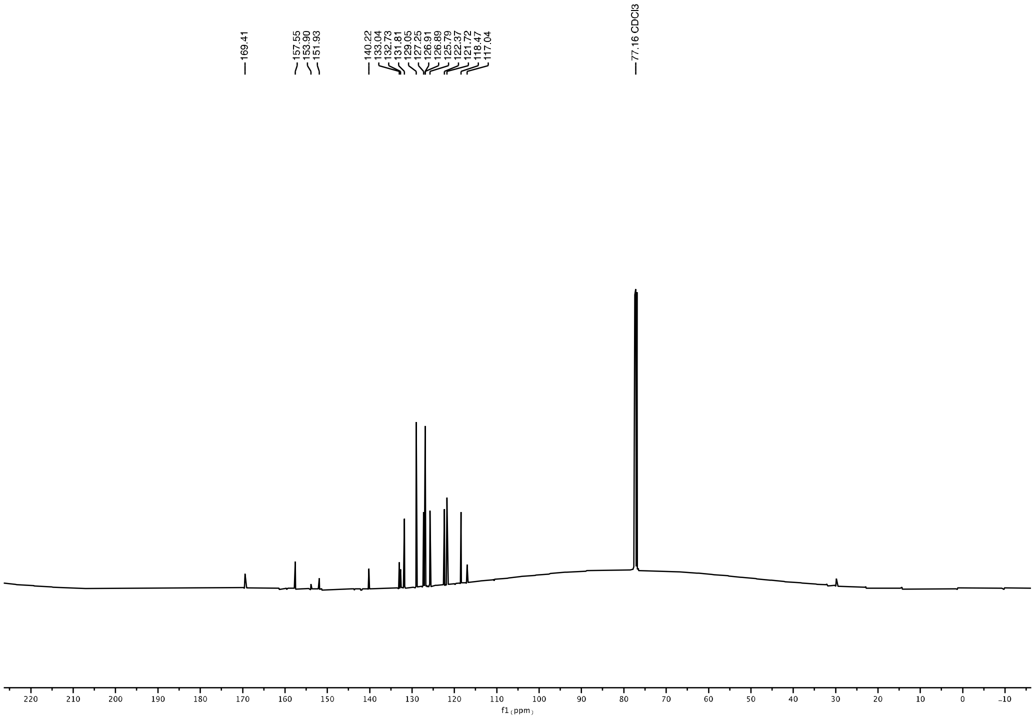

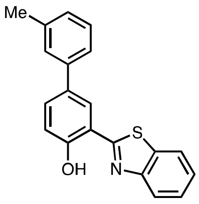
Me
OH
N
S

11b

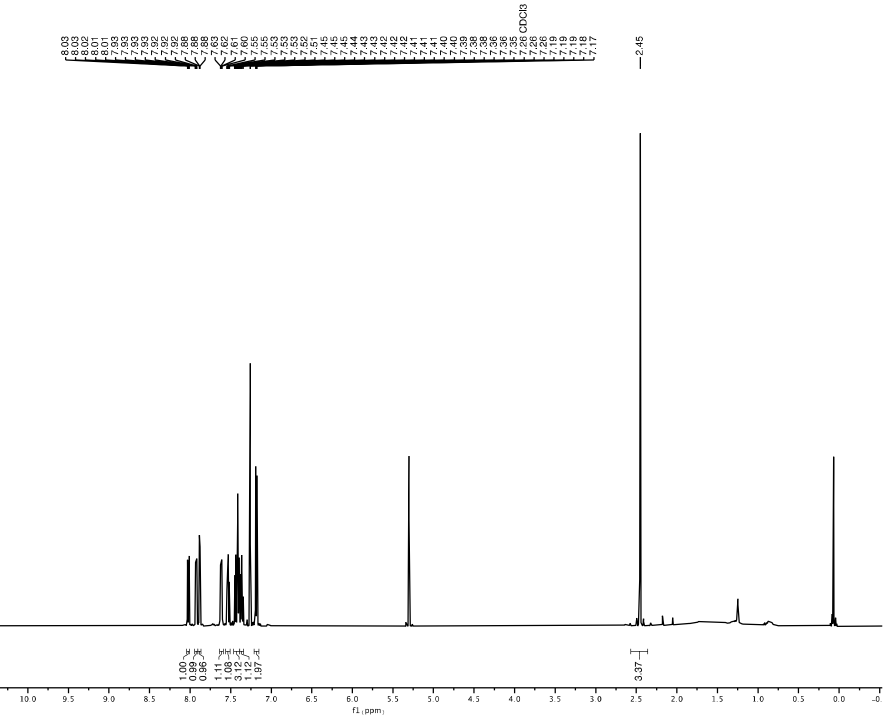
8.03
8.03
8.02
8.01
8.01
7.93
7.93
7.93
7.92
7.92
7.92
7.88
7.88
7.63
7.62
7.61
7.60
7.55
7.55
7.53
7.53
7.53
7.52
7.51
7.45
7.45
7.44
7.43
7.43
7.42
7.42
7.41
7.41
7.40
7.39
7.38
7.38
7.36
7.36
7.35
7.26 CDCl3
7.26
7.26
7.19
7.19
7.19
7.18
7.17
2.45
1.00
0.99
0.96
1.11
1.08
3.12
1.12
1.97
3.37
f1 (ppm)

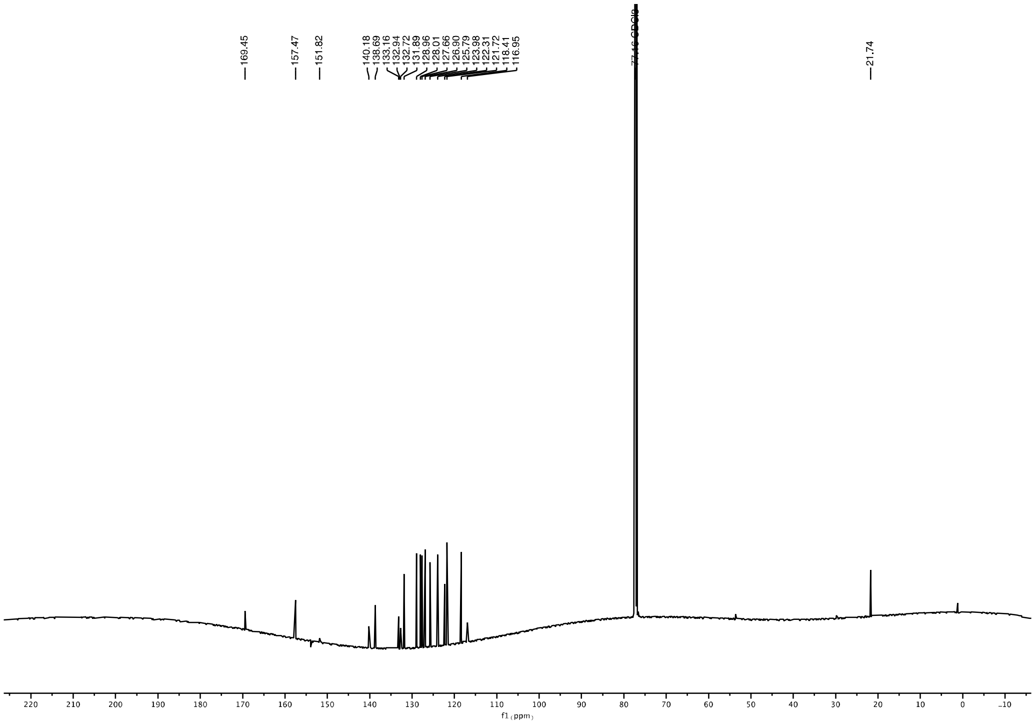
169.45
157.47
151.82
140.18
138.69
133.16
132.94
132.72
131.89
128.96
128.01
127.66
126.90
125.79
123.98
122.31
121.72
118.41
116.95
77.16 CDCl3
21.74
f1 (ppm)

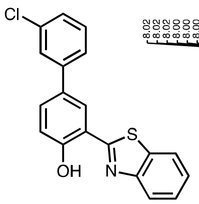

11c

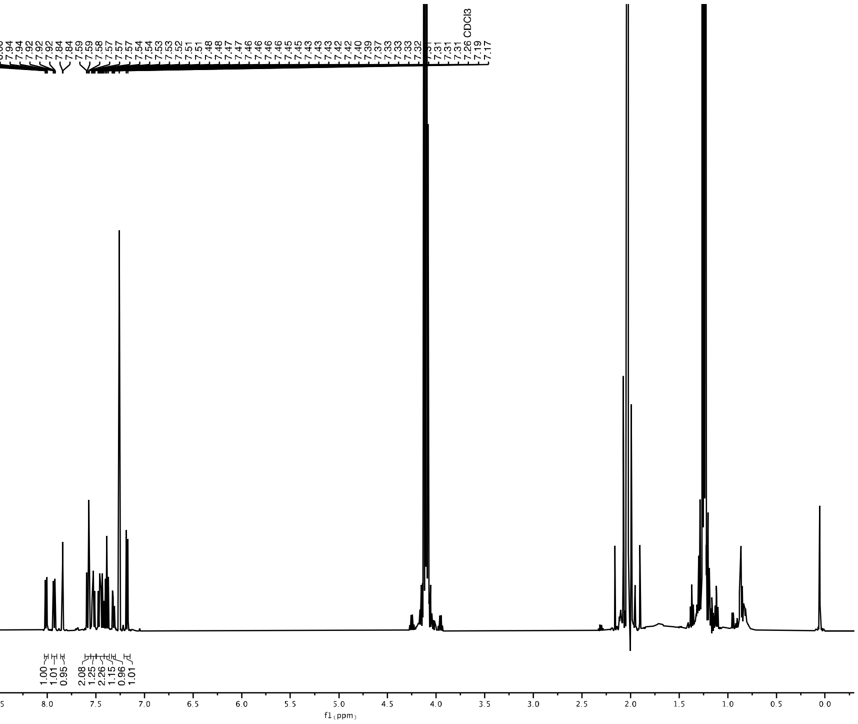

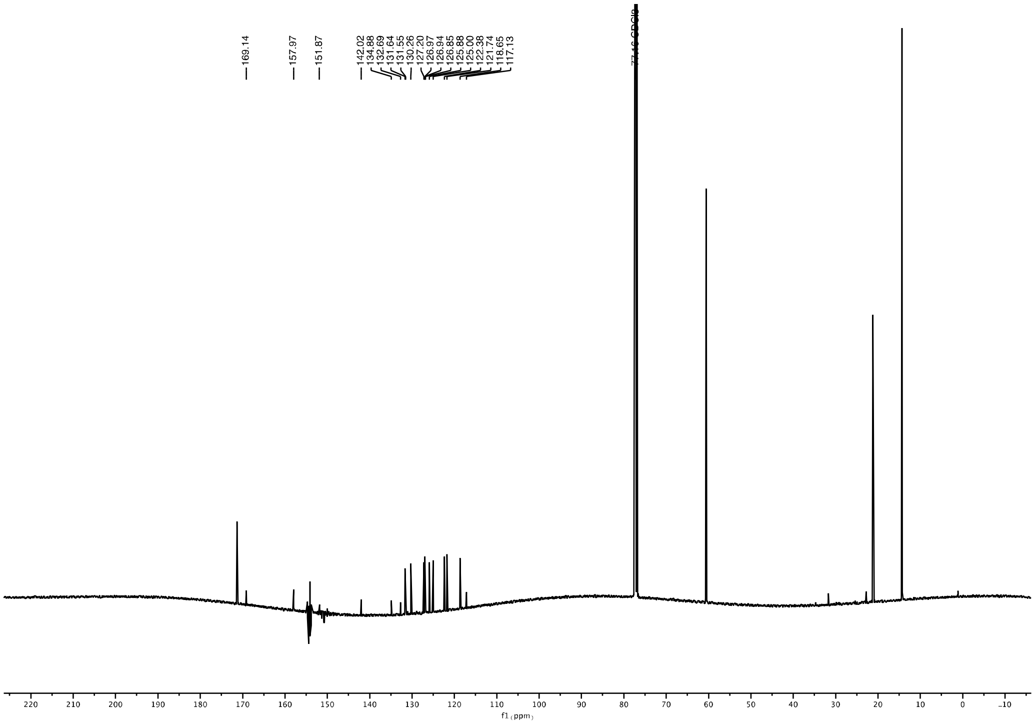

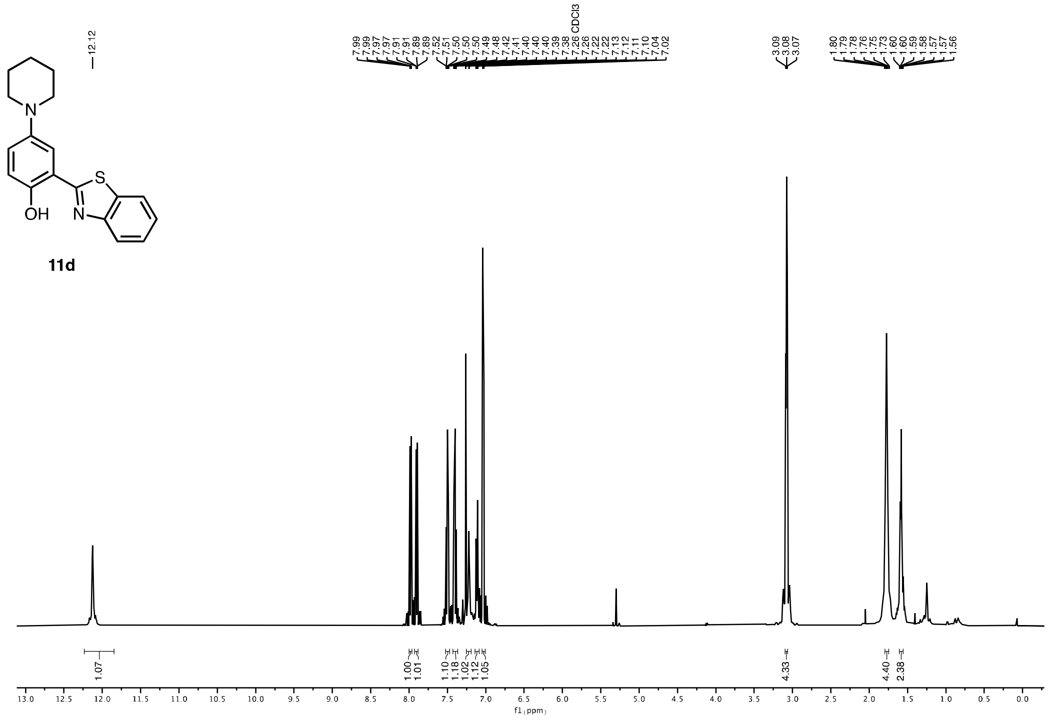

11d

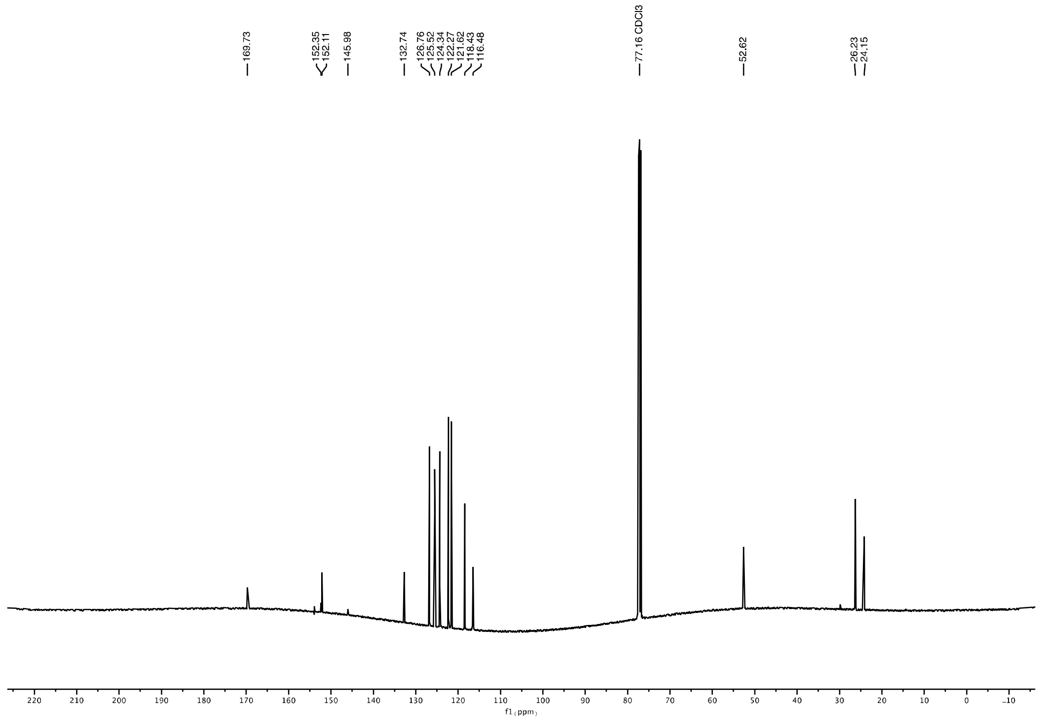

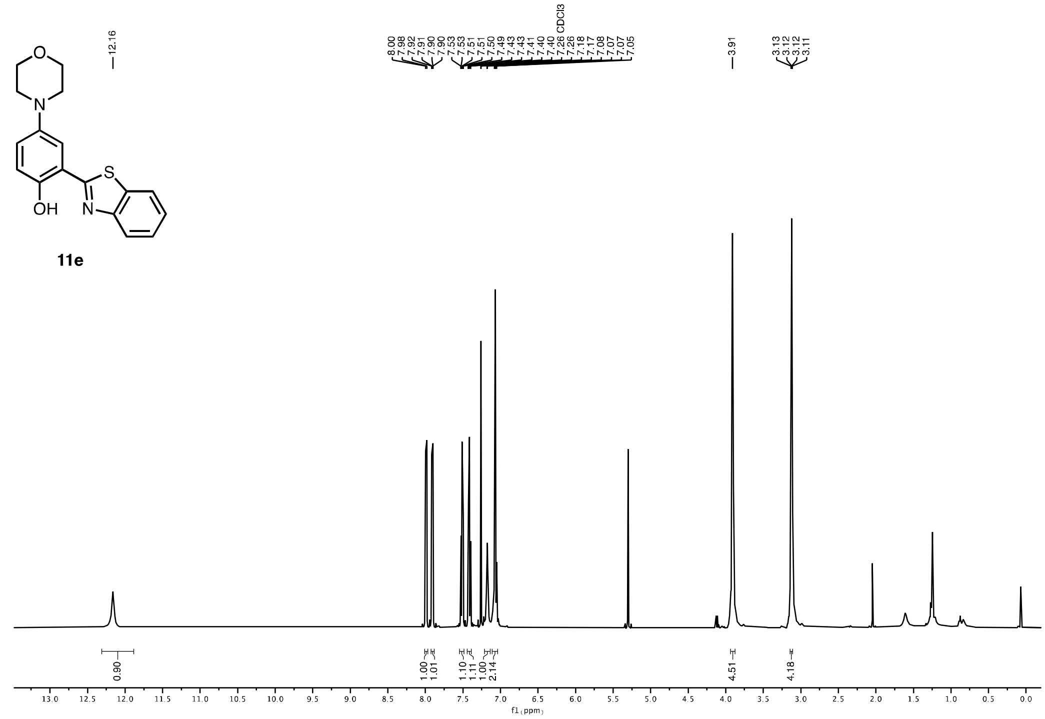
O
N
S
OH
N
12.16
8.00 7.98 7.92 7.91 7.90 7.90 7.53 7.51 7.51 7.50 7.49 7.43 7.41 7.40 7.40 7.26 CDCl3 7.26 7.18 7.17 7.08 7.07 7.07 7.05
3.91
3.13 3.12 3.12 3.11
0.90
1.00 1.01
1.10 1.11 1.00 2.14
4.51
4.18
f1 (ppm)

11e

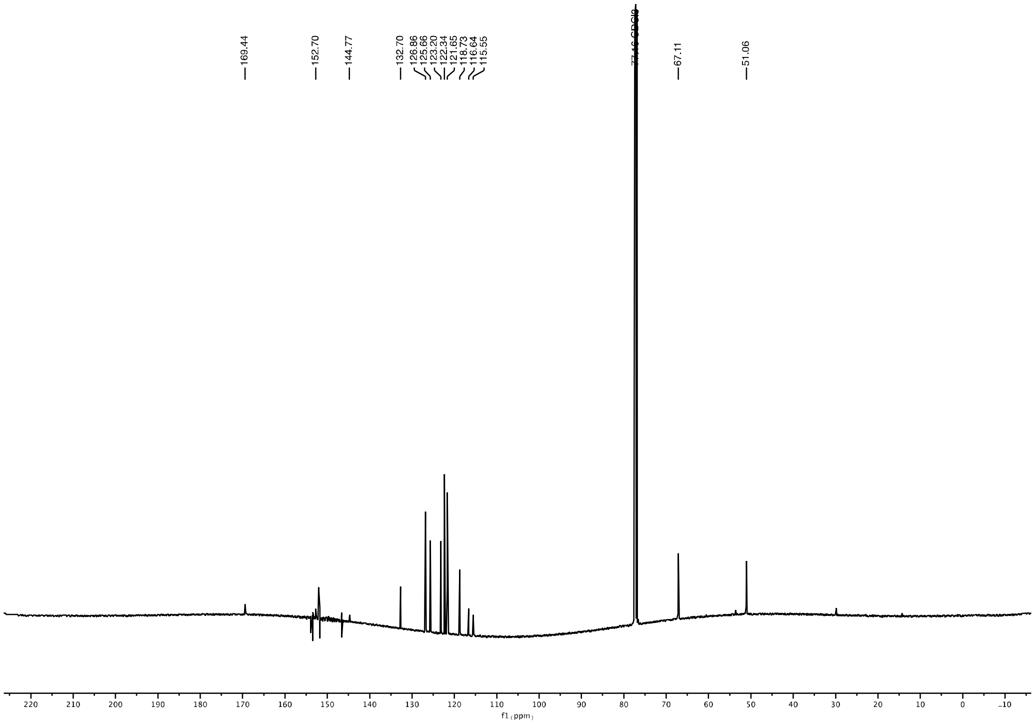
169.44
152.70
144.77
132.70 126.86 125.66 123.20 122.34 121.65 118.73 116.64 115.55
77.16 CDCl3
67.11
51.06
f1 (ppm)

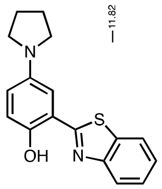
N
S
OH
N

11f

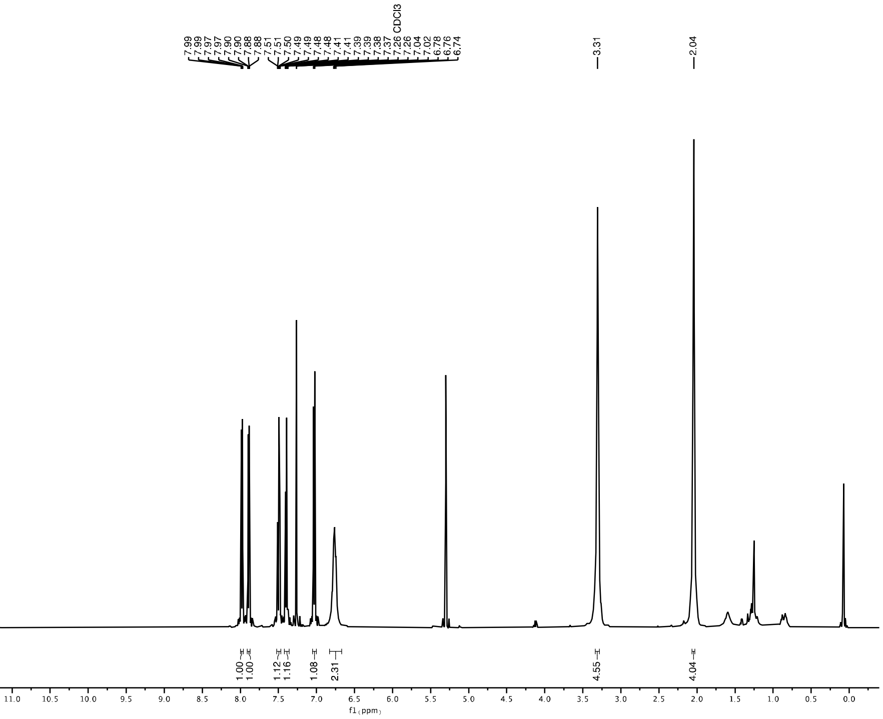
11.82
7.99
7.99
7.97
7.97
7.90
7.90
7.88
7.88
7.51
7.51
7.50
7.49
7.49
7.48
7.48
7.41
7.41
7.39
7.39
7.38
7.37
7.26 CDCl3
7.26
7.04
7.02
6.78
6.76
6.74
3.31
2.04
0.99
1.00
1.00
1.12
1.16
1.08
2.31
4.55
4.04
f1 (ppm)

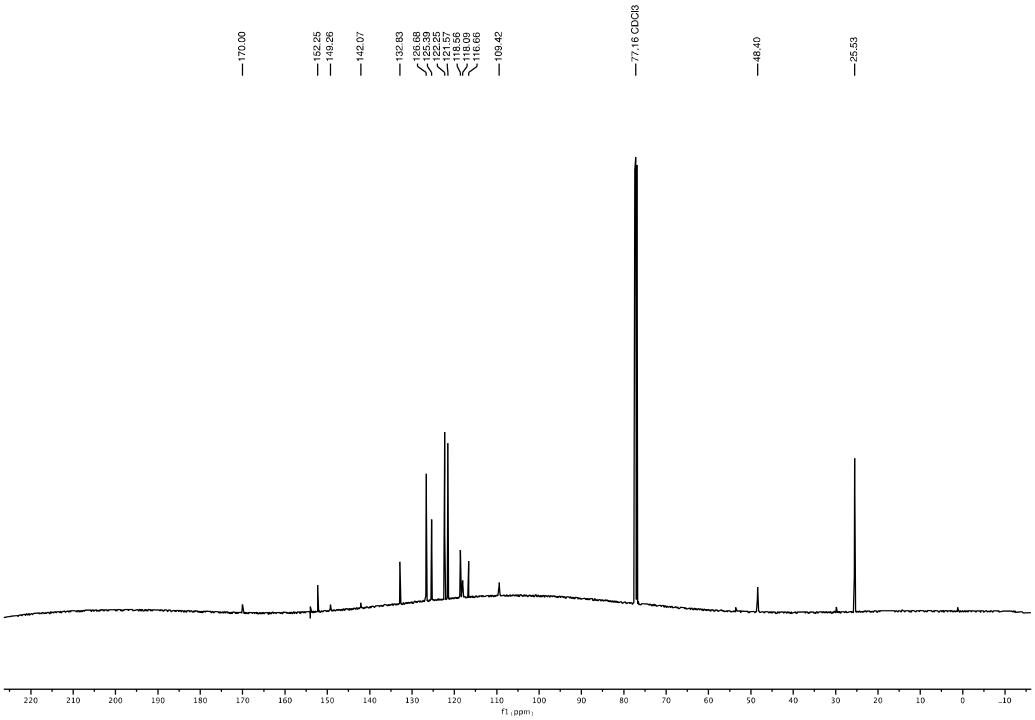
170.00
152.25
149.26
142.07
132.83
126.68
125.39
122.25
121.57
118.56
118.09
116.66
109.42
77.16 CDCl3
48.40
25.53
f1 (ppm)

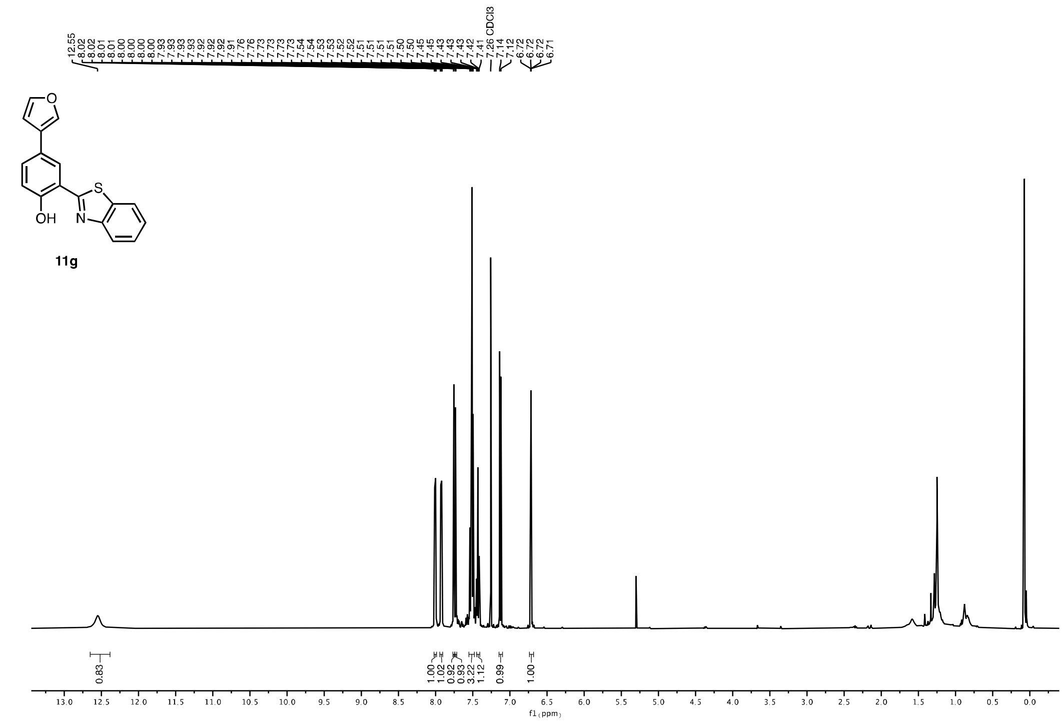

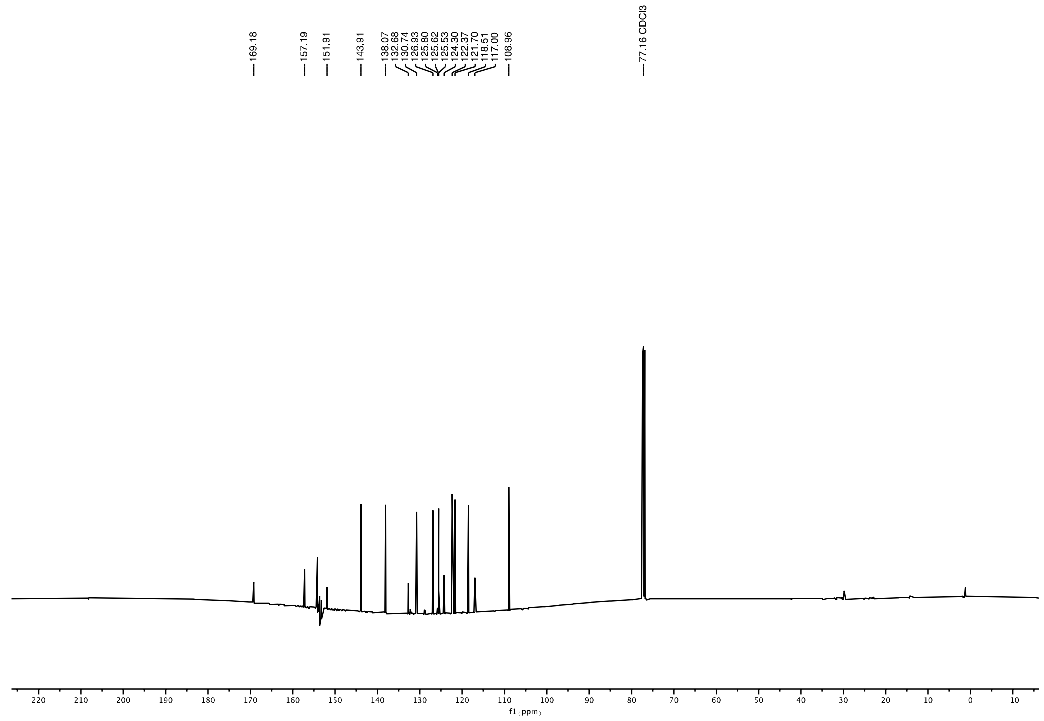

Supplement: SC-013-D1SC06011B-s003 [file SC-013-D1SC06011B-s003.pdf]
